# Multi-omics analysis provides new insights into the molecular mechanisms underlying colostral immunoglobulin G absorption in the gut of neonatal goat kids

**DOI:** 10.1016/j.aninu.2024.11.022

**Published:** 2025-01-25

**Authors:** Chao Yang, Yan Cheng, Tianxi Zhang, Kefyalew Gebeyew, Amanda Fischer-Tlustos, Leluo Guan, Michael Steele, Zhiliang Tan, Zhixiong He

**Affiliations:** a National Engineering Laboratory for Pollution Control and Waste Utilization in Livestock and Poultry Production, CAS Key Laboratory for Agro-Ecological Processes in Subtropical Region, South-Central Experimental Station of Animal Nutrition and Feed Science in Ministry of Agriculture, Hunan Provincial Engineering Research Center for Healthy Livestock and Poultry Production, Institute of Subtropical Agriculture, The Chinese Academy of Sciences, Changsha 410125, China; b State Key Laboratory of Plateau Ecology and Agriculture, Qinghai University, Xining 810016, China; c University of Chinese Academy of Sciences, Beijing 100049, China; d Department of Animal Biosciences, University of Guelph, Guelph ON N1G2W1, Canada; e Faculty of Land and Food Systems, The University of British Columbia, Vancouver BC V6T 1Z4, Canada; f Key Laboratory of Forage Breeding-by-Design and Utilization, Chinese Academy of Sciences, Beijing 100093, China

**Keywords:** Neonatal goat kid, Colostrum, Immunoglobulin G, Clathrin-mediated endocytosis, Macropinocytosis

## Abstract

Early colostrum feeding facilitates the passive transfer of immunoglobulin G (IgG), which contributes to the defensive establishment of neonates; however, the molecular mechanisms of IgG absorption in the small intestine of neonatal mammals remain largely unknown. In this study, a total of 16 neonatal goat kids with similar body weight (2.05 ± 0.31 kg) were selected and randomly assigned to 1 of 2 feeding treatments: normal colostrum feeding (NCF, *n* = 8) or delayed colostrum feeding (DCF, *n* = 8). Multi-omics coupled with individual bioinformatics analyses were employed to obtain a comprehensive understanding of the molecular mechanisms of IgG absorption. Phenotypic analysis showed that the capacity of IgG absorption was largely affected (*P* < 0.05) by colostrum feeding time in neonatal goat kids. Weighted gene co-expression network analysis generated 23 gene modules (gene module defined M1 to M23) and the M12 module was highly correlated (|r| > 0.70 and adjusted *P* < 0.01) with IgG absorption. Genes in M12 were involved in the endocytosis pathway, especially related to clathrin-mediated endocytosis and macropinocytosis. The differentially expressed genes (DEGs) enriched in the above-mentioned pathways regulated the clathrin synthesis (*CLTC*), the formation of clathrin-coated vesicles (*ARPC1A*), and the sorting and recycling endosomes (*CAPZA2*, *KIAA0196*, *RAB10*, *RAB11A* and *VPS35*) as well as the formation of macropinosomes (*FGFR4* and *RhoA*) in micropinocytosis, which induced differences in serum IgG concentrations. Additionally, 5 differentially expressed miRNAs (miR-2755-3p, miR-10400-5p, miR-71-5p, miR-2944-3p and miR-2411-3p) were predicted to regulate mRNA involved in clathrin-coated vesicles, Fc receptor for IgG (FcRn)-IgG sorting, and macropinosomes formation that may cause the difference in IgG absorption ability. This study provides new insights into the molecular mechanisms controlling IgG absorption of neonatal ruminants and reveals novel mRNA and miRNA markers involved in clathrin-mediated endocytosis and macropinocytosis which may provide the fundamental knowledge related to IgG absorption to support further study in other mammals.

## 1 Introduction

The prenatal and early postnatal periods are the vital windows of mammalian development during which nutritional factors can have profound and lasting effects on the growth and health of offspring (Langley-Evans, 2006). In humans, early breastfeeding is essential for infants due to both short and long-term beneficial effects, including preventing acute physical illnesses (Victora et al., 2000), promoting cognitive development (Kim and Choi, 2020) and modifying intestinal microbiology (Stewart, 2021). In other mammals, early suckling or colostrum feeding may promote growth and reproductive performance, as well as improve gut health in later life (Faber et al., 2005; Kao et al., 2020; Le Dividich et al., 2005; Soberon and Van Amburgh, 2011).

During the prenatal or early postnatal window, the establishment of passive immunity is critical to mammalian health. Maternal immunoglobulins can transfer through the placenta, yolk sac, or colostrum; all of which eventually ensure the establishment of immune defense and resistance to pathogen invasion in neonates (Godden, 2008). In humans, the fetus has direct contact with the maternal blood supply due to the hemochorial placenta's lack of maternal tissue layers, allowing antibodies, notably immunoglobulin G (IgG), to be prenatally transferred from mother to fetus (Borghesi et al., 2014). However, in ruminants, the synepitheliochorial placenta's lack of uterine epithelium leads to direct contact between maternal connective tissue and the chorion, which separates the maternal and fetal blood supplies and prevents the transfer of immunoglobulins during gestation (Borghesi et al., 2014; Godden, 2008). Thus, the sole mechanism responsible for passive immunity in neonatal ruminants is the uptake of large quantities of IgG from colostrum as soon as possible after birth. It is well known that optimal IgG absorption occurs within the first 4 h of life and rapidly declines after 12 h postpartum (Weaver et al., 2000). Compared to calves fed colostrum immediately after birth, delaying colostrum feeding to 6 and 12 h after birth reduced serum IgG levels and maximum apparent efficiency of absorption (AEA) of IgG (Fischer et al., 2018). All the aforementioned evidence indicates that the capacity of IgG absorption across small intestinal epithelium tremendously depends on colostrum feeding time. However, knowledge related to the potential regulatory mechanisms of how colostrum feeding time affects the process of IgG absorption is limited.

Generally, IgG absorption in small intestinal epithelium is accomplished through transcytosis, in which the Fc receptor for IgG (FcRn) plays a vital role (Rodewald and Kraehenbuhl, 1984). FcRn belongs to the functionally distinct family of major histocompatibility complex (MHC) molecules and consists of a type I transmembrane MHC class I-related heavy α chain that non-covalently binds to a β2-microglobulin light chain (Roopenian et al., 2003). FcRn is the only receptor that can bind to IgG with high affinity at low pH and traffic IgG in both directions across intestinal epithelium to participate in host defense (Claypool, 2016; Claypool et al., 2004). The pathway for FcRn-mediated transcytosis of IgG has been widely verified in human intestinal epithelial cells, and the first cellular event of this process is IgG bound to FcRn located on the apical enterocyte surface membrane (Dickinson et al., 1999). The complex of IgG and FcRn is then internalized by endocytosis and diverted into the early endosome and the common recycling endosome. After that, the acidification of endosomes activates the FcRn-mediated transport of IgG to the basolateral membrane where the neutral extracellular pH induces the release of IgG into circulation (He et al., 2008). Contrary to the above-mentioned studies, some data have revealed that abundant IgG was observed in FcRn^−/−^ neonatal rodent enterocytes, suggesting that IgG uptake took place in the apical surface of the enterocytes without the assistance of FcRn (Mohanty et al., 2013). The previous study documented that FcRn and IgG were internalized into endosomes by endocytosis, then the binding of FcRn and IgG occurred in acidic endosomes, and finally, IgG was transported across the epithelial-cell barrier to neonatal circulation (Roopenian and Akilesh, 2007). Nevertheless, the potential molecular mechanism of IgG absorption by transcytosis including initiation of internalization, endosomal formation and maturation in infants and other animals, especially in ruminants, still warrants elucidation.

In rodents, IgG absorption mainly occurs in the proximal jejunum (Mohanty et al., 2013), and similar results occurred in the neonatal goat kids that IgG vacuoles were only observed in the jejunal villus (Nordi et al., 2012). To elucidate the potential regulatory mechanisms involved in IgG absorption, this study utilized goat kids as an animal model and selected proximal jejunum to conduct next-generation sequencing of transcriptomes (RNA-Seq sequencing of mRNAs and microRNAs) using integrated bioinformatics analyses coupled with phenotypic values to investigate the candidate genes or pathways that mediate IgG transportation. A preliminary understanding of the regulatory mechanism of IgG absorption may give new insight into factors that induce differences in IgG absorption with respect to feeding time. Furthermore, it will also provide the fundamental knowledge related to the molecular mechanism of IgG absorption to support further study in other mammals.

## 2 Materials and methods

### 2.1 Animal ethics statement

The animal feeding trial was conducted from March 2019 to June 2019 at a local commercial farm (Jiangxi Mulei Agriculture and Forestry Development Co. Ltd.) located in the Jiangxi Province of China. The use of animals and all experimental protocols for the current study were approved by the Animal Care and Use Committee of the Institute of Subtropical Agriculture, Chinese Academy of Sciences (permit No. ISAZLT1805).

### 2.2 Colostrum preparation

Due to the low yield of colostrum and the difficulty in collecting enough colostrum from Ganxi black goats, this study used dairy cow colostrum replacing goat colostrum. It is reported that the use of bovine colostrum in small ruminants (especially goats) as an alternative colostrum resource is feasible, bovine colostrum can be well absorbed by neonatal goats (Yang et al., 2022). Colostrum used in the current study was obtained from five multiparous dairy cows within 12 h after parturition at a local dairy farm (Hunan Youzhuo Animal Husbandry Co., Ltd., Changsha, China). Colostrum collected from each cow was pooled together, aliquoted into 50 mL sterile centrifuge tubes, and stored at −20 °C until used for animal feeding. The gross composition of colostrum was determined using a Milk Analyzers platform (FOSS Electric, Hilleroed, Denmark). The contents of total solids, protein, fat, lactose, and urea in the bovine colostrum were 23.50%, 15.67%, 4.15%, 3.48%, and 28.70 mg/dL, respectively. Approximately 5 mL of colostrum was centrifuged at 3000 × *g* for 15 min at 4 °C to obtain supernatant for IgG determination using commercial bovine-specific ELISA kits (Jiangsu Meimian Industrial Co., Ltd., Jiangsu, China), which determined that the IgG concentration of colostrum fed to goat kids was 15.43 mg/mL.

### 2.3 Animals and experimental design

**Fig. 1:**
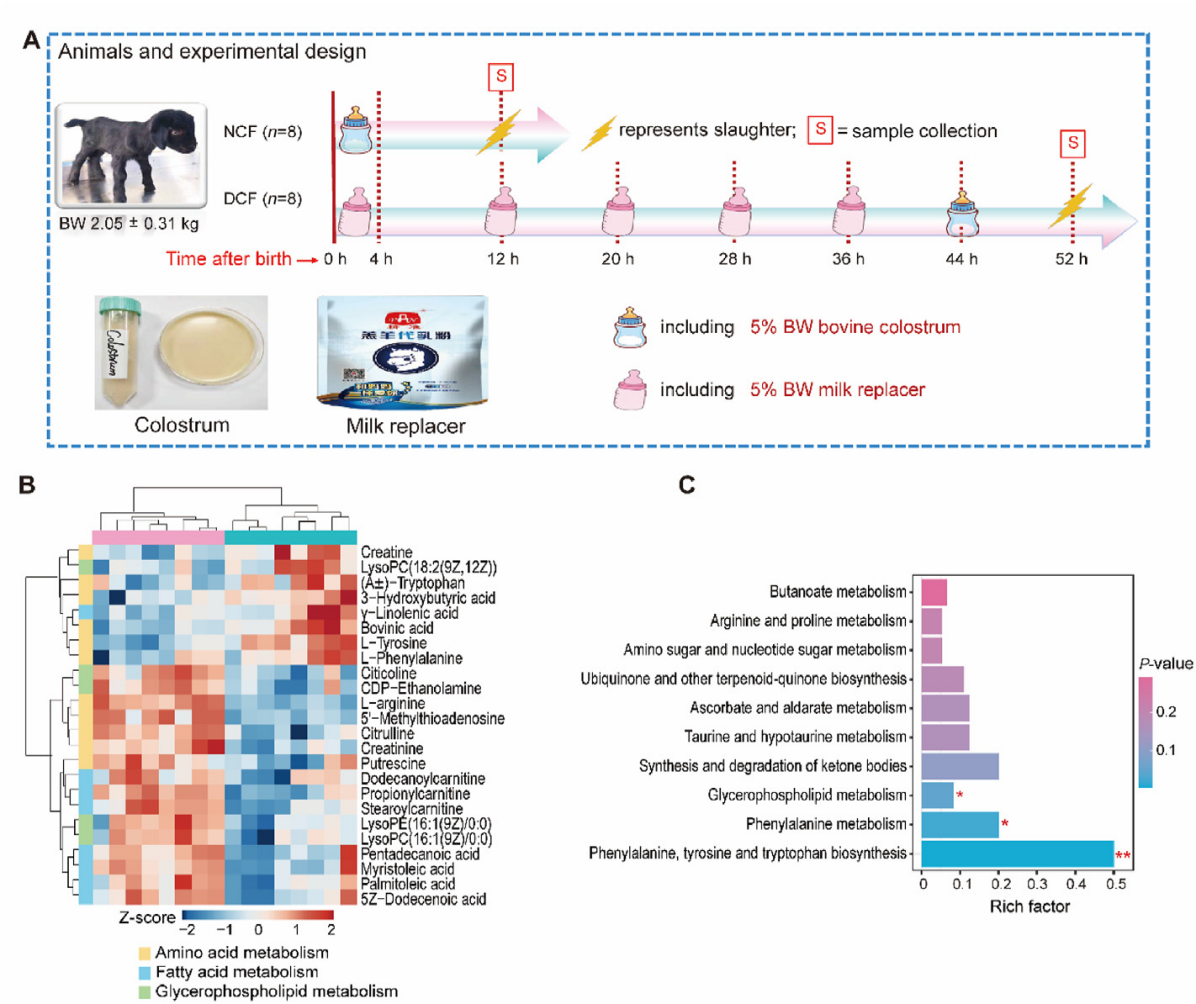
Experimental design and metabolomic profiles of jejunal tissue in neonatal goat kids. (A) Experimental design, feeding regime and the information of sample collection. (B) Heatmap of differential metabolites related to amino acid metabolism, fatty acid metabolism and glycerophospholipid metabolism. (C) KEGG pathway enrichment analysis of the union set of differential metabolites in PI and NI mode. Rich factor represents the ratio of the number of differentially expressed genes annotated in a pathway to the number of all genes annotated in the pathway. ∗ indicates *P* < 0.05 and ∗∗ indicates *P* < 0.01. NCF = normal colostrum feeding; DCF = delayed colostrum feeding; BW = body weight; PI = positive ion; NI = negative ion; KEGG = The Kyoto Encyclopedia of Genes and Genomes.

Information regarding the experimental animal and design is illustrated in Fig. 1A. A total of 16 neonatal goat kids with similar body weight (BW) (2.05 ± 0.31 kg) were selected and randomly assigned to 1 of 2 feeding treatments: normal colostrum feeding (NCF) or delayed colostrum feeding (DCF). It is reported that the process of IgG absorption from colostrum in neonatal ruminants is the greatest within 6 h of birth, decreases after 6 h and ends after 24 or 48 h due to “gut closure” (Lorenz et al., 2011; Nordi et al., 2012). Thus, in the current study, colostrum feeding time for neonatal goat kids in the NCF (within the first 4 h from birth, all goats were mainly fed colostrum at 2 to 2.5 h after birth) and DCF (at 44 h from birth) groups represented before and after gut closure, respectively. Specifically, kids in the NCF and DCF groups were fed colostrum at 5% BW and milk replacer at 5% BW within the first 4 h after birth, respectively. The DCF group received a 5% BW milk replacer every 8 h until 44 h when the kids were fed their first meal of colostrum at 5% BW. Milk replacer was prepared by mixing 200 g of milk replacer powder in 1 L of water, and the milk replacer powder (Beijing Precision Animal Nutrition Research Center, Beijing, China) contained 94% dry matter (DM), 23% crude protein (CP), 12% ether extract (EE), 3% crude fiber (CF), 10% ash, 1.5% calcium, and 1.2% phosphorus. Briefly, the DM of milk replacer powder was determined according to the national standard of China (GB/T 6435-2014). The CP concentration was determined via national standard of China (GB/T 6432-2018) using an automatic nitrogen apparatus (Kjeltec 8400, FOSS, Denmark). The concentration of EE was determined via national standard of China (GB/T 6433-2006) using a Soxhlet apparatus extractor (DW-SXT-06, Drawell, Chongqing, China). The content of CF was determined by the national standard of China (GB/T 6434-2022) using an automatic fiber analyzer (Fibretherm FT12, Gerhardt, Nordrhein-Westfalen, Germany). Ash was determined by national standard of China (GB/T 6438-2007) using a muffle furnace (K114, Thermo Fisher, Massachusetts, USA). Calcium and phosphorus were determined by national standards of China (GB/T 6436-2018 and GB/T 6437-2018) using an inductively coupled plasma emission spectrometer (5110 ICP-OES, Agilent, California, USA).

Newborn goat kids were immediately removed from their dams to avoid suckling, weighed to determine the BW, and then housed separately in individual hutches (1.2 m × 1 m × 1.2 m). Before the formal experiment, all hutches underwent cleaning and disinfection. During the experimental period, all neonatal goat kids had free access to water and each hutch was equipped with a warming light. To ensure equivalent IgG absorption time (8 h) from colostrum feeding, goat kids in the NCF and DCF groups were slaughtered at 12 and 52 h after birth, respectively. No goats received vaccine or other drug injections during the experimental period.

### 2.4 Sample collection

Blood samples from each goat in the NCF and DCF groups were collected from the jugular vein before slaughter, and then serum was separated at 3000 × *g* at 4 °C for 15 min. Afterward, goat kids were euthanized by exsanguination after intravenous administration of anesthesia (50 mg/kg BW) and then the small intestine was separated according to the previous method (Malmuthuge et al., 2015). In detail, jejunal tissues were the proximal 30 cm portion of the jejunum. Then, jejunum tissue was thoroughly washed with 0.9% saline solution, chipped into small pieces, and then placed into a sterile sample bag (B01064, Whirl-Pak, WI, USA), frozen in liquid nitrogen and stored at −80 °C.

### 2.5 Analysis of serum IgG concentration and the maximum AEA of IgG

Serum IgG concentration was analyzed using a commercial goat-specific ELISA kit (Jiangsu Meimian Industrial Co., Ltd., Jiangsu, China) according to the manufacturer's instructions. The AEA of IgG for each goat was calculated using goat BW, goat serum IgG concentration, colostrum IgG concentration and colostrum feeding volume. The assumed blood plasma volume of neonatal goat kids was equivalent to 7.5% of BW and the equation was described by Ramos et al. (2010):

AEA (%) = [(BW × 0.075) × serum IgG concentration/(colostrum IgG concentration × colostrum feeding volume)] × 100.

### 2.6 Analysis of jejunal IgG level

The jejunal homogenate of each goat kid was prepared using approximately 200 mg of jejunal tissue and 1.8 mL of phosphate buffer solution in a ratio of 1:9. The grinding process was conducted using a handheld glass homogenizer and a vortex mixer (Vortex-Genie G560E, Scientific Industries, New York, USA). Then, the homogenate was centrifuged at 12,000 × *g* for 15 min at 4 °C to obtain supernatant and the IgG level of that supernatant was analyzed using a goat-specific ELISA kit (Jiangsu Meimian Industrial Co., Ltd., Jiangsu, China) with the guidance of manufacturer's specification. The total protein concentration of the supernatant was measured using a BCA protein assay kit (Beyotime Biotechnology, Shanghai, China). The IgG concentration in jejunal tissue was presented as mg/mg protein.

### 2.7 Metabolomics analysis

Metabolites from 50 mg of jejunal tissue were extracted using an extraction solvent (acetonitrile-methanol–water, 2:2:1, containing internal standard), which was then vortexed for 30 s, homogenized at 45 Hz for 4 min, and sonicated for 5 min in the ice-water bath. The homogenates were incubated at −20 °C for 1 h and centrifuged at 12,000 × *g* at 4 °C for 15 min to obtain supernatants. The quality control sample was prepared by mixing an equal aliquot of the supernatants from all of the samples. The mixed supernatants were then analyzed for metabolomics using a hybrid quadrupole-time-of-flight mass spectrometer coupled to a UHPLC system (1290, Agilent Technologies, Santa Clara, CA, USA) and Q Exactive (Orbitrap MS, Thermo, Waltham, MA, USA). A preheated ACQUITY UPLC HSS T3 column (2.1 mm × 100 mm, 1.8 μm internal diameter, Waters, Milford,MA, USA) was used for chromatographic separation in both positive ion (PI) mode and negative ion (NI) mode. The samples were eluted with a mobile phase containing solvent A (0.1% formic acid in water for PI mode and 5 mmol/L ammonium acetate in water for NI mode) and solvent B (acetonitrile with 0.1% formic acid) at a flow rate of 0.5 mL/min, with a sample injection volume of 3 μL. Furthermore, the QE mass spectrometer was used to acquire MS/MS spectra on an information-dependent basis with the acquisition software (Xcalibur 4.0.27, Thermo, Waltham, MA, USA) that continuously evaluates the full scan survey MS data.

Raw data were first converted to the mzML format using ProteoWizard and processed to obtain retention time alignment, peak detection, and peak matching by the XCMS R package (version 3.2). Subsequently, the above data were annotated with the MS2 internal database (BiotreeDB) with a cutoff value of 0.3. Orthogonal partial least squares discriminant analysis (OPLS-DA) was conducted to evaluate the metabolic changes between two groups using SIMCA (version 14.1). The differential metabolites were filtered using the variable importance in projection (VIP) generated in OPLS-DA, *P*-value, and fold change (FC) obtained in statistical analysis (VIP >1, *P* < 0.05, and | FC| > 1.2).

### 2.8 RNA isolation, sequencing and transcriptome analysis

Total RNA was isolated from jejunal tissue using a Trizol reagent kit (Invitrogen, Carlsbad, CA, USA) according to the manufacturer's standard protocol. RNA quality was assessed using an Agilent 2100 Bioanalyzer (Agilent Technologies, Santa Clara, CA, USA) and 1% agarose gel electrophoresis. The eukaryotic mRNA was enriched by Oliogo (dT) beads and broken down into short fragments followed by reverse transcription. The cDNA fragments were purified with a QIAquick Gel Extraction Kit (Qiagen, Venlo, The Netherlands) and sequenced on the Illumina HiSeq2500 platform (Illumina, San Diego, CA, USA). The raw reads of RNA sequencing are available at the NCBI Sequence Read Archive under the accession number PRJNA799403.

Raw reads were subjected to quality control to remove adapters and low-quality bases by Trimmomatic (version 0.39) (Bolger et al., 2014). Bowtie2 (v2.2.8) tool was used to align reads to the ribosome RNA (rRNA) database and the rRNA mapped reads were removed to obtain clean mRNA reads (Langmead and Salzberg, 2012). Paired-end clean reads were mapped to the goat reference genome (ARS1) using HISAT (version 2.2.4) with default parameters (Kim et al., 2015). The mapped reads of each sample were assembled using StringTie (version 1.3.1) in which the fragment per kilobase of transcript per million mapped reads (FPKM) value was calculated to quantify gene expression (Pertea et al., 2015). Expressed genes (FPKM >1 in at least 50% of the samples in one group) were used to conduct differentially expressed gene (DEGs) analysis using DESeq2 software (Love et al., 2014) and other analyses. Genes with an adjusted *P* < 0.05 and |FC| > 1.2 were considered differentially expressed. The Kyoto Encyclopedia of Genes and Genomes (KEGG) pathway enrichment for DEGs was performed on KOBAS software (version 3.0; Bu et al., 2021), and pathways on level 3 underwent variance analysis in which adjusted *P* < 0.05 was considered as a significant difference.

### 2.9 Weighted gene co-expression network analysis (WGCNA)

WGCNA was conducted to explore the link between phenotypic values related to IgG absorption and jejunal transcriptome, aiming to reveal the molecular mechanism of IgG absorption in neonatal goats. The genes (12,867, average FPKM >2 in all samples) in jejunal tissue samples collected from all neonatal goats were used in WGCNA (R package; Langfelder and Horvath, 2008). A gene co-expression network was constructed based on Pearson's correlation using the soft thresholding power. Module detection (blockwise modules in WGCNA) functions were performed with the following parameters: minBlockSize of 100, maxModuleSize of 30 and reassign threshold of 0.1. The relationships between the resulting gene modules and the phenotypic values were evaluated using Pearson's correlation analysis. Modules with |*r*| > 0.70 and adjusted *P* < 0.01 were defined as significant and used for downstream functional analysis.

### 2.10 MicroRNA (miRNA) sequencing, differential analysis and functional analysis

The RNA molecules in a size range of 18 to 30 nt were enriched by polyacrylamide gel electrophoresis from the above extracted total RNA. The enriched RNA was added to 3′ and 5' adapters and then used for microRNA-Seq library construction by a TruSeq Small RNA Sample Preparation Kit (Illumina, CA, USA). Small RNA sequencing was conducted using an Illumina HiSeq 2500 platform (Illumina, CA, USA). The raw reads of miRNA sequencing are available at the NCBI Sequence Read Archive under the accession number PRJNA799234. The adaptors and low-quality bases were removed to obtain tags that were further aligned with small RNAs in the GenBank database (Release 209.0) and Rfam database (version 11.0) to identify (Griffiths-Jones et al., 2003) and remove rRNA, scRNA, snoRNA, snRNA, and tRNA. The tags were aligned with the reference genome (ARS1), and mapped exons, introns, and repeat sequences were removed to obtain clean tags. Clean tags were searched against the miRBase database (Release 22) to identify known miRNAs (Griffiths-Jones, 2006). The read number of detected miRNAs was normalized as transcripts per million (TPM), and the miRNAs with average TPM > 1 in at least one group were defined as expressed miRNAs. Differentially expressed (DE) miRNAs were identified using the edgeR package (Robinson et al., 2010) with the threshold of |FC| > 2 and adjusted *P* < 0.05. The candidate target genes for DE miRNAs were predicted using Miranda (version 3.3a) and TargetScan (version 7.0) software (Lewis et al., 2005, Turner, 1985). The union set of target gene lists generated from the above software was used to perform functional enrichment in KOBAS software.

### 2.11 Quantitative real-time PCR (qRT-PCR) validation

Total RNA from jejunal tissue was extracted using AG RNAex Pro Reagent (AG21101, Accurate Biology, Changsha, China) according to the manufacturer's instructions. After detection of concentration and quality by NanoDrop 2000 (Thermo Scientific, MA, USA), the extracted RNA was prepared for reverse transcription using a commercial Evo M-MLV Reverse Transcription Kit (AG11705, Accurate Biology, Changsha, China) and the cDNA was stored at −20 °C. The reverse transcription of miRNA was performed using total RNA with a miRNA 1st Strand cDNA Synthesis Kit (AG11717, Accurate Biology, Changsha, China).

qRT-PCR was conducted on a fluorescence LightCycler 480 II platform (Roche, Basel, Switzerland) using SYBR Green Premix Pro Taq HS qPCR Kit (Accurate Biology, Changsha, China). The reaction system and PCR program were prepared according to the manufacturer's recommendation. The quantification cycle of each mRNA was normalized by the internal control gene (β-actin) using the 2^−ΔΔCt^ method. In the reaction system of miRNA, the forward primers were synthesized by adding poly(A) and the reverse primer was provided by a reverse transcription kit. The relative expression of each miRNA was normalized by U6 snRNA and calculated using the 2^−ΔΔCt^ method. The paired primers of mRNAs and forward primers of miRNAs are deposited in Table S1.

### 2.12 Statistical analysis

Data on growth performance, serum IgG level and AEA of jejunal IgG, jejunal IgG concentration, and the expression of mRNAs and miRNAs were subjected to a student's *t*-test using SPSS 24.0 software. The mathematical model for the *t*-test is:

*Yij* = *μ* + *Ti*+ *εij*,

where *Yij* is the observed value of the dependent variable for the *j*-th observation in the *i*-th group; *μ*, the overall mean; *Ti*, the effect of the *i*-th group; and *εij*, is the random error. Data were presented as mean and standard error of the mean (SEM), and the threshold of significance was set at *P* < 0.05.

## 3 Results

### 3.1 Effects of delaying colostrum feeding on growth performance, diarrhea rate and IgG absorption in neonatal goat kids

As shown in Table 1, the birth weight (*P* = 0.223), final weight (*P* = 0.341), and weight gain (*P* = 0.089) of neonatal goat kids in the NCF and DCF groups did not exhibit any significant differences. During the experimental period, none of the goat kids presented diarrhea. To evaluate the IgG absorption of neonatal goats when colostrum feeding was delayed, the serum IgG level and AEA were detected. Compared to the NCF group, serum IgG level (*P* < 0.001), AEA (*P* < 0.001) and jejunal IgG level (*P* = 0.003) were lower in DCF goats (Table 1).

**Table 1:** Effects of delaying colostrum feeding on growth performance, diarrhea rate and immunoglobulin G (IgG) absorption in neonatal goat kids.

Item	Group^1^		SEM	*P*-value^2^
	NCF	DCF		
Birth weight, kg	1.96	2.15	0.077	0.223
Final weight, kg	1.89	2.03	0.071	0.341
Weight gain, kg	−0.067	−0.121	0.0158	0.089
Diarrhea rate, %	0	0		
Serum IgG concentration, mg/mL	5.38	2.96	0.346	<0.001
AEA, %	52.1	28.7	3.34	<0.001
Jejunal IgG level, mg/mg protein	2.47	0.55	0.332	0.003

AEA = the maximum apparent efficiency of absorption of IgG; SEM = standard error of the mean.

1 NCF = normal colostrum feeding; DCF = delayed colostrum feeding.

2 *P* < 0.05 represents statistical differences, *n* = 8.

### 3.2 Metabolomic analysis in the jejunal epithelium of neonatal goat kids

To obtain a comprehensive metabolomic profile of jejunal tissue, liquid chromatography-mass spectrometry was used to separate and identify metabolites. A total of 216 and 119 reliable metabolites, mainly containing amino acids and their derivatives, fatty acids, organic acids and others, were identified in the jejunal tissue after quality control on PI (Table S2) and NI modes (Table S3), respectively. The score plots of OPLS-DA in PI and NI modes displayed a distinct separation between the NCF and DCF groups (Figs. S1A and B), indicating that colostrum feeding time altered the jejunal metabolites. In addition, to assess the effects of delayed colostrum feeding on jejunal metabolites, the VIP values obtained from PLS-DA analysis and statistical analysis were used to filter differential metabolites. In total, 36 and 19 metabolites (VIP > 1.0, *P* < 0.05, FC > 1.2 or < 0.83) were observed in the comparison of the NCF and DCF groups based on PI and NI mode, respectively (Figs. S1C and D). We further combined the differential metabolites in PI and NI modes, and found that 11 metabolites were related to amino acid metabolism and 8 metabolites were associated with fatty acid metabolism (Fig. 1B). Subsequently, we used the above metabolites to perform functional enrichment analysis based on the KEGG database. The results showed that these differential metabolites were significantly enriched (*P* < 0.05) in phenylalanine, tyrosine and tryptophan biosynthesis, phenylalanine metabolism, and glycerophospholipid metabolism (Fig. 1C).

### 3.3 Transcriptomic analysis in the jejunal epithelium of neonatal goat kids

**Fig. 2:**
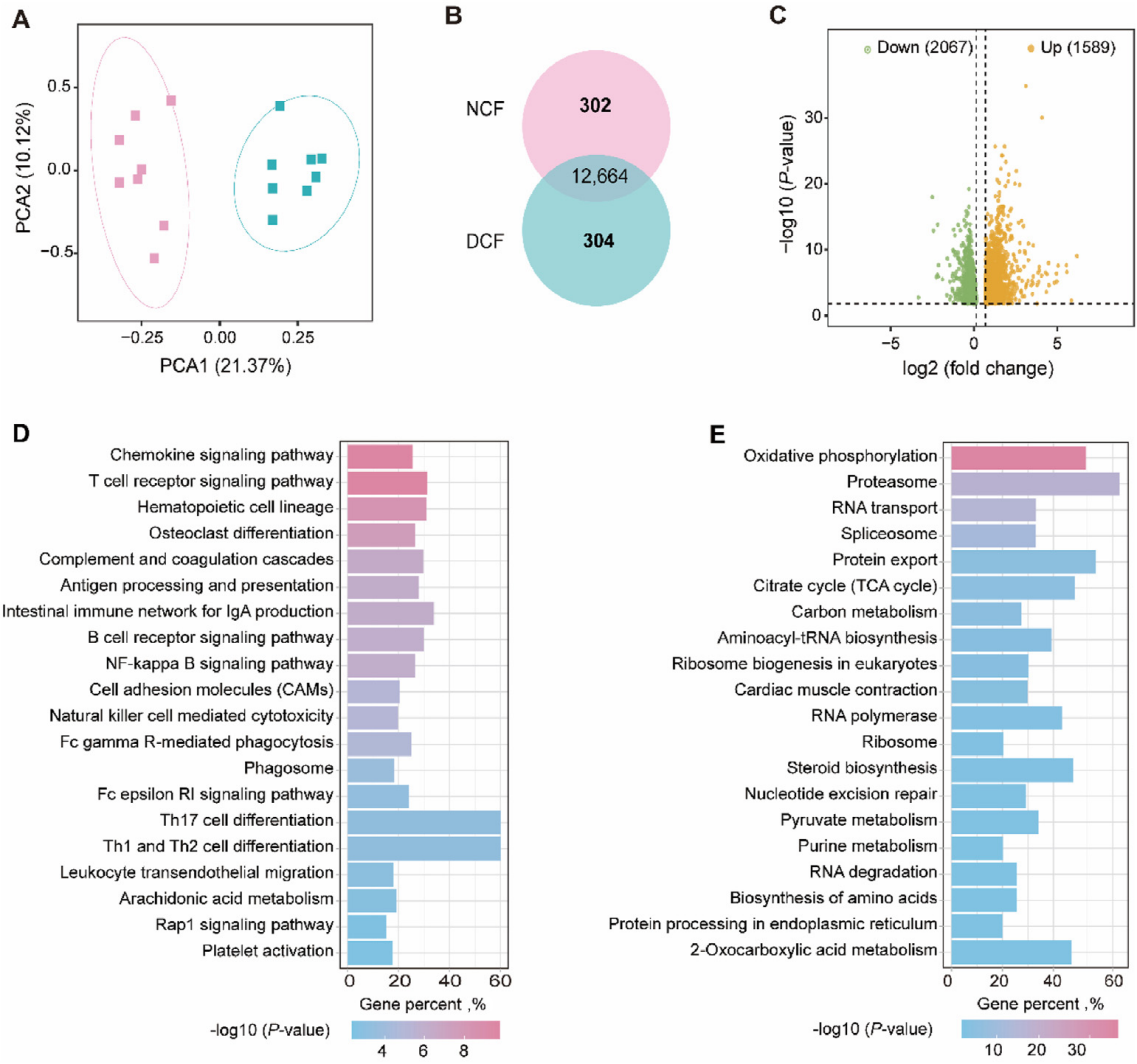
Principal component analysis, differentially expressed gene (DEG) analysis and the Kyoto Encyclopedia of Genes and Genomes (KEGG) enrichment analysis of transcriptomic profiles of jejunal tissues in neonatal goats. (A) Principal component analysis of genes expressed in the jejunum of neonatal goat kids. (B) Venn diagram of co-expressed and uniquely expressed genes between NCF and DCF group. All genes of this analysis were expressed genes with FPKM >1 in at least 50% samples of one group. (C) The volcano plot of DEGs (|fold change| > 1.2 and adjusted *P* < 0.05). KEGG enrichment analysis of upregulated (D) and downregulated (E) DEGs. Color bar represents -log10 (*P-*value) and the rank of pathway from top to bottom is according to adjusted *P* values from low to high. NCF = normal colostrum feeding; DCF = delayed colostrum feeding; DEGs = differentially expressed genes; PCA = principal component analysis.

The data of RNA-Seq revealed that a total of 13,277 genes were expressed (FPKM > 1 in 50% of samples in at least one group) in the jejunal tissue of neonatal goats. Principal component analysis based on the expressed genes showed that the jejunal transcriptomic profiles were distinctly separate from each other (Fig. 2A). Among the above-mentioned expressed genes, a total of 12,664 genes were co-expressed in the two groups, while 302 and 304 genes were uniquely expressed in the NCF and DCF groups, respectively (Fig. 2B). Unique genes expressed in the NCF group were mainly involved in the growth-related processes (Fig. S2A), while genes uniquely expressed in the DCF group were enriched in the immune pathways (Fig. S2B). A total of 3656 DEGs (FC| ≥ 1.2 and adjusted *P* < 0.05) were identified between NCF and DCF groups, in which 1589 and 2067 genes were up-and down-regulated, respectively (Fig. 2C). KEGG pathway enrichment analysis showed that upregulated DEGs were mainly enriched in immune-related processes (Fig. 2D and Table S4), while downregulated DEGs were involved in metabolism-related pathway (Fig. 2E and Table S5).

### 3.4 Potential genes involved in the endocytosis pathway that contribute to the molecular mechanism regulation of IgG absorption in the jejunum

As demonstrated in a previous study, colostral IgG absorption can be accomplished through endocytosis from the lumen of the small intestine to the basolateral side of the enterocytes (Pyzik et al., 2015). We found that 1 (*TGFB2*) and 6 genes (*CCR5*, *HSPA6*, *LOC102183314*, *LOC102174322* and *LOC 102181347*) involved in endocytosis were uniquely expressed in the NCF and DCF groups, respectively (Fig. S2C). Specifically, *TGFB2* is a key gene in the transforming growth factor-β (TGF-β) signaling pathway, while other genes expressed in the DCF group were mainly related to cytokine–cytokine receptor interaction and phagosome, which had no relationship with IgG absorption. Among all DEGs, upregulated genes were significantly enriched in endocytosis (*P* = 0.032). In contrast, the downregulated genes failed to be enriched in this pathway (*P* > 0.05). We summarized the DEGs enriched in endocytosis and found that 28 upregulated and 35 downregulated DEGs were related to that pathway (Fig. S2D). Therefore, clarifying the absorptive pathway is challenging due to the vast number of candidate genes.

### 3.5 WGCNA filters the key genes contributing to IgG absorption in the jejunum

**Fig. 3:**
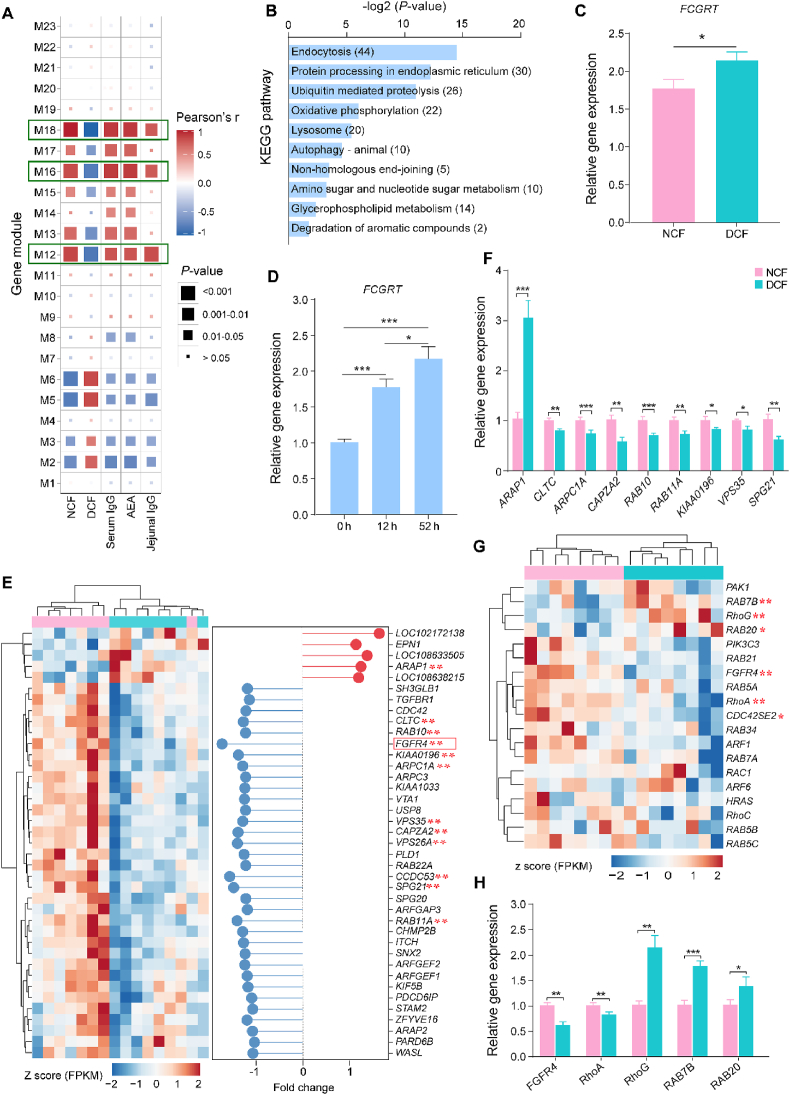
Gene module correlated with the phenotypic values (treatment, serum IgG, AEA, jejunal IgG) in the neonatal goats using a weighted gene co-expression network analysis, the Kyoto Encyclopedia of Genes and Genomes (KEGG) enrichment analysis and the expression of filtrating genes contributed to IgG absorption. (A) Relationship between gene modules (gene modules are defined as M1 to M23) and phenotypic values. Gene modules were obtained using a weighted gene co-expression network analysis. (B) Top 10 KEGG pathways of genes expressed in the M12 modules. Numerical values in parenthesis represents gene number of the pathways. The rank of pathway from top to bottom is according to values of adjusted *P* from low to high. (C) Relative expression of the Fc fragment of the IgG receptor (*FCGRT*) in the jejunal tissue of neonatal goat kids by quantitative real-time PCR (qRT-PCR). ∗ represents *P* < 0.05. (D) Relative expression shifts of *FCGRT* in the jejunum at different developmental time points by qRT-PCR. ∗ represents *P* < 0.05 and ∗∗∗ represents *P* < 0.001. (E) Heatmap of the expression of genes involved in endocytosis in the M12 module. ∗∗ represents adjusted *P* < 0.01. Gene with red frame represents the initial gene of micropinocytosis. (F) Validation of DEGs involved in clathrin-mediated endocytosis by qRT-PCR. ∗, ∗∗, ∗∗∗ represents *P* < 0.05, *P* < 0.01 and *P* < 0.001, respectively. (G) Heatmap of the expression of genes involved in macropinocytosis in the M12 module. ∗ represents adjusted *P* < 0.05 and ∗∗ represents adjusted *P* < 0.01. (H) Validation of DEGs involved in macropinocytosis by qRT-PCR. ∗, ∗∗, ∗∗∗ represents *P* < 0.05, *P* < 0.01 and *P* < 0.001, respectively. NCF = normal colostrum feeding; DCF = delayed colostrum feeding; IgG = immunoglobulin G; AEA = the maximum apparent efficiency of absorption of IgG; DEGs = differentially expressed genes.

To further filter the key genes associated with IgG absorption, we used phenotypic values (experimental treatment, serum IgG level, AEA and jejunal IgG level) and transcriptomic data to perform WGCNA. A total of 12,867 genes (FPKM > 2) in jejunal tissues, which were used to conduct WGCNA were clustered into 23 gene modules, defined as M1 to M23 modules (Fig. S3A). Pearson's correlation showed that the expressions of genes in the M12 module (1621 genes, 12.60% of total genes), M16 module (1480 genes, 11.50% of total genes) and M18 module (1392 genes, 10.82% of total genes) were significantly positively correlated (|*r*| > 0.70 and adjusted *P* < 0.01) with phenotypic traits (Fig. 3A and Fig. S3B). The expression of genes in the above 3 modules was higher in the NCF group than those in the DCF group of jejunal tissue (Fig. S3C). Jejunal genes co-expressed in the M16 module were related to ribosome biogenesis, RNA transport, spliceosome, cell cycle, protein export and other molecular biological functions (Fig. S4A). Genes co-expressed in the M18 module were enriched in oxidative phosphorylation, carbon metabolism, citrate cycle, biosynthesis of amino acids, glycolysis/gluconeogenesis and other metabolic pathways (Fig. S4B).

The M12 module, clustered genes that were related to endocytosis and had a positive correlation with serum IgG, AEA, and jejunal IgG level, was selected for further analysis to explore the potential molecular mechanism involved in the process of IgG absorption. Among 553 genes in the M12 module annotated against the KEGG database, the largest proportion (44 genes, 7.96%) were related to endocytosis. Furthermore, 30 genes (5.42%) were related to protein processing in the endoplasmic reticulum, 26 genes (4.70%) were associated with ubiquitin mediated proteolysis, 22 genes (3.98%) were involved in oxidative phosphorylation and 20 genes (3.62%) were enriched in the lysosome (Fig. 3B). Using the ClueGO (version 2.5.8) application in Cytoscape (version 3.9.0), an interaction was found among the 5 pathways mentioned above, which were linked by 7 joint genes (Fig. S4C). Among those genes, *ITCH* was the joint point between endocytosis and ubiquitin-mediated proteolysis, *CLTC* linked endocytosis and lysosome, *UBE2G1*, *UBE2J1*, and *UBE2D3* joined ubiquitin-mediated proteolysis and protein processing in endoplasmic reticulum, *ATP6V0D1* and *ATP6AP1* were the joint points between lysosome and oxidative phosphorylation.

As mentioned, FcRn is a vital receptor mediating bidirectional IgG transportation in the epithelium (Claypool et al., 2004). To assess whether the expression of FcRn was affected by delayed colostrum feeding, we determined the relative expression of the Fc fragment of the IgG receptor (*FCGRT*) in the jejunum by qRT-PCR and the results showed that neonatal goats in the DCF group had higher *FCGRT* expression than those in the NCF group (Fig. 3C). We further verified whether the changes of *FCGRT* were induced solely by time after birth through determining the jejunal *FCGRT* expression in neonatal goats at different time points after birth (0, 12 and 52 h, all goats were fed colostrum at 5% BW within the first 4 h after birth except for goats slaughtered at 0 h). We found that the expression of *FCGRT* significantly increased with extended developmental time (Fig. 3D). The results indicated that the processes of endocytosis rather than FcRn might be the main factor contributing to the regulation of IgG absorption in neonatal goat kids. Subsequently, we aggregated the expression levels and FC of genes in the M12 module that were enriched in endocytosis and found 12 DEGs included in that pathway (Fig. 3E). Of those 12 DEGs, 11 DEGs were related to clathrin-mediated endocytosis (CME). We further quantified the relative mRNA abundance by qRT-PCR, and the results showed drastic shifts in expression of *ARAP1*, *CLTC*, *ARPC1A*, *CAPZA2*, *RAB10*, *KIAA0196*, *RAB11A*, *VPS35* and *SPG21*, which are involved in clathrin synthesis, actin-related clathrin-coated vesicle formation and sorting and recycling endosome (Fig. 3F). Furthermore, *FGFR4* is a key gene regulating the synthesis of receptor tyrosine kinases that initiate the macropinocytosis process (Palm, 2019). Macropinocytosis, an important process of macromolecular nutrient uptake, has a potential role in IgG absorption. Thus, we listed functional genes in the M12 module involved in macropinocytosis according to the KEGG database and the results showed that 6 DEGs were involved in that pathway, participating in the formation and maturation of macropinosomes (Fig. 3G). The results of qRT-PCR showed that the expression of *FGFR4*, *RhoA*, *RhoG*, *RAB7B* and *RAB20* was affected by delayed colostrum feeding (Fig. 3H).

### 3.6 miRNA regulates the DEGs of CME and macropinocytosis

**Fig. 4:**
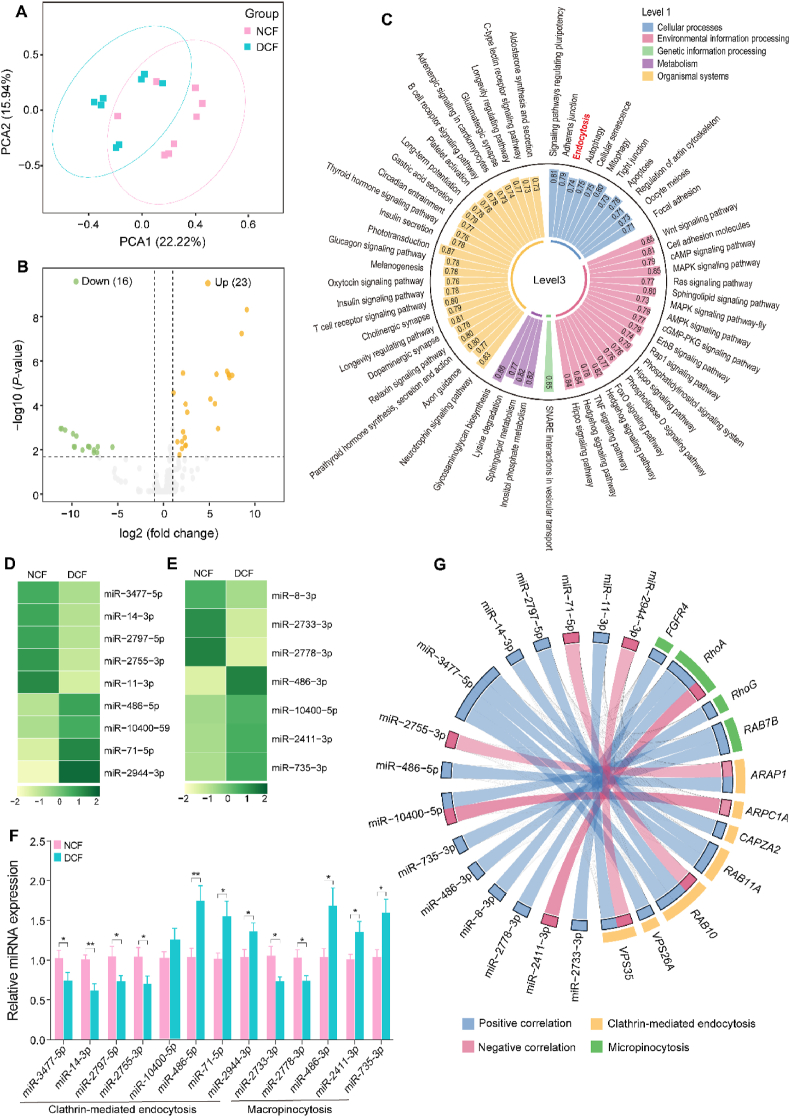
MicroRNA (miRNA) sequencing profiles and regulatory mechanisms of miRNA in clathrin-mediated endocytosis and macropinocytosis. (A) Principal component analysis of known miRNAs. (B) Volcano plot of differentially expressed miRNAs (|fold change| > 2 and adjusted *P* < 0.05) between the NCF and DCF group. (C) Top 60 KEGG pathways of target genes on level 1 and level 3. Color represents a different class on level 1. Number labeled on each column represents gene percentage (the ration of gene number enriched on each pathway to background gene number on the same pathway). (D, E) List of miRNAs involved in clathrin-mediated endocytosis (D) and macropinocytosis (E). **(**F**)** Validation of DE miRNAs involved in clathrin-mediated endocytosis and macropinocytosis by qRT-PCR. ∗, ∗∗ represents *P* < 0.05, *P* < 0.01, respectively. (G) Relationship between miRNAs (miRNA expression) and target genes (mRNA expression). miRNA-mRNA pairs in pink color indicate miRNA may negatively regulate mRNA expression. KEGG = the Kyoto Encyclopedia of Genes and Genomes; PCA = principal component analysis; NCF = normal colostrum feeding; DCF = delayed colostrum feeding; DE = differentially expressed; qRT-PCR = quantitative real-time PCR.

The sequencing reads generated from 16 miRNA libraries were prepared from jejunal tissues. A total of 2386 miRNAs were identified as known miRNAs, and 754 expressed miRNAs (with TPM > 1 in at least 50% of the samples in one group) were obtained after filtering out low-expressed miRNAs. The principal component analysis (PCA) for expressed miRNAs in the NCF and DCF groups is shown in Fig. 4A. Based on all expressed miRNAs, there were 27 up-regulated and 16 down-regulated miRNAs identified with absolute FC > 2 and adjusted *P* < 0.05 (Fig. 4B, Table S6). The use of TargetScan and miRbase revealed that these DE miRNAs might regulate 79,216 mRNAs in total. Afterward, the results of KEGG enrichment analysis showed that these miRNAs were significantly enriched (*P* < 0.05) in endocytosis (Fig. 4C and Table S7), indicating a regulation by miRNAs in the endocytic process. To further elucidate the potential regulatory mechanisms of miRNAs in endocytosis, we summarized all DE miRNAs that regulate mRNAs involved in CME and macropinocytosis. Specifically, a total of 9 DE miRNAs and 7 targeted genes were related to CME (Fig. 4D and G), and 7 miRNAs had 4 predicted DEGs associated with macropinocytosis (Fig. 4E and G). We further quantified the relative expression abundance of miRNAs by qRT-PCR (Fig. 4F), and the results showed that the expressions of 8 DE miRNAs related to CME and 5 DE miRNAs related to macropinocytosis were significantly affected by delayed colostrum feeding. Among these miRNA-mRNA gene pairs, only 4 pairs (miR-2755-3p and *ARAP1*, miR-10400-5p and *ARPC1A*, miR-71-5p and *RAB10*, miR-2944-3p and *VPS35*) involved in CME and 1 pair (miR-2411-3p and *RhoA*) associated with macropinocytosis showed interaction (Fig. 4G).

## 4 Discussion

It has been shown that the small intestine of neonatal ruminants can absorb macromolecules and this capacity disappears 24 h after birth due to “gut closure” (Lorenz et al., 2011). In the current study, goat kids received colostrum during the first 4 h (NCF) or at 44 h after birth (DCF), with the experiment designed to evaluate the mechanisms leading to the difference in IgG absorption before and after gut closure, respectively. As expected, the serum IgG levels and AEA of goat kids in the NCF group were higher than those in the DCF group. Meanwhile, the IgG concentration of jejunal tissue was remarkably higher in goat kids from the NCF group than those from the DCF group, suggesting that IgG absorption nearly terminated after gut closure.

In suckling mammals, the absorptive cells of the small intestine are able to uptake luminal macromolecules through active fluid-phase or membrane-bound endocytosis (Fujita et al., 2007). The neonatal Fc receptor (FcRn) transfers IgG from maternal milk to the offspring's circulation across the proximal small intestine by transcytosis (Roopenian and Akilesh, 2007). In detail, FcRn synthesis occurs in the rough endoplasmic reticulum and is then processed in the cis-golgi network and eventually transported to and incorporated into the apical membrane domain (Kumagai et al., 2011). In the process of IgG absorption during the neonatal period, FcRn binding with IgG is the first step, occurring in an acidic pH environment. The FcRn–IgG complex is then internalized into coated vesicles and transported through endosomes in the enterocytes of the small intestine (Lardner, 2001). Finally, the FcRn–IgG complex fuses to the basolateral membrane and then releases IgG into the intercellular space of the absorptive cell. Subsequently, the FcRn-containing endosome recycles back to the apical membrane domain via the process of basolateral transcytosis (Ober et al., 2004). In the current study, the relative expression of *FCGRT*, one of the components of FcRn, was higher when colostrum feeding was delayed, which is not in line with the results of serum IgG and AEA. The increased expression of *FCGRT* in neonatal goat kids at different time points (0, 12, and 52 h) after birth indicated that the relative expression of *FCGRT* was time-dependent, which was consistent with a previous study showing that the relative mRNA expression of FcRn increased with culture days in Caco-2 cells (Sato et al., 2009). Overall, the available evidence shows that FcRn might not be the main factor regulating IgG absorption in neonatal goat kids fed delayed colostrum.

Our study demonstrated transcriptional changes in the process of endocytosis in neonatal goat kids subjected to delayed colostrum using transcriptomic analysis. Endocytosis, one of the basic cellular events that traffics macromolecules to intestinal absorptive cells, has many different pathways including CME, caveola-mediated endocytosis, macropinocytosis, and clathrin/caveola-independent endocytosis (Kumari et al., 2010). The WGCNA analysis using mRNA transcriptome and phenotypic values related to the capacity of IgG absorption showed that genes co-expressed in the M12 module were significantly enriched in endocytosis, especially CME. Generally, CME is a key pathway for vesicular trafficking that transports a wide range of surface receptors and their bound ligands (cargoes), including nutrients, cell adhesion and cell signaling receptors from the cell surface into intracellular membrane compartments (Mettlen et al., 2018). The initiation of the CME process involves clathrin and other coated proteins gathering on the inner leaflet of the plasma membrane from the cytosolic pool, followed by the recruitment of cargo molecules to the coated region of the plasma membrane. In addition, the assembling coated proteins facilitate membrane bending and form a ‘clathrin-coated pit’, scission and actin proteins constrict and cut the neck of the membrane invagination to separate the clathrin-coated vesicle from the plasma membrane. Finally, coat proteins are disassembled and uncoated cargo-filled vesicles are released to fuse with endosomes (Kaksonen and Roux, 2018). In the above processes, clathrin plays a vital role in IgG and FcRn coating. The gene clathrin heavy chain (*CLTC*), which regulates the synthesis of heavy-chain subunits of clathrin, is an important protein-coding gene that controls CME initiation, especially clathrin coating cargos (Kirchhausen et al., 2014). The present study revealed that the expression of *CLTC* was decreased in neonatal goat kids from the DCF group, suggesting that delayed colostrum feeding affected the synthesis of clathrin, which restrained the formation of vesicles to coat IgG and FcRn. In addition to the clathrin coat, the formation of the actin filaments at the endocytic sites is an indispensable step during the formation of clathrin-coated vesicles. Actin-related protein 2/3 complex (ARP2/3) mediates actin filament nucleation around the base of the invagination to facilitate membrane deformation or movement that is regulated by actin-related protein 2/3 complex subunit 1A (ARPC1A) (Sirotkin et al., 2005). Furthermore, ARP2/3 needs to be activated by nucleation-promoting factors (NPFs), and WASH complex, as an NPF, is composed of F-actin-capping protein subunit alpha (CAPZA2) and other WASH complex subunits (WASHC1-5) that generate an actin network on a restricted domain of sorting and recycling endosomes (Derivery et al., 2009). The protein ARAP1 targets multiple signals by activating different GTP-binding proteins to coordinate actin and membrane remodeling (Miura et al., 2002). In the current study, the expressions of *ARPC1A*, *CAPZA2* and *WASHC5* (*KIAA0196*) were reduced in the DCF group, suggesting that delayed colostrum feeding affected the actin-mediated formation of clathrin-coated vesicles, sorting and recycling endosomes, which restricted IgG absorption through CME.

In humans and rodents, the FcRn–IgG complex is internalized into a vesicle, fuses with an endosome, undergo sorting of FcRn–IgG complex, and is eventually released to neonatal circulation at physiological pH (Roopenian and Akilesh, 2007). Vacuolar protein sorting-associated protein 35 (VPS35) is believed to contribute to sorting polymeric immunoglobulin receptors and preventing them from undergoing lysosomal degradation (Vergés et al., 2004). The present study showed that the expression of *VPS35* was lower in neonatal goat kids from the DCF group, which may be a developmental time-dependent factor, as discussed for *FCGRT* above. GTPases of the family of Rab protein control vesicle fusion, and over 60 Rab proteins with different functions in the intracellular transport step have been identified in mammals (Zerial and McBride, 2001). Among them, Rab10 mediates intracellular transport from basolateral sorting endosomes to common endosomes (Babbey et al., 2006). Rab11 has a primary function of transporting cargo from the apical recycling endosomes to the plasma membrane in polarized cells (Brown et al., 2000). The results of RNA-Seq and qRT-PCR showed that gene expressions of *RAB10* and *RAB11A* were lower in neonatal goat kids from the DCF group, indicating that it might create an obstacle for FcRn recycling from basolateral sorting endosomes to the plasma membrane on the cell surface and ultimately reducing the number of FcRn receptor available for the next IgG transport.

Unlike other endocytic pathways, macropinocytosis is a non-selective liquid-phase endocytic pathway for the uptake of extracellular substances. The initiation of this process involves the wrapping of exogenous substances by cell membrane deformation and, subsequently, the formation of endocytic vesicles through fusion with the cell membrane, which is controlled by growth factor signaling (Palm, 2019; Zheng et al., 2021). Tyrosine kinases (RTKs), coupled with their effectors, the small GTPase Ras and Class I phosphatidylinositol 3-kinase (PI3-kinase), orchestrate actin-driven membrane ruffling and macropinosome formation after being activated by growth factors (Swanson, 2008). In mammalian cells, Ras and PI3-kinase can be activated by RTKs, including epidermal growth factor receptor and platelet-derived growth factor receptor, and macropinocytosis is blocked once RTKs or PI3-kinase are inhibited (Araki et al., 1996). In the current study, *FGFR4* encoding a tyrosine kinase had a lower relative expression in neonatal goat kids when subjected to delayed colostrum feeding. Furthermore, *RhoA*, which plays a role in the formation of macropinosomes (Pertz et al., 2006), was inhibited in the DCF group. These results indicated that IgG absorption in the jejunum of neonatal goats through macropinocytosis was restricted mainly by the lower expression of genes involved in membrane ruffling and macropinosome formation when colostrum feeding was delayed. After that, macropinosomes undergo maturation and fuse with lysosomes to release trafficking cargos, and the macropinosome membrane is recycled to the cell surface (Song et al.*,* 2021). In the above processes, *RAB7B* and *RAB20* are markers of mature or late macropinosomes (Egami and Araki, 2012; Kerr et al., 2006), and the expressions of these 2 genes were higher in the DCF group. The findings that mature or late macropinosomes had limitations in returning to the cell surface through recycled vesicles, leads to the accumulation of large numbers in enterocytes and reduces the capacity to participate in a new macropinosome formation.

Our study also identified several novel miRNA markers that may regulate the process of endocytosis in neonatal goat kids subjected to delayed colostrum feeding. The present study emphasized the potential regulation of miRNA on the above-discussed genes using miRNA sequencing. A total of 4 miRNAs were negatively correlated with target genes involved in CME and macropinocytosis. Specifically, *ARPC1A*, *RAB10*, and *VPS35* were targeted by miR-10400-5p, miR-71-5p, and miR-2944-3p, respectively, which mediated actin-related clathrin-coated vesicle formation, FcRn–IgG sorting and FcRn recycling in CME. For macropinocytosis, only miR-2411-3p, which regulates *RhoA*, affected macropinosome formation. These predicted novel miRNA markers that may affect IgG absorption in neonatal goat kids need to be further verified and investigated.

## 5 Conclusion

**Fig. 5:**
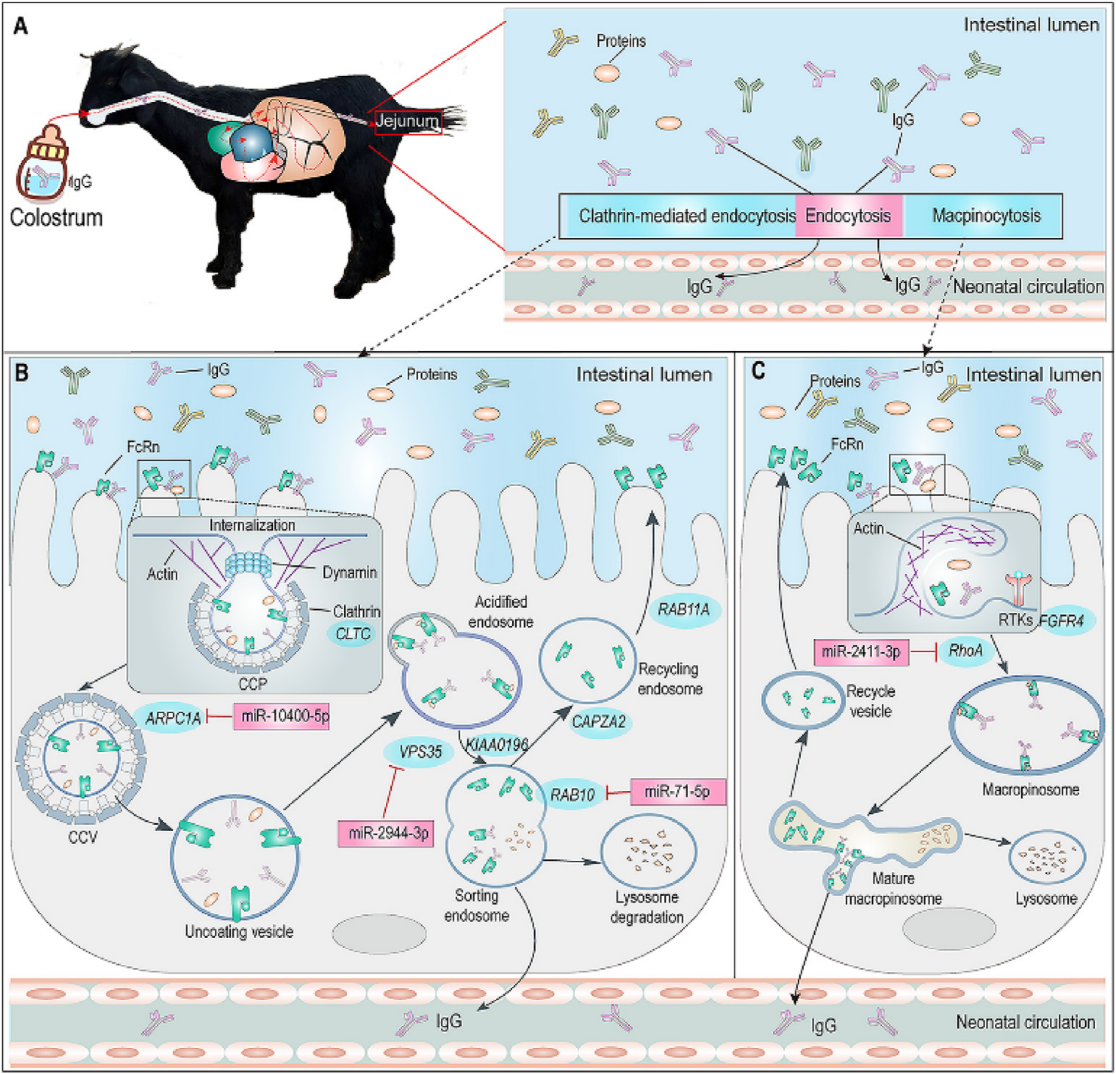
Proposed regulatory mechanisms of IgG absorption from taken colostrum in feed to circulation when neonatal goat kids were subjected to a delayed colostrum feeding. (A–C) IgG absorption is mainly through CME in which functional genes regulate the clathrin synthesis (*CLTC*), the clathrin-coated vesicle (*ARPC1A*), sorting and recycling endosome (*CAPZA2*, *KIAA0196*, *RAB10*, *RAB11A* and *VPS35*) and macropinocytosis pathway, where functional genes regulate macropinosomes formation (*FGFR4* and *RhoA*). miRNAs may negatively regulate mRNAs involved the formation of clathrin-coated vesicles (miR-10400-5p), FcRn–IgG sorting (miR-2944-3p and miR-71-5p) in CME, and macropinosomes formation (miR-2411-3p) in micropinocytosis pathway. Only verified genes tested by qRT-PCR were proposed in the mode. IgG = immunoglobulin G; CME = clathrin-mediated endocytosis; CCP = clathrin coated pit; CCV = clathrin coated vesicle; qRT-PCR = quantitative real-time PCR.

Neonatal goat kids without maternal colostrum uptake after birth were used as animal models to investigate the potential molecular mechanisms regulating IgG absorption. Current results suggest that the endocytosis pathways including CME and macropinocytosis, rather than FcRn, play dominant roles in regulating IgG absorption in the jejunum for neonatal goat kids. As summarized in Fig. 5, the DEGs enriched in CME regulated the clathrin synthesis (*CLTC*), actin-related clathrin-coated vesicle formation (*ARPC1A*) and sorting and recycling endosome (*CAPZA2*, *KIAA0196*, *RAB10*, *RAB11A* and *VPS35*). In addition, the DEGs enriched in macropinocytosis regulated macropinosome formation (*FGFR4* and *RhoA*). Moreover, the miRNAs (miR-10400-5p, miR-2944-3p, miR-71-5p and miR-2411-3p) were predicted to negatively regulate mRNA involved in the formation of clathrin-coated vesicles (*ARPC1A*), FcRn–IgG sorting (*RAB10* and *VPS35*), and macropinosome formation (*RhoA*). Our study provides novel markers and new insights into the molecular mechanism that reduces IgG absorption after gut closure in neonatal ruminants. These findings may provide fundamental knowledge related to the molecular mechanisms of IgG absorption that can support further study in other mammals.

## Credit Author Statement

**Chao Yang:** Writing – original draft, Software, Methodology, Formal analysis, Data curation, Conceptualization. **Yan Cheng:** Formal analysis, Data curation. **Tianxi Zhang:** Validation, Software. **Kefyalew Gebeyew:** Writing – review & editing, Software. **Amanda Fischer-Tlustos:** Writing – review & editing. **Leluo Guan:** Writing – review & editing. **Michael Steele:** Writing – review & editing. **Zhiliang Tan:** Project administration, Funding acquisition. **Zhixiong He:** Writing – review & editing, Project administration, Methodology, Funding acquisition.

## Declaration of competing interest

We declare that we have no financial and personal relationships with other people or organizations that can inappropriately influence our work, and there is no professional or other personal interest of any nature or kind in any product, service and/or company that could be construed as influencing the content of this paper.
