# Supplementary material for: Multi-omics analysis provides new insights into the molecular mechanisms underlying colostral immunoglobulin G absorption in the gut of neonatal goat kids
Source: Anim Nutr. 2025 Jan 25;21:166–78. doi: 10.1016/j.aninu.2024.11.022 (PMC12143632; doi:10.1016/j.aninu.2024.11.022)
Supplement: Multimedia component 1 [file mmc1.docx]

Supplementary Tables

Table of gene symbol and their full name.

| Gene symbol | Full name |
| --- | --- |
| *ARAP1* | ArfGAP with rhoGAP domain, ankyrin repeat and PH domain 1 |
| *ARAP2* | ArfGAP with rhoGAP domain, ankyrin repeat and PH domain 2 |
| *ARF1* | ADP ribosylation factor 1 |
| *ARF6* | ADP ribosylation factor 6 |
| *ARFGAP1* | ADP ribosylation factor GTPase activating protein 1 |
| *ARFGAP2* | ADP ribosylation factor GTPase activating protein 2 |
| *ARFGAP3* | ADP ribosylation factor GTPase activating protein 3 |
| *ARPC1A* | Actin related protein 2/3 complex subunit 1A |
| *ARPC3* | Actin related protein 2/3 complex subunit 3 |
| *ATP6V0D1* | ATPase H^+^ transporting V0 subunit d1 |
| *ATP6AP1* | ATPase H^+^ transporting accessory protein 1 |
| *CCDC53* | Coiled-coil domain containing 53 |
| *CCR5* | C-C motif chemokine receptor 5 |
| *CDC42* | CDC42 effector protein 2 |
| *CDC42SE2* | CDC42 small effector 2 |
| *CHMP2B* | Charged multivesicular body protein 2B |
| *CLTC* | Clathrin heavy chain |
| *CAPZA2* | Capping actin protein of muscle z-line subunit alpha 2 |
| *EPN1* | Epsin 1 |
| *FCGRT* | Fc gamma receptor and transporter |
| *FGFR4* | Fibroblast growth factor receptor 4 |
| *HRAS* | HRas proto-oncogene, GTPase |
| *HSPA6* | Heat shock protein family A member 6 |
| *ITCH* | Itchy E3 ubiquitin protein ligase |
| *KIAA0196* | KIAA0196 ortholog |
| *KIAA1033* | KIAA1033 ortholog |
| *KIF5B* | Kinesin family member 5B |
| *LOC102172138* | BOLA class I histocompatibility antigen, alpha chain BL3-7 |
| *LOC102174322* | Folate receptor alpha |
| *LOC102183314* | BOLA class I histocompatibility antigen, alpha chain BL3-7-like |
| *LOC102181347* | BOLA class I histocompatibility antigen, alpha chain BL3-7-like |
| *LOC108633505* | BOLA class I histocompatibility antigen, alpha chain BL3-7-like |
| *LOC108638215* | Translation initiation factor IF-2-like |
| *PAK1* | p21 (RAC1) activated kinase 1 |
| *PARD6B* | Par-6 family cell polarity regulator beta |
| *PDCD6IP* | Programmed cell death 6 interacting protein |
| *PIK3C3* | Phosphatidylinositol 3-kinase catalytic subunit type 3 |
| *PLD1* | Phospholipase D1 |
| *RAB5A* | Ras-related protein Rab-5 alpha |
| *RAB5B* | Ras-related protein Rab-5 beta |
| *RAB5C* | Ras-related protein Rab-5c |
| *RAB7A* | Ras-related protein Rab-7 alpha |
| *RAB7B* | Ras-related protein Rab-7 beta |
| *RAB10* | Ras-related protein Rab-10 |
| *RAB11A* | Ras-related protein Rab-11 alpha |
| *RAB20* | Ras-related protein Rab-20 |
| *RAB21* | Ras-related protein Rab-21 |
| *RAB22A* | Ras-related protein Rab-22 alpha |
| *RAB34* | Ras-related protein Rab-34 |
| *RAC1* | Ras-related C3 botulinum toxin substrate 1 (rho family, small GTP binding protein Rac1) |
| *RhoA* | Ras homolog family member A |
| *RhoC* | Ras homolog family member C |
| *RhoG* | Ras homolog family member G |
| *SH3GLB1* | SH3 domain containing GRB2 like endophilin B1 |
| *SNX2* | Sorting nexin family member 21 |
| *SPG20* | Spastic paraplegia 20 (autosomal recessive, Mast syndrome) |
| *SPG21* | Spastic paraplegia 21 (autosomal recessive, Mast syndrome) |
| *STAM2* | Signal transducing adaptor molecule 2 |
| *TGFB2* | Transforming growth factor-beta 2 |
| *TGFBR1* | Transforming growth factor beta receptor 1 |
| *UBE2G1* | Ubiquitin conjugating enzyme E2 G1 |
| *UBE2J1* | Ubiquitin conjugating enzyme E2 J1 |
| *UBE2D3* | Ubiquitin conjugating enzyme E2 D3 |
| *USP8* | Ubiquitin specific peptidase 8 |
| *VPS26A* | VPS26, retromer complex component A |
| *VPS35* | VPS35, retromer complex component |
| *VTA1* | Vesicle trafficking 1 |
| *WASL* | WAS/WASL interacting protein family member 1 |
| *ZFVYE16* | Zinc finger FYVE-type containing 16 |

Table S1 Gene symbol and primer sequences for the mRNAs and miRNAs used for quantitative real-time PCR (qRT-PCR) verification.

| Gene symbol | Forward primer (5’-3’) | Reverse primer (5’-3’) |
| --- | --- | --- |
| *FCGRT* | CGGTCTGTTTCTCGTCC | CGCTCGCAGGTTATTG |
| *ARAP1* | CGAAGCCAAATACCG | CGAGAAGCAGTTGACCC |
| *CLTC* | ACTTGCTCAGCGTTTG | ACAGGCTATCTTTCTTGC |
| *ARPC1A* | GGCATCCCAACAACG | CCAAACTCGGACATCAG |
| *CAPZA2* | GCCAAGTTCATCATTCA | TTTCCATTTCCCAAGTC |
| *RAB10* | TTTTCGGATGATGCC | TAGGAGGTTGTGATGGT |
| *RAB11A* | CATCTCAGGGCAGTTC | TTCAGTGGTTGGTGGA |
| *KIAA0196* | ACTCTGCCTGTGACCC | GCCCTCCTTCTTGTAATG |
| *VPS35* | ACTACCTGGAAGTTTACC | AAGCCTTGGGATGATGT |
| *SPG21* | TTATTGGGACCACCTTG | GGACTCTGGGAGATTTGT |
| *FGFR4* | TACAAGGAGGGCAGTCG | GGAGTCGTCCACAACCA |
| *RhoA* | TTCCCAGAGGTGTATGT | CTATCAGGGCTGTCAAT |
| *RhoG* | GGTGGTGGGTGATGGA | CGTTGGTCTGAGGGTAG |
| *RAB7B* | CTGACCTGGAGTCCTTCG | CTCGGGCTACCTCTTGC |
| *RAB20* | GGACGAGAAGGATGTGCC | ATCTGGTCTGTGCGGGTAG |
| *GAPDH* | TTCCACGGCACAGTCAAG | TACTCAGCACCAGCATCACC |
| β-Actin | ACCACTGGCATTGTCATGGACTCT | TCCTTGATGTCACGGACGATTTCC |
| miR-3477-5p | CGCGTAATCTCATTTGGTAACTG | - |
| miR-14-3p | CGCGTCAGTCTTTTTCTCTCTC | - |
| miR-2797-5p | GCGCGATAAGTAGGTTCTTATTCG | - |
| miR-2755-3p | CACCCTGTCAGACCATACTTGTT | - |
| miR-486-5p | TATCCTGTACTGAGCTGCCCC | - |
| miR-10400-5p | GGCGGCGACTCTGGAAAAA | - |
| miR-71-5p | TGAAAGACATGGGTAGTGAGATG | - |
| miR-2944-3p | TATCACAGTCCTAGTTACCTAG | - |
| miR-8-3p | CGCGTAATACTGTCAGGTAAAGAT | - |
| miR-2733-3p | CGCTCACTGGGAATGTAATAGC | - |
| miR-2778-3p | GCGCAGAGTACGCAAAAAAACAA | - |
| miR-486-3p | GGGCAGCTCAGTACAGGATAA | - |
| miR-2411-3p | TGAACTGTCTTACTCCCACATCT | - |
| miR-735-3p | GCGCCCACTGCTAAATTTGAC | - |
| U6 snRNA | GGACATCCGATAAAATTGGAAC | TTCTCGATTTGTGCGTGTCA |

Table S2 Metabolites determined by LC-MS platform under positive ion mode.

| ID | MS2 name | DCF1 | DCF2 | DCF3 | DCF4 | DCF5 | DCF6 | DCF7 | DCF8 | NCF1 | NCF2 | NCF3 | NCF4 | NCF5 | NCF6 | NCF7 | NCF8 |
| --- | --- | --- | --- | --- | --- | --- | --- | --- | --- | --- | --- | --- | --- | --- | --- | --- | --- |
| 1 | 1-Butylamine | 0.679 | 0.660 | 0.593 | 0.591 | 0.506 | 0.507 | 0.491 | 0.450 | 0.552 | 0.582 | 0.671 | 0.538 | 0.578 | 1.052 | 0.484 | 0.512 |
| 2 | Pyridine | 9.35 | 10.45 | 11.65 | 8.30 | 9.47 | 8.65 | 7.56 | 9.90 | 19.64 | 14.33 | 6.70 | 9.58 | 9.48 | 11.95 | 12.25 | 5.93 |
| 3 | 2,6-Dimethylpyridine | 0.393 | 0.382 | 0.352 | 0.380 | 0.337 | 0.352 | 0.339 | 0.318 | 0.318 | 0.348 | 0.400 | 0.348 | 0.380 | 0.714 | 0.354 | 0.368 |
| 4 | Glycine | 0.283 | 0.212 | 0.165 | 0.169 | 0.241 | 0.153 | 0.160 | 0.171 | 0.156 | 0.171 | 0.280 | 0.183 | 0.181 | 0.366 | 0.205 | 0.272 |
| 5 | Cytidine | 0.305 | 0.404 | 0.457 | 0.201 | 0.305 | 0.186 | 0.197 | 0.302 | 0.420 | 0.248 | 0.329 | 0.293 | 0.260 | 0.183 | 0.214 | 0.288 |
| 6 | N, N-Dimethylformamide | 0.065 | 0.043 | 0.048 | 0.061 | 0.057 | 0.087 | 0.041 | 0.053 | 0.053 | 0.052 | 0.049 | 0.036 | 0.044 | 0.099 | 0.053 | 0.038 |
| 7 | L-Isoleucine | 6.383 | 7.038 | 3.546 | 3.145 | 7.965 | 2.175 | 3.105 | 7.024 | 11.058 | 3.787 | 4.529 | 1.989 | 2.125 | 1.588 | 12.36 | 4.444 |
| 8 | 5-Ethyl-2-methylpyridine | 3.640 | 3.297 | 2.891 | 3.143 | 2.836 | 3.060 | 4.179 | 2.702 | 3.051 | 2.924 | 3.376 | 2.959 | 4.212 | 5.983 | 3.797 | 3.237 |
| 9 | Hypoxanthine | 5.025 | 4.322 | 4.420 | 3.304 | 3.150 | 2.747 | 3.411 | 3.751 | 0.024 | 2.688 | 3.966 | 3.438 | 2.861 | 2.305 | 3.017 | 3.960 |
| 10 | Isopropylpyrazine | 0.210 | 0.209 | 0.194 | 0.203 | 0.166 | 0.181 | 0.110 | 0.158 | 0.165 | 0.180 | 0.193 | 0.164 | 0.182 | 0.389 | 0.183 | 0.190 |
| 11 | L-Gulose | 0.195 | 0.515 | 0.490 | 0.753 | 0.289 | 0.475 | 0.570 | 0.454 | 0.320 | 0.461 | 0.320 | 0.322 | 0.517 | 0.788 | 0.389 | 0.305 |
| 12 | Choline | 15.87 | 10.96 | 14.80 | 7.90 | 12.79 | 9.80 | 7.38 | 10.34 | 12.99 | 8.50 | 12.17 | 9.98 | 9.71 | 8.37 | 11.66 | 11.75 |
| 13 | 2,6-Diaminohexanoic acid | 0.779 | 0.811 | 0.456 | 0.510 | 0.818 | 0.360 | 0.459 | 0.669 | 0.643 | 0.441 | 0.786 | 0.430 | 0.400 | 0.502 | 0.419 | 0.761 |
| 14 | 3'-AMP | 0.228 | 0.972 | 0.386 | 0.182 | 0.403 | 0.487 | 0.185 | 0.197 | 0.130 | 0.484 | 0.137 | 0.245 | 0.325 | 0.295 | 0.326 | 0.135 |
| 15 | Inosine | 0.793 | 0.452 | 0.475 | 0.637 | 0.704 | 0.427 | 0.480 | 0.666 | 1.783 | 0.772 | 0.883 | 0.739 | 0.618 | 1.379 | 0.797 | 0.758 |
| 16 | Creatinine | 0.262 | 0.252 | 0.114 | 0.098 | 0.260 | 0.095 | 0.100 | 0.135 | 0.216 | 0.219 | 0.398 | 0.091 | 0.208 | 0.107 | 0.182 | 0.337 |
| 17 | Adenosine | 0.128 | 0.107 | 0.129 | 0.040 | 0.183 | 0.070 | 0.025 | 0.045 | 0.227 | 0.118 | 0.093 | 0.111 | 0.070 | 0.125 | 0.148 | 0.089 |
| 18 | N-Methyl proline | 0.222 | 0.224 | 0.138 | 0.149 | 0.227 | 0.110 | 0.147 | 0.204 | 0.187 | 0.131 | 0.220 | 0.131 | 0.126 | 0.146 | 0.130 | 0.226 |
| 19 | 1-Deoxy-1-(N6-lysino)-D-fructose | 1.010 | 0.559 | 0.580 | 0.493 | 0.796 | 0.407 | 0.461 | 0.795 | 0.618 | 0.794 | 0.952 | 0.644 | 0.595 | 1.081 | 0.767 | 0.976 |
| 20 | 1-Pyrroline | 0.581 | 0.403 | 0.284 | 0.325 | 0.481 | 0.247 | 0.298 | 0.473 | 0.307 | 0.369 | 0.561 | 0.274 | 0.248 | 0.275 | 0.325 | 0.552 |
| 21 | Citrulline | 0.441 | 1.302 | 0.230 | 0.447 | 0.584 | 0.297 | 0.421 | 0.344 | 0.205 | 0.386 | 0.290 | 0.189 | 0.297 | 0.398 | 0.357 | 0.268 |
| 22 | L-Glutamine | 1.921 | 1.371 | 0.956 | 1.211 | 1.470 | 0.850 | 1.162 | 1.598 | 1.120 | 1.284 | 1.744 | 0.906 | 0.917 | 1.444 | 0.962 | 1.644 |
| 23 | Creatine | 29.10 | 20.55 | 13.03 | 12.54 | 19.67 | 12.62 | 10.92 | 12.75 | 13.17 | 9.32 | 9.45 | 6.72 | 10.31 | 10.39 | 7.28 | 9.10 |
| 24 | Iso-nLc8Cer | 0.240 | 0.261 | 0.202 | 0.206 | 0.169 | 0.184 | 0.191 | 0.168 | 0.197 | 0.204 | 0.232 | 0.216 | 0.194 | 0.388 | 0.198 | 0.189 |
| 25 | Pyroglutamic acid | 1.697 | 1.247 | 1.025 | 1.025 | 1.360 | 0.891 | 0.989 | 1.345 | 1.079 | 1.335 | 1.710 | 1.092 | 1.012 | 0.707 | 1.077 | 1.674 |
| 26 | DL-Glutamate | 7.087 | 4.872 | 4.649 | 3.633 | 5.527 | 3.549 | 3.570 | 4.538 | 4.883 | 6.016 | 6.369 | 4.133 | 3.946 | 6.782 | 4.122 | 5.973 |
| 27 | Nervonyl carnitine | 0.018 | 0.017 | 0.016 | 0.017 | 0.015 | 0.015 | 0.019 | 0.016 | 0.015 | 0.016 | 0.020 | 0.016 | 0.016 | 0.036 | 0.019 | 0.018 |
| 28 | Guanosine | 0.194 | 0.090 | 0.102 | 0.138 | 0.171 | 0.098 | 0.106 | 0.188 | 0.525 | 0.155 | 0.207 | 0.174 | 0.135 | 0.291 | 0.242 | 0.194 |
| 29 | Taurine | 0.751 | 0.637 | 0.542 | 0.632 | 0.463 | 0.607 | 0.567 | 0.600 | 0.349 | 0.364 | 0.608 | 0.600 | 0.353 | 1.086 | 0.582 | 0.552 |
| ID | MS2 name | DCF1 | DCF2 | DCF3 | DCF4 | DCF5 | DCF6 | DCF7 | DCF8 | NCF1 | NCF2 | NCF3 | NCF4 | NCF5 | NCF6 | NCF7 | NCF8 |
| 30 | Octadecanamide | 0.022 | 0.021 | 0.020 | 0.021 | 0.019 | 0.017 | 0.018 | 0.014 | 0.019 | 0.020 | 0.024 | 0.020 | 0.019 | 0.039 | 0.019 | 0.020 |
| 31 | 1H-Indole-3-carboxaldehyde | 0.266 | 0.515 | 0.169 | 0.200 | 0.213 | 0.120 | 0.188 | 0.194 | 0.202 | 0.117 | 0.368 | 0.128 | 0.225 | 0.022 | 0.150 | 0.337 |
| 32 | NAD | 0.029 | 0.031 | 0.017 | 0.003 | 0.017 | 0.019 | 0.003 | 0.007 | 0.019 | 0.025 | 0.016 | 0.028 | 0.018 | 0.012 | 0.031 | 0.013 |
| 33 | Niacinamide | 1.879 | 1.809 | 1.866 | 1.855 | 2.070 | 1.256 | 1.812 | 1.967 | 0.103 | 1.586 | 2.586 | 1.770 | 1.703 | 1.253 | 1.623 | 2.610 |
| 34 | Dibutyl phthalate | 0.431 | 0.518 | 0.331 | 0.279 | 0.311 | 0.391 | 0.305 | 0.238 | 0.247 | 0.274 | 0.346 | 0.377 | 0.319 | 0.618 | 0.294 | 0.279 |
| 35 | Putrescine | 0.049 | 0.067 | 0.042 | 0.055 | 0.047 | 0.054 | 0.047 | 0.072 | 0.060 | 0.067 | 0.064 | 0.065 | 0.081 | 0.069 | 0.071 | 0.055 |
| 36 | Phosphoric acid | 0.047 | 0.069 | 0.051 | 0.042 | 0.041 | 0.133 | 0.056 | 0.018 | 0.046 | 0.042 | 0.142 | 0.103 | 0.056 | 0.117 | 0.124 | 0.155 |
| 37 | Dodecanoylcarnitine | 0.013 | 0.073 | 0.070 | 0.019 | 0.068 | 0.036 | 0.017 | 0.044 | 0.052 | 0.104 | 0.085 | 0.103 | 0.152 | 0.035 | 0.039 | 0.072 |
| 38 | Ornithine | 0.099 | 0.058 | 0.041 | 0.045 | 0.068 | 0.041 | 0.045 | 0.059 | 0.057 | 0.058 | 0.118 | 0.049 | 0.047 | 0.053 | 0.061 | 0.112 |
| 39 | 2-Pentylpyridine | 0.486 | 0.428 | 0.423 | 0.401 | 0.353 | 0.352 | 0.365 | 0.415 | 0.328 | 0.390 | 0.425 | 0.369 | 0.193 | 0.748 | 0.389 | 0.371 |
| 40 | L-Phenylalanine | 7.428 | 11.629 | 6.994 | 6.856 | 9.985 | 4.605 | 6.599 | 10.411 | 8.089 | 5.254 | 7.361 | 5.133 | 4.512 | 3.148 | 4.682 | 7.377 |
| 41 | L-Histidine | 0.506 | 0.336 | 0.288 | 0.281 | 0.509 | 0.201 | 0.252 | 0.426 | 0.348 | 0.296 | 0.368 | 0.213 | 0.186 | 0.416 | 0.248 | 0.344 |
| 42 | Genistein 5-O-glucuronide | 0.008 | 0.016 | 0.013 | 0.001 | 0.044 | 0.001 | 0.001 | 0.000 | 0.000 | 0.000 | 0.000 | 0.000 | 0.000 | 0.000 | 0.000 | 0.000 |
| 43 | Verimol C | 0.040 | 0.046 | 0.054 | 0.068 | 0.039 | 0.056 | 0.057 | 0.051 | 0.043 | 0.050 | 0.040 | 0.038 | 0.054 | 0.108 | 0.037 | 0.036 |
| 44 | Guanosine monophosphate | 0.026 | 0.072 | 0.032 | 0.028 | 0.035 | 0.046 | 0.027 | 0.021 | 0.023 | 0.045 | 0.025 | 0.029 | 0.034 | 0.043 | 0.040 | 0.023 |
| 45 | Histamine | 0.148 | 0.117 | 0.109 | 0.170 | 0.163 | 0.150 | 0.160 | 0.101 | 0.089 | 0.159 | 0.162 | 0.130 | 0.079 | 0.234 | 0.094 | 0.147 |
| 46 | 2,3-Butanediol | 0.037 | 0.029 | 0.020 | 0.028 | 0.023 | 0.021 | 0.019 | 0.026 | 0.021 | 0.018 | 0.025 | 0.025 | 0.021 | 0.036 | 0.027 | 0.022 |
| 47 | Protein serine | 0.473 | 0.172 | 0.276 | 0.321 | 0.449 | 0.233 | 0.296 | 0.380 | 0.309 | 0.285 | 0.448 | 0.254 | 0.289 | 0.753 | 0.330 | 0.435 |
| 48 | Citicoline | 0.021 | 0.038 | 0.015 | 0.029 | 0.023 | 0.023 | 0.023 | 0.019 | 0.057 | 0.034 | 0.045 | 0.046 | 0.026 | 0.043 | 0.049 | 0.042 |
| 49 | 2-Acetyl-1-alkyl-sn-glycero-3-phosphocholine | 0.236 | 0.255 | 0.116 | 0.069 | 0.179 | 0.078 | 0.059 | 0.149 | 0.199 | 0.157 | 0.142 | 0.126 | 0.106 | 0.049 | 0.113 | 0.106 |
| 50 | L-Carnitine | 1.721 | 1.491 | 0.834 | 0.907 | 1.553 | 0.839 | 0.817 | 1.564 | 1.408 | 1.364 | 2.447 | 2.909 | 2.505 | 2.463 | 1.560 | 2.281 |
| 51 | L-Arginine | 0.756 | 1.030 | 0.540 | 0.756 | 0.874 | 0.527 | 0.677 | 0.674 | 0.687 | 0.488 | 0.747 | 0.463 | 0.699 | 0.666 | 0.559 | 0.727 |
| 52 | 5'-Methylthioadenosine | 0.181 | 0.182 | 0.159 | 0.174 | 0.189 | 0.155 | 0.176 | 0.167 | 0.206 | 0.280 | 0.288 | 0.266 | 0.197 | 0.282 | 0.296 | 0.287 |
| 53 | CDP-Ethanolamine | 0.005 | 0.016 | 0.005 | 0.010 | 0.007 | 0.008 | 0.007 | 0.007 | 0.019 | 0.011 | 0.015 | 0.017 | 0.012 | 0.020 | 0.018 | 0.013 |
| 54 | Sphingosine | 0.094 | 0.134 | 0.400 | 0.113 | 0.286 | 0.097 | 0.107 | 0.165 | 0.161 | 0.075 | 0.156 | 0.112 | 0.180 | 0.058 | 0.135 | 0.144 |
| 55 | Propionylcarnitine | 0.137 | 0.404 | 0.111 | 0.154 | 0.183 | 0.096 | 0.142 | 0.377 | 0.589 | 0.343 | 0.590 | 0.715 | 0.596 | 0.540 | 0.457 | 0.472 |
| 56 | Phosphorylcholine | 2.255 | 4.154 | 1.323 | 2.761 | 2.962 | 1.931 | 2.527 | 1.526 | 2.755 | 1.872 | 3.469 | 2.924 | 2.798 | 4.123 | 3.235 | 3.344 |
| 57 | (-)-Epigallocatechin | 0.242 | 1.086 | 0.138 | 0.639 | 0.262 | 1.043 | 0.843 | 0.488 | 0.043 | 0.439 | 0.153 | 0.134 | 1.008 | 0.001 | 0.410 | 0.168 |
| 58 | Racemethionine | 1.855 | 1.759 | 1.212 | 1.354 | 1.716 | 0.939 | 1.366 | 2.202 | 1.099 | 1.145 | 1.376 | 0.878 | 0.909 | 0.798 | 0.963 | 1.387 |
| 59 | 1,3,11(13)-Eudesmatrien-12-oic acid | 0.030 | 0.043 | 0.044 | 0.021 | 0.098 | 0.023 | 0.021 | 0.020 | 0.000 | 0.000 | 0.000 | 0.000 | 0.000 | 0.000 | 0.000 | 0.000 |
| 60 | L-Threonine | 1.085 | 1.040 | 0.671 | 0.656 | 1.188 | 0.504 | 0.594 | 0.765 | 0.731 | 0.563 | 0.974 | 0.529 | 0.592 | 0.750 | 0.638 | 0.931 |
| 61 | Glycerophosphocholine | 5.527 | 3.711 | 1.807 | 2.404 | 3.531 | 2.255 | 2.207 | 3.277 | 3.796 | 3.404 | 6.325 | 6.202 | 2.414 | 3.160 | 4.273 | 5.710 |
| 62 | Palmitic amide | 0.307 | 0.285 | 0.212 | 0.239 | 0.161 | 0.267 | 0.238 | 0.080 | 0.157 | 0.125 | 0.266 | 0.102 | 0.062 | 0.374 | 0.257 | 0.267 |
| 63 | (+)-2,3-Dihydro-3-methyl-1H-pyrrole | 0.029 | 0.025 | 0.023 | 0.020 | 0.029 | 0.016 | 0.019 | 0.023 | 0.026 | 0.016 | 0.033 | 0.017 | 0.018 | 0.020 | 0.018 | 0.030 |
| 64 | Phytosphingosine | 0.019 | 0.032 | 0.024 | 0.021 | 0.025 | 0.020 | 0.020 | 0.025 | 0.009 | 0.009 | 0.012 | 0.009 | 0.027 | 0.015 | 0.012 | 0.009 |
| 65 | L-Acetylcarnitine | 0.356 | 0.725 | 1.604 | 0.360 | 0.660 | 1.402 | 1.383 | 0.864 | 1.904 | 1.948 | 1.120 | 1.163 | 0.953 | 2.258 | 0.642 | 2.990 |
| 66 | Glycyl-valine | 1.052 | 0.986 | 0.121 | 0.177 | 0.571 | 0.475 | 0.450 | 0.796 | 0.858 | 1.038 | 1.353 | 0.856 | 0.880 | 0.996 | 0.553 | 1.271 |
| 67 | Decanoylcarnitine | 0.009 | 0.098 | 0.085 | 0.019 | 0.105 | 0.056 | 0.018 | 0.052 | 0.070 | 0.145 | 0.106 | 0.170 | 0.476 | 0.054 | 0.051 | 0.041 |
| 68 | Pantothenic acid | 0.254 | 0.281 | 0.318 | 0.114 | 0.210 | 0.193 | 0.103 | 0.157 | 0.178 | 0.150 | 0.158 | 0.126 | 0.134 | 0.063 | 0.106 | 0.139 |
| 69 | Saussurea lactone | 0.050 | 0.035 | 0.042 | 0.036 | 0.036 | 0.030 | 0.029 | 0.034 | 0.049 | 0.033 | 0.031 | 0.030 | 0.032 | 0.057 | 0.030 | 0.029 |
| 70 | Quinaldic acid | 0.116 | 0.147 | 0.073 | 0.069 | 0.087 | 0.041 | 0.058 | 0.063 | 0.079 | 0.038 | 0.099 | 0.033 | 0.055 | 0.004 | 0.044 | 0.067 |
| 71 | (Â±)-Tryptophan | 0.487 | 0.668 | 0.845 | 0.767 | 1.065 | 0.532 | 0.755 | 0.902 | 0.653 | 0.553 | 0.438 | 0.433 | 0.375 | 0.746 | 0.507 | 0.459 |
| 72 | LysoPE (16:0/0:0) | 0.226 | 0.425 | 0.245 | 0.157 | 0.248 | 0.151 | 0.138 | 0.283 | 0.512 | 0.294 | 0.318 | 0.241 | 0.217 | 0.099 | 0.266 | 0.272 |
| 73 | MG (18:2(9Z,12Z)/0:0/0:0) | 0.008 | 0.009 | 0.006 | 0.002 | 0.008 | 0.004 | 0.003 | 0.007 | 0.009 | 0.006 | 0.007 | 0.003 | 0.003 | 0.005 | 0.003 | 0.007 |
| 74 | 2,5-Dihydro-2,4,5-trimethyloxazole | 0.098 | 0.247 | 0.052 | 0.075 | 0.063 | 0.070 | 0.064 | 0.063 | 0.171 | 0.077 | 0.083 | 0.048 | 0.072 | 0.154 | 0.062 | 0.066 |
| 75 | Epsilon-caprolactam | 0.143 | 0.063 | 0.088 | 0.049 | 0.078 | 0.077 | 0.075 | 0.070 | 0.091 | 0.152 | 0.089 | 0.079 | 0.085 | 0.163 | 0.073 | 0.085 |
| 76 | Arachidyl carnitine | 0.031 | 0.051 | 0.047 | 0.029 | 0.036 | 0.024 | 0.024 | 0.049 | 0.063 | 0.045 | 0.043 | 0.040 | 0.030 | 0.026 | 0.073 | 0.034 |
| 77 | [8]-Gingerol | 0.051 | 0.042 | 0.043 | 0.043 | 0.039 | 0.041 | 0.041 | 0.040 | 0.042 | 0.043 | 0.047 | 0.043 | 0.045 | 0.087 | 0.040 | 0.040 |
| 78 | LysoPE (18:1(9Z)/0:0) | 0.603 | 1.035 | 0.678 | 0.375 | 0.673 | 0.504 | 0.316 | 0.638 | 1.159 | 0.910 | 0.832 | 0.579 | 0.693 | 0.235 | 0.605 | 0.703 |
| 79 | gamma-Glutamylcysteine | 0.001 | 0.011 | 0.000 | 0.020 | 0.002 | 0.037 | 0.018 | 0.003 | 0.000 | 0.000 | 0.000 | 0.000 | 0.043 | 0.022 | 0.010 | 0.000 |
| 80 | gamma-Glutamylleucine | 0.533 | 0.201 | 0.464 | 0.174 | 0.550 | 0.128 | 0.185 | 0.351 | 0.352 | 0.229 | 0.393 | 0.271 | 0.157 | 0.085 | 0.235 | 0.384 |
| 81 | Muricatacin | 0.002 | 0.006 | 0.003 | 0.002 | 0.005 | 0.002 | 0.002 | 0.014 | 0.014 | 0.007 | 0.011 | 0.007 | 0.020 | 0.005 | 0.004 | 0.010 |
| 82 | 2-Acetylpyrrolidine | 0.145 | 0.088 | 0.063 | 0.048 | 0.053 | 0.051 | 0.052 | 0.049 | 0.071 | 0.076 | 0.062 | 0.057 | 0.056 | 0.110 | 0.051 | 0.053 |
| 83 | Glutathione | 0.014 | 0.081 | 0.015 | 0.031 | 0.013 | 0.157 | 0.040 | 0.028 | 0.010 | 0.028 | 0.029 | 0.021 | 0.093 | 0.401 | 0.026 | 0.035 |
| 84 | N2-gamma-Glutamylglutamine | 0.452 | 0.093 | 0.161 | 0.163 | 0.288 | 0.089 | 0.154 | 0.289 | 0.181 | 0.214 | 0.384 | 0.192 | 0.111 | 0.086 | 0.156 | 0.386 |
| 85 | Cadabicilone | 0.101 | 0.158 | 0.160 | 0.077 | 0.358 | 0.081 | 0.074 | 0.063 | 0.004 | 0.000 | 0.002 | 0.000 | 0.000 | 0.000 | 0.000 | 0.003 |
| 86 | Xanthine | 0.112 | 0.029 | 0.046 | 0.130 | 0.028 | 0.145 | 0.186 | 0.030 | 0.010 | 0.027 | 0.194 | 0.117 | 0.133 | 0.067 | 0.032 | 0.233 |
| 87 | Acetylcholine | 0.193 | 0.306 | 0.181 | 0.285 | 0.225 | 0.226 | 0.251 | 0.338 | 0.230 | 0.350 | 0.347 | 0.303 | 0.269 | 0.492 | 0.201 | 0.311 |
| ID | MS2 name | DCF1 | DCF2 | DCF3 | DCF4 | DCF5 | DCF6 | DCF7 | DCF8 | NCF1 | NCF2 | NCF3 | NCF4 | NCF5 | NCF6 | NCF7 | NCF8 |
| 88 | Oleamide | 0.017 | 0.025 | 0.077 | 0.020 | 0.057 | 0.018 | 0.019 | 0.033 | 0.031 | 0.015 | 0.028 | 0.020 | 0.034 | 0.009 | 0.026 | 0.029 |
| 89 | 2-Isopropylphenyl methylcarbamate | 0.675 | 0.419 | 0.609 | 0.577 | 0.544 | 0.387 | 0.529 | 0.450 | 0.499 | 0.416 | 0.717 | 0.628 | 0.396 | 1.108 | 0.587 | 0.374 |
| 90 | Sarcosine | 1.444 | 1.378 | 0.781 | 0.972 | 1.186 | 0.918 | 0.923 | 1.001 | 0.828 | 0.957 | 0.993 | 0.749 | 0.817 | 1.115 | 0.751 | 0.968 |
| 91 | Hypoletin 8-gentiobioside | 0.007 | 0.010 | 0.008 | 0.003 | 0.023 | 0.006 | 0.004 | 0.003 | 0.010 | 0.005 | 0.003 | 0.003 | 0.024 | 0.000 | 0.009 | 0.003 |
| 92 | L-Palmitoylcarnitine | 0.153 | 0.204 | 0.288 | 0.155 | 0.260 | 0.162 | 0.147 | 0.280 | 0.438 | 0.313 | 0.494 | 0.776 | 0.853 | 0.307 | 0.290 | 0.456 |
| 93 | Stearoylcarnitine | 0.136 | 0.196 | 0.226 | 0.141 | 0.226 | 0.127 | 0.131 | 0.241 | 0.332 | 0.232 | 0.332 | 0.441 | 0.403 | 0.210 | 0.316 | 0.316 |
| 94 | Piperidine | 0.837 | 0.840 | 0.499 | 0.484 | 0.955 | 0.399 | 0.433 | 3.555 | 1.196 | 0.509 | 0.691 | 0.361 | 0.389 | 0.312 | 0.537 | 1.567 |
| 95 | LysoPE (16:1(9Z)/0:0) | 0.016 | 0.020 | 0.016 | 0.006 | 0.015 | 0.012 | 0.004 | 0.015 | 0.084 | 0.040 | 0.037 | 0.029 | 0.035 | 0.007 | 0.034 | 0.034 |
| 96 | Beta-Guanidinopropionic acid | 0.226 | 0.207 | 1.161 | 0.949 | 0.227 | 1.053 | 0.944 | 1.164 | 1.161 | 0.940 | 2.199 | 0.576 | 0.749 | 0.556 | 0.678 | 1.291 |
| 97 | Adenine | 0.014 | 0.014 | 0.022 | 0.011 | 0.009 | 0.010 | 0.010 | 0.013 | 0.008 | 0.023 | 0.011 | 0.005 | 0.011 | 0.022 | 0.009 | 0.010 |
| 98 | PC(22:2(13Z,16Z)/14:1(9Z)) | 0.007 | 0.003 | 0.005 | 0.011 | 0.048 | 0.083 | 0.092 | 0.079 | 0.009 | 0.004 | 0.003 | 0.013 | 0.075 | 0.110 | 0.089 | 0.100 |
| 99 | (13E)-11a-Hydroxy-9,15-dioxoprost-13-enoic acid | 0.017 | 0.020 | 0.016 | 0.017 | 0.017 | 0.014 | 0.015 | 0.014 | 0.018 | 0.019 | 0.017 | 0.015 | 0.010 | 0.023 | 0.014 | 0.015 |
| 100 | Glutamylglutamic acid | 0.055 | 0.010 | 0.039 | 0.014 | 0.042 | 0.011 | 0.014 | 0.031 | 0.051 | 0.039 | 0.055 | 0.036 | 0.016 | 0.021 | 0.026 | 0.052 |
| 101 | Argininosuccinic acid | 0.054 | 0.048 | 0.014 | 0.044 | 0.035 | 0.016 | 0.041 | 0.049 | 0.020 | 0.025 | 0.045 | 0.016 | 0.034 | 0.020 | 0.013 | 0.045 |
| 102 | L-Proline | 11.053 | 6.660 | 4.496 | 5.676 | 8.980 | 3.664 | 5.259 | 8.782 | 4.703 | 6.395 | 11.403 | 4.603 | 3.841 | 5.249 | 5.950 | 10.628 |
| 103 | Palmitoyl serinol | 0.026 | 0.045 | 0.023 | 0.015 | 0.031 | 0.022 | 0.015 | 0.029 | 0.005 | 0.006 | 0.022 | 0.012 | 0.037 | 0.006 | 0.012 | 0.019 |
| 104 | LysoPC (22:6(4Z,7Z,10Z,13Z,16Z,19Z)) | 0.007 | 0.008 | 0.014 | 0.004 | 0.022 | 0.002 | 0.005 | 0.003 | 0.017 | 0.011 | 0.008 | 0.018 | 0.004 | 0.004 | 0.008 | 0.009 |
| 105 | Aspartyl-isoleucine | 0.169 | 0.045 | 0.118 | 0.040 | 0.144 | 0.032 | 0.038 | 0.084 | 0.090 | 0.076 | 0.075 | 0.077 | 0.048 | 0.028 | 0.077 | 0.073 |
| 106 | 2-Phenylacetamide | 0.149 | 0.182 | 0.128 | 0.156 | 0.166 | 0.109 | 0.151 | 0.197 | 0.215 | 0.133 | 0.119 | 0.082 | 0.090 | 0.175 | 0.151 | 0.119 |
| 107 | Avocadyne | 0.642 | 0.609 | 0.561 | 0.582 | 0.530 | 0.529 | 0.529 | 0.510 | 0.553 | 0.566 | 0.619 | 0.554 | 0.584 | 1.093 | 0.541 | 0.563 |
| 108 | Dehydrophytosphingosine | 0.031 | 0.032 | 0.031 | 0.029 | 0.033 | 0.027 | 0.028 | 0.029 | 0.024 | 0.027 | 0.033 | 0.032 | 0.029 | 0.058 | 0.029 | 0.029 |
| 109 | LysoPC (14:0/0:0) | 0.081 | 0.060 | 0.054 | 0.018 | 0.041 | 0.026 | 0.017 | 0.117 | 0.115 | 0.091 | 0.070 | 0.071 | 0.076 | 0.029 | 0.070 | 0.063 |
| 110 | LysoPC (18:3(6Z,9Z,12Z)) | 0.035 | 0.022 | 0.027 | 0.007 | 0.041 | 0.021 | 0.007 | 0.018 | 0.026 | 0.020 | 0.013 | 0.017 | 0.015 | 0.004 | 0.017 | 0.009 |
| 111 | 7-Ethoxy-4-methyl-2H-1-benzopyran-2-one | 0.120 | 0.112 | 0.095 | 0.112 | 0.054 | 0.079 | 0.101 | 0.043 | 0.065 | 0.079 | 0.096 | 0.049 | 0.112 | 0.169 | 0.053 | 0.079 |
| 112 | N-Acetyl-2,6-diethylaniline | 0.170 | 0.214 | 0.134 | 0.159 | 0.138 | 0.140 | 0.144 | 0.124 | 0.129 | 0.199 | 0.218 | 0.145 | 0.159 | 0.363 | 0.147 | 0.141 |
| 113 | Ophthalmic acid | 0.004 | 0.026 | 0.010 | 0.018 | 0.006 | 0.021 | 0.017 | 0.024 | 0.001 | 0.035 | 0.017 | 0.016 | 0.039 | 0.020 | 0.038 | 0.017 |
| ID | MS2 name | DCF1 | DCF2 | DCF3 | DCF4 | DCF5 | DCF6 | DCF7 | DCF8 | NCF1 | NCF2 | NCF3 | NCF4 | NCF5 | NCF6 | NCF7 | NCF8 |
| 114 | Norcodeine | 0.019 | 0.017 | 0.014 | 0.015 | 0.013 | 0.013 | 0.011 | 0.010 | 0.015 | 0.016 | 0.017 | 0.015 | 0.015 | 0.029 | 0.012 | 0.013 |
| 115 | Succinyladenosine | 0.109 | 0.017 | 0.033 | 0.036 | 0.037 | 0.034 | 0.035 | 0.119 | 0.041 | 0.053 | 0.100 | 0.038 | 0.043 | 0.017 | 0.070 | 0.102 |
| 116 | L-Octanoylcarnitine | 0.015 | 0.194 | 0.105 | 0.063 | 0.084 | 0.103 | 0.057 | 0.099 | 0.051 | 0.157 | 0.136 | 0.140 | 0.422 | 0.114 | 0.095 | 0.120 |
| 117 | Oxidized glutathione | 0.026 | 0.099 | 0.010 | 0.048 | 0.018 | 0.072 | 0.061 | 0.032 | 0.004 | 0.047 | 0.012 | 0.010 | 0.081 | 0.000 | 0.031 | 0.012 |
| 118 | Taurocholic acid | 0.766 | 1.233 | 1.457 | 0.610 | 2.250 | 0.528 | 0.596 | 0.680 | 1.130 | 0.792 | 0.869 | 0.982 | 1.305 | 0.315 | 1.294 | 0.854 |
| 119 | Aspartame | 0.170 | 0.073 | 0.200 | 0.062 | 0.177 | 0.045 | 0.060 | 0.119 | 0.151 | 0.081 | 0.146 | 0.112 | 0.057 | 0.027 | 0.082 | 0.142 |
| 120 | Homodihydrocapsaicin | 0.026 | 0.026 | 0.024 | 0.024 | 0.021 | 0.021 | 0.019 | 0.018 | 0.021 | 0.024 | 0.027 | 0.019 | 0.019 | 0.048 | 0.019 | 0.022 |
| 121 | Yuccaol C | 0.013 | 0.001 | 0.016 | 0.000 | 0.055 | 0.000 | 0.000 | 0.001 | 0.001 | 0.002 | 0.004 | 0.000 | 0.000 | 0.000 | 0.003 | 0.003 |
| 122 | MG (0:0/14:0/0:0) | 0.005 | 0.012 | 0.007 | 0.002 | 0.006 | 0.002 | 0.002 | 0.010 | 0.021 | 0.006 | 0.011 | 0.006 | 0.019 | 0.008 | 0.005 | 0.010 |
| 123 | DL-2-Aminooctanoic acid | 1.086 | 0.574 | 0.388 | 0.526 | 0.560 | 0.309 | 0.486 | 0.988 | 0.365 | 0.813 | 1.276 | 0.814 | 1.031 | 0.991 | 0.896 | 1.124 |
| 124 | Uridine diphosphate-N-acetylgalactosamine | 0.006 | 0.005 | 0.006 | 0.005 | 0.004 | 0.007 | 0.006 | 0.006 | 0.041 | 0.019 | 0.013 | 0.011 | 0.012 | 0.013 | 0.010 | 0.018 |
| 125 | LysoPC (17:0) | 0.116 | 0.108 | 0.062 | 0.037 | 0.076 | 0.041 | 0.031 | 0.061 | 0.134 | 0.085 | 0.097 | 0.087 | 0.080 | 0.031 | 0.074 | 0.080 |
| 126 | LysoPC (18:2(9Z,12Z)) | 0.894 | 0.693 | 0.825 | 0.269 | 0.971 | 0.418 | 0.252 | 0.387 | 0.491 | 0.353 | 0.195 | 0.294 | 0.319 | 0.144 | 0.226 | 0.171 |
| 127 | Homocitrulline | 0.080 | 0.109 | 0.012 | 0.032 | 0.056 | 0.145 | 0.034 | 0.215 | 0.449 | 0.543 | 0.787 | 0.446 | 0.273 | 0.704 | 0.202 | 0.776 |
| 128 | MG (20:4(5Z,8Z,11Z,14Z)/0:0/0:0) | 0.007 | 0.005 | 0.006 | 0.002 | 0.006 | 0.003 | 0.002 | 0.004 | 0.006 | 0.008 | 0.004 | 0.003 | 0.003 | 0.003 | 0.003 | 0.003 |
| 129 | LysoPC (16:1(9Z)/0:0) | 0.057 | 0.057 | 0.039 | 0.017 | 0.061 | 0.028 | 0.015 | 0.042 | 0.146 | 0.097 | 0.079 | 0.078 | 0.075 | 0.030 | 0.077 | 0.067 |
| 130 | Maltotriose | 0.008 | 0.000 | 0.029 | 0.000 | 0.042 | 0.000 | 0.000 | 0.002 | 0.000 | 0.003 | 0.005 | 0.001 | 0.000 | 0.000 | 0.003 | 0.003 |
| 131 | gamma-Glutamyltyrosine | 0.115 | 0.039 | 0.099 | 0.048 | 0.099 | 0.030 | 0.043 | 0.077 | 0.065 | 0.048 | 0.068 | 0.054 | 0.030 | 0.021 | 0.048 | 0.068 |
| 132 | Asparaginyl-phenylalanine | 0.126 | 0.190 | 0.222 | 0.130 | 0.311 | 0.125 | 0.143 | 0.158 | 0.155 | 0.131 | 0.162 | 0.176 | 0.228 | 0.086 | 0.128 | 0.193 |
| 133 | 1-Phenyl-1,3-heneicosanedione | 0.039 | 0.049 | 0.072 | 0.037 | 0.064 | 0.040 | 0.035 | 0.068 | 0.106 | 0.079 | 0.121 | 0.199 | 0.219 | 0.072 | 0.070 | 0.110 |
| 134 | cis-5-Tetradecenoylcarnitine | 0.001 | 0.015 | 0.013 | 0.005 | 0.028 | 0.011 | 0.004 | 0.012 | 0.015 | 0.024 | 0.019 | 0.019 | 0.023 | 0.006 | 0.005 | 0.016 |
| 135 | 4-Hydroxy-1H-indole-3-acetonitrile | 0.109 | 0.137 | 0.094 | 0.085 | 0.153 | 0.055 | 0.079 | 0.110 | 0.099 | 0.041 | 0.044 | 0.051 | 0.070 | 0.090 | 0.053 | 0.080 |
| 136 | LysoPC (20:3(5Z,8Z,11Z)) | 0.037 | 0.014 | 0.038 | 0.008 | 0.021 | 0.015 | 0.006 | 0.021 | 0.073 | 0.035 | 0.020 | 0.042 | 0.020 | 0.011 | 0.027 | 0.035 |
| 137 | trans-Hexadec-2-enoyl carnitine | 0.008 | 0.029 | 0.027 | 0.021 | 0.031 | 0.018 | 0.010 | 0.019 | 0.045 | 0.037 | 0.042 | 0.042 | 0.064 | 0.019 | 0.016 | 0.039 |
| 138 | Momordenol | 0.018 | 0.039 | 0.051 | 0.023 | 0.050 | 0.027 | 0.021 | 0.029 | 0.061 | 0.045 | 0.072 | 0.053 | 0.179 | 0.032 | 0.038 | 0.066 |
| 139 | N-Acetylneuraminic acid | 0.025 | 0.026 | 0.028 | 0.030 | 0.050 | 0.019 | 0.030 | 0.025 | 0.037 | 0.036 | 0.040 | 0.038 | 0.032 | 0.051 | 0.032 | 0.031 |
| 140 | 6-Chloro-N-(1-methylethyl)-1,3,5-triazine-2,4-diamine | 0.218 | 0.311 | 0.413 | 0.367 | 0.253 | 0.254 | 0.373 | 0.456 | 0.321 | 0.251 | 0.205 | 0.197 | 0.170 | 0.349 | 0.243 | 0.214 |
| ID | MS2 name | DCF1 | DCF2 | DCF3 | DCF4 | DCF5 | DCF6 | DCF7 | DCF8 | NCF1 | NCF2 | NCF3 | NCF4 | NCF5 | NCF6 | NCF7 | NCF8 |
| 141 | LysoPE (0:0/22:5(4Z,7Z,10Z,13Z,16Z)) | 0.023 | 0.033 | 0.041 | 0.025 | 0.033 | 0.018 | 0.021 | 0.027 | 0.071 | 0.035 | 0.034 | 0.022 | 0.022 | 0.028 | 0.026 | 0.030 |
| 142 | Testosterone | 0.053 | 0.049 | 0.049 | 0.057 | 0.037 | 0.045 | 0.045 | 0.043 | 0.034 | 0.041 | 0.048 | 0.045 | 0.043 | 0.094 | 0.045 | 0.045 |
| 143 | 3,4-Dihydroxyphenyllactic acid methyl ester | 0.053 | 0.077 | 0.025 | 0.042 | 0.025 | 0.025 | 0.035 | 0.034 | 0.049 | 0.028 | 0.050 | 0.030 | 0.039 | 0.031 | 0.036 | 0.043 |
| 144 | gamma-Glutamylalanine | 0.254 | 0.095 | 0.117 | 0.115 | 0.185 | 0.097 | 0.119 | 0.160 | 0.119 | 0.149 | 0.203 | 0.150 | 0.111 | 0.074 | 0.125 | 0.206 |
| 145 | Deoxycholic acid | 0.004 | 0.007 | 0.034 | 0.001 | 0.018 | 0.000 | 0.000 | 0.000 | 0.002 | 0.003 | 0.000 | 0.000 | 0.005 | 0.000 | 0.005 | 0.001 |
| 146 | 2-Methylpropan-2-ol | 0.005 | 0.005 | 0.007 | 0.013 | 0.006 | 0.011 | 0.009 | 0.006 | 0.006 | 0.006 | 0.006 | 0.010 | 0.009 | 0.025 | 0.010 | 0.006 |
| 147 | LysoPE (0:0/22:4(7Z,10Z,13Z,16Z)) | 0.013 | 0.015 | 0.016 | 0.010 | 0.009 | 0.013 | 0.008 | 0.005 | 0.022 | 0.017 | 0.008 | 0.009 | 0.014 | 0.003 | 0.008 | 0.007 |
| 148 | Elaidic carnitine | 0.071 | 0.143 | 0.192 | 0.085 | 0.186 | 0.108 | 0.081 | 0.108 | 0.226 | 0.169 | 0.269 | 0.389 | 0.661 | 0.131 | 0.146 | 0.251 |
| 149 | Coriandrin | 0.074 | 0.064 | 0.021 | 0.043 | 0.055 | 0.016 | 0.022 | 0.055 | 0.043 | 0.031 | 0.059 | 0.035 | 0.018 | 0.059 | 0.038 | 0.056 |
| 150 | LysoPE (0:0/18:0) | 0.330 | 0.629 | 0.330 | 0.227 | 0.262 | 0.222 | 0.194 | 0.386 | 0.478 | 0.346 | 0.342 | 0.255 | 0.283 | 0.107 | 0.227 | 0.260 |
| 151 | 1-Hydroxy-3-methyl-9H-carbazole | 0.037 | 0.038 | 0.038 | 0.037 | 0.035 | 0.037 | 0.036 | 0.036 | 0.030 | 0.033 | 0.041 | 0.037 | 0.040 | 0.081 | 0.037 | 0.037 |
| 152 | [6]-Gingerdiol 3-acetate | 0.012 | 0.010 | 0.011 | 0.010 | 0.009 | 0.010 | 0.010 | 0.010 | 0.011 | 0.011 | 0.012 | 0.010 | 0.013 | 0.022 | 0.010 | 0.012 |
| 153 | (R)-N-Methylsalsolinol | 0.103 | 0.086 | 0.063 | 0.087 | 0.065 | 0.071 | 0.084 | 0.065 | 0.076 | 0.085 | 0.090 | 0.078 | 0.070 | 0.164 | 0.066 | 0.067 |
| 154 | Homo-L-arginine | 0.009 | 0.013 | 0.012 | 0.014 | 0.018 | 0.012 | 0.011 | 0.008 | 0.099 | 0.080 | 0.130 | 0.067 | 0.075 | 0.156 | 0.052 | 0.122 |
| 155 | gamma-Glutamylisoleucine | 0.016 | 0.001 | 0.010 | 0.001 | 0.015 | 0.002 | 0.003 | 0.002 | 0.002 | 0.003 | 0.037 | 0.021 | 0.007 | 0.000 | 0.008 | 0.035 |
| 156 | LysoPC (15:0) | 0.026 | 0.026 | 0.018 | 0.007 | 0.019 | 0.009 | 0.009 | 0.034 | 0.041 | 0.031 | 0.032 | 0.024 | 0.023 | 0.007 | 0.027 | 0.029 |
| 157 | 1,4-Benzodioxin-2(3H)-one | 0.079 | 0.085 | 0.081 | 0.101 | 0.061 | 0.096 | 0.114 | 0.065 | 0.045 | 0.095 | 0.095 | 0.078 | 0.146 | 0.700 | 0.087 | 0.085 |
| 158 | 2,2,4,4-Tetramethyl-6-(1-oxobutyl)-1,3,5-cyclohexanetrione | 0.035 | 0.041 | 0.037 | 0.014 | 0.085 | 0.023 | 0.016 | 0.016 | 0.033 | 0.027 | 0.018 | 0.013 | 0.079 | 0.000 | 0.041 | 0.015 |
| 159 | LysoPE (22:6(4Z,7Z,10Z,13Z,16Z,19Z)/0:0) | 0.009 | 0.020 | 0.025 | 0.015 | 0.020 | 0.015 | 0.016 | 0.012 | 0.043 | 0.018 | 0.018 | 0.016 | 0.010 | 0.044 | 0.020 | 0.015 |
| 160 | Ethyl acetate | 0.025 | 0.022 | 0.021 | 0.028 | 0.023 | 0.018 | 0.017 | 0.023 | 0.020 | 0.034 | 0.020 | 0.020 | 0.019 | 0.045 | 0.024 | 0.018 |
| 161 | Phosphocreatinine | 0.043 | 0.034 | 0.015 | 0.016 | 0.049 | 0.013 | 0.014 | 0.029 | 0.033 | 0.017 | 0.023 | 0.015 | 0.014 | 0.028 | 0.017 | 0.022 |
| 162 | 6,10,14-Trimethyl-5,9,13-pentadecatrien-2-one | 0.018 | 0.032 | 0.025 | 0.026 | 0.018 | 0.030 | 0.010 | 0.010 | 0.017 | 0.020 | 0.039 | 0.011 | 0.007 | 0.034 | 0.026 | 0.035 |
| 163 | Linoelaidyl carnitine | 0.012 | 0.054 | 0.044 | 0.014 | 0.038 | 0.013 | 0.013 | 0.012 | 0.017 | 0.012 | 0.016 | 0.019 | 0.020 | 0.035 | 0.014 | 0.009 |
| ID | MS2 name | DCF1 | DCF2 | DCF3 | DCF4 | DCF5 | DCF6 | DCF7 | DCF8 | NCF1 | NCF2 | NCF3 | NCF4 | NCF5 | NCF6 | NCF7 | NCF8 |
| 164 | Heptadecanoyl carnitine | 0.012 | 0.008 | 0.020 | 0.009 | 0.017 | 0.005 | 0.007 | 0.028 | 0.037 | 0.030 | 0.051 | 0.072 | 0.106 | 0.015 | 0.032 | 0.045 |
| 165 | Cyclotetradecane | 0.257 | 0.405 | 0.449 | 0.378 | 0.356 | 0.178 | 0.190 | 0.323 | 0.367 | 0.228 | 0.244 | 0.385 | 0.220 | 0.729 | 0.347 | 0.199 |
| 166 | (R)-Higenamine | 0.016 | 0.015 | 0.014 | 0.014 | 0.011 | 0.013 | 0.011 | 0.013 | 0.012 | 0.013 | 0.017 | 0.014 | 0.012 | 0.030 | 0.012 | 0.014 |
| 167 | 3-Mercapto-3-methyl-1-butyl acetate | 0.024 | 0.018 | 0.016 | 0.018 | 0.024 | 0.022 | 0.019 | 0.015 | 0.024 | 0.031 | 0.037 | 0.041 | 0.031 | 0.019 | 0.041 | 0.034 |
| 168 | Phenylalanyl-threonine | 0.124 | 0.487 | 0.305 | 0.196 | 0.304 | 0.193 | 0.186 | 0.367 | 0.544 | 0.389 | 0.382 | 0.289 | 0.274 | 0.129 | 0.344 | 0.356 |
| 169 | LysoPE (20:4(8Z,11Z,14Z,17Z)/0:0) | 0.099 | 0.102 | 0.130 | 0.065 | 0.084 | 0.096 | 0.057 | 0.077 | 0.158 | 0.127 | 0.092 | 0.088 | 0.163 | 0.054 | 0.085 | 0.076 |

LC-MS = liquid chromatograph-mass spectrometer; NCF = normal colostrum feeding; DCF = delayed colostrum feeding.Table S3 Metabolites determined by LC-MS platform under negative ion mode.

| ID | MS2 name | DCF1 | DCF2 | DCF3 | DCF4 | DCF5 | DCF6 | DCF7 | DCF8 | NCF1 | NCF2 | NCF3 | NCF4 | NCF5 | NCF6 | NCF7 | NCF8 |
| --- | --- | --- | --- | --- | --- | --- | --- | --- | --- | --- | --- | --- | --- | --- | --- | --- | --- |
| 1 | Glyceric acid | 0.68 | 0.66 | 0.59 | 0.59 | 0.51 | 0.51 | 0.49 | 0.45 | 0.55 | 0.58 | 0.67 | 0.54 | 0.58 | 1.05 | 0.48 | 0.51 |
| 2 | Dodecanoic acid | 9.35 | 10.45 | 11.65 | 8.30 | 9.47 | 8.65 | 7.56 | 9.90 | 19.64 | 14.33 | 6.70 | 9.58 | 9.48 | 11.95 | 12.25 | 5.93 |
| 3 | 1H-Indole-3-carboxaldehyde | 0.39 | 0.38 | 0.35 | 0.38 | 0.34 | 0.35 | 0.34 | 0.32 | 0.32 | 0.35 | 0.40 | 0.35 | 0.38 | 0.71 | 0.35 | 0.37 |
| 4 | L-Norleucine | 0.28 | 0.21 | 0.16 | 0.17 | 0.24 | 0.15 | 0.16 | 0.17 | 0.16 | 0.17 | 0.28 | 0.18 | 0.18 | 0.37 | 0.21 | 0.27 |
| 5 | Capric acid | 0.31 | 0.40 | 0.46 | 0.20 | 0.30 | 0.19 | 0.20 | 0.30 | 0.42 | 0.25 | 0.33 | 0.29 | 0.26 | 0.18 | 0.21 | 0.29 |
| 6 | Citrulline | 0.07 | 0.04 | 0.05 | 0.06 | 0.06 | 0.09 | 0.04 | 0.05 | 0.05 | 0.05 | 0.05 | 0.04 | 0.04 | 0.10 | 0.05 | 0.04 |
| 7 | Stearic acid | 6.38 | 7.04 | 3.55 | 3.15 | 7.97 | 2.17 | 3.11 | 7.02 | 11.06 | 3.79 | 4.53 | 1.99 | 2.13 | 1.59 | 12.36 | 4.44 |
| 8 | Taurine | 3.64 | 3.30 | 2.89 | 3.14 | 2.84 | 3.06 | 4.18 | 2.70 | 3.05 | 2.92 | 3.38 | 2.96 | 4.21 | 5.98 | 3.80 | 3.24 |
| 9 | L-Proline | 5.02 | 4.32 | 4.42 | 3.30 | 3.15 | 2.75 | 3.41 | 3.75 | 0.02 | 2.69 | 3.97 | 3.44 | 2.86 | 2.30 | 3.02 | 3.96 |
| 10 | O-Phosphoethanolamine | 0.21 | 0.21 | 0.19 | 0.20 | 0.17 | 0.18 | 0.11 | 0.16 | 0.16 | 0.18 | 0.19 | 0.16 | 0.18 | 0.39 | 0.18 | 0.19 |
| 11 | Palmitoleic acid | 0.20 | 0.52 | 0.49 | 0.75 | 0.29 | 0.47 | 0.57 | 0.45 | 0.32 | 0.46 | 0.32 | 0.32 | 0.52 | 0.79 | 0.39 | 0.31 |
| 12 | Hypoxanthine | 15.87 | 10.96 | 14.80 | 7.90 | 12.79 | 9.80 | 7.38 | 10.34 | 12.99 | 8.50 | 12.17 | 9.98 | 9.71 | 8.37 | 11.66 | 11.75 |
| 13 | Myristic acid | 0.78 | 0.81 | 0.46 | 0.51 | 0.82 | 0.36 | 0.46 | 0.67 | 0.64 | 0.44 | 0.79 | 0.43 | 0.40 | 0.50 | 0.42 | 0.76 |
| 14 | D-Glutamic acid | 0.23 | 0.97 | 0.39 | 0.18 | 0.40 | 0.49 | 0.19 | 0.20 | 0.13 | 0.48 | 0.14 | 0.24 | 0.33 | 0.30 | 0.33 | 0.13 |
| 15 | 2-Hydroxystearic acid | 0.79 | 0.45 | 0.48 | 0.64 | 0.70 | 0.43 | 0.48 | 0.67 | 1.78 | 0.77 | 0.88 | 0.74 | 0.62 | 1.38 | 0.80 | 0.76 |
| 16 | Maleic acid | 0.26 | 0.25 | 0.11 | 0.10 | 0.26 | 0.09 | 0.10 | 0.13 | 0.22 | 0.22 | 0.40 | 0.09 | 0.21 | 0.11 | 0.18 | 0.34 |
| 17 | Pentadecanoic acid | 0.13 | 0.11 | 0.13 | 0.04 | 0.18 | 0.07 | 0.03 | 0.04 | 0.23 | 0.12 | 0.09 | 0.11 | 0.07 | 0.12 | 0.15 | 0.09 |
| 18 | L-Arginine | 0.22 | 0.22 | 0.14 | 0.15 | 0.23 | 0.11 | 0.15 | 0.20 | 0.19 | 0.13 | 0.22 | 0.13 | 0.13 | 0.15 | 0.13 | 0.23 |
| 19 | (R)-lipoic acid | 1.01 | 0.56 | 0.58 | 0.49 | 0.80 | 0.41 | 0.46 | 0.80 | 0.62 | 0.79 | 0.95 | 0.64 | 0.59 | 1.08 | 0.77 | 0.98 |
| 20 | Myristoleic acid | 0.58 | 0.40 | 0.28 | 0.32 | 0.48 | 0.25 | 0.30 | 0.47 | 0.31 | 0.37 | 0.56 | 0.27 | 0.25 | 0.27 | 0.33 | 0.55 |
| 21 | Pyroglutamic acid | 0.44 | 1.30 | 0.23 | 0.45 | 0.58 | 0.30 | 0.42 | 0.34 | 0.21 | 0.39 | 0.29 | 0.19 | 0.30 | 0.40 | 0.36 | 0.27 |
| 22 | Gamma-Linolenic acid | 1.92 | 1.37 | 0.96 | 1.21 | 1.47 | 0.85 | 1.16 | 1.60 | 1.12 | 1.28 | 1.74 | 0.91 | 0.92 | 1.44 | 0.96 | 1.64 |
| 23 | Xanthine | 29.10 | 20.55 | 13.03 | 12.53 | 19.67 | 12.61 | 10.92 | 12.75 | 13.16 | 9.32 | 9.45 | 6.72 | 10.31 | 10.39 | 7.28 | 9.10 |
| 24 | Oleic acid | 0.24 | 0.26 | 0.20 | 0.21 | 0.17 | 0.18 | 0.19 | 0.17 | 0.20 | 0.20 | 0.23 | 0.22 | 0.19 | 0.39 | 0.20 | 0.19 |
| 25 | Malic acid | 1.70 | 1.25 | 1.02 | 1.03 | 1.36 | 0.89 | 0.99 | 1.35 | 1.08 | 1.33 | 1.71 | 1.09 | 1.01 | 0.71 | 1.08 | 1.67 |
| 26 | Arachidonic acid | 7.09 | 4.87 | 4.65 | 3.63 | 5.53 | 3.55 | 3.57 | 4.54 | 4.88 | 6.02 | 6.37 | 4.13 | 3.95 | 6.78 | 4.12 | 5.97 |
| 27 | L-Valine | 0.02 | 0.02 | 0.02 | 0.02 | 0.01 | 0.01 | 0.02 | 0.02 | 0.02 | 0.02 | 0.02 | 0.02 | 0.02 | 0.04 | 0.02 | 0.02 |
| 28 | Palmitic acid | 0.19 | 0.09 | 0.10 | 0.14 | 0.17 | 0.10 | 0.11 | 0.19 | 0.52 | 0.16 | 0.21 | 0.17 | 0.13 | 0.29 | 0.24 | 0.19 |
| 29 | Citric acid | 0.75 | 0.64 | 0.54 | 0.63 | 0.46 | 0.61 | 0.57 | 0.60 | 0.35 | 0.36 | 0.61 | 0.60 | 0.35 | 1.09 | 0.58 | 0.55 |
| 30 | LysoPE (18:1(9Z)/0:0) | 0.02 | 0.02 | 0.02 | 0.02 | 0.02 | 0.02 | 0.02 | 0.01 | 0.02 | 0.02 | 0.02 | 0.02 | 0.02 | 0.04 | 0.02 | 0.02 |
| ID | MS2 name | DCF1 | DCF2 | DCF3 | DCF4 | DCF5 | DCF6 | DCF7 | DCF8 | NCF1 | NCF2 | NCF3 | NCF4 | NCF5 | NCF6 | NCF7 | NCF8 |
| 31 | Glycerol 3-phosphate | 0.27 | 0.51 | 0.17 | 0.20 | 0.21 | 0.12 | 0.19 | 0.19 | 0.20 | 0.12 | 0.37 | 0.13 | 0.23 | 0.02 | 0.15 | 0.34 |
| 32 | L-Serine | 0.03 | 0.03 | 0.02 | 0.00 | 0.02 | 0.02 | 0.00 | 0.01 | 0.02 | 0.03 | 0.02 | 0.03 | 0.02 | 0.01 | 0.03 | 0.01 |
| 33 | Bovinic acid | 1.88 | 1.81 | 1.87 | 1.86 | 2.07 | 1.26 | 1.81 | 1.97 | 0.10 | 1.59 | 2.59 | 1.77 | 1.70 | 1.25 | 1.62 | 2.61 |
| 34 | cis-Vaccenic acid | 0.43 | 0.52 | 0.33 | 0.28 | 0.31 | 0.39 | 0.31 | 0.24 | 0.25 | 0.27 | 0.35 | 0.38 | 0.32 | 0.62 | 0.29 | 0.28 |
| 35 | Indoleacetaldehyde | 0.05 | 0.07 | 0.04 | 0.06 | 0.05 | 0.05 | 0.05 | 0.07 | 0.06 | 0.07 | 0.06 | 0.06 | 0.08 | 0.07 | 0.07 | 0.06 |
| 36 | D-Ribose 5-phosphate | 0.05 | 0.07 | 0.05 | 0.04 | 0.04 | 0.13 | 0.06 | 0.02 | 0.05 | 0.04 | 0.14 | 0.10 | 0.06 | 0.12 | 0.12 | 0.16 |
| 37 | Adenosine monophosphate | 0.01 | 0.07 | 0.07 | 0.02 | 0.07 | 0.04 | 0.02 | 0.04 | 0.05 | 0.10 | 0.09 | 0.10 | 0.15 | 0.04 | 0.04 | 0.07 |
| 38 | Pyrophosphate | 0.10 | 0.06 | 0.04 | 0.04 | 0.07 | 0.04 | 0.04 | 0.06 | 0.06 | 0.06 | 0.12 | 0.05 | 0.05 | 0.05 | 0.06 | 0.11 |
| 39 | Glucose 6-phosphate | 0.49 | 0.43 | 0.42 | 0.40 | 0.35 | 0.35 | 0.37 | 0.42 | 0.33 | 0.39 | 0.42 | 0.37 | 0.19 | 0.75 | 0.39 | 0.37 |
| 40 | Inosine | 7.43 | 11.63 | 6.99 | 6.86 | 9.98 | 4.61 | 6.60 | 10.41 | 8.09 | 5.25 | 7.36 | 5.13 | 4.51 | 3.15 | 4.68 | 7.38 |
| 41 | ADP | 0.51 | 0.34 | 0.29 | 0.28 | 0.51 | 0.20 | 0.25 | 0.43 | 0.35 | 0.30 | 0.37 | 0.21 | 0.19 | 0.42 | 0.25 | 0.34 |
| 42 | Pantothenic acid | 0.01 | 0.02 | 0.01 | 0.00 | 0.04 | 0.00 | 0.00 | 0.00 | 0.00 | 0.00 | 0.00 | 0.00 | 0.00 | 0.00 | 0.00 | 0.00 |
| 43 | Phytanic acid | 0.04 | 0.05 | 0.05 | 0.07 | 0.04 | 0.06 | 0.06 | 0.05 | 0.04 | 0.05 | 0.04 | 0.04 | 0.05 | 0.11 | 0.04 | 0.04 |
| 44 | L-Phenylalanine | 0.03 | 0.07 | 0.03 | 0.03 | 0.04 | 0.05 | 0.03 | 0.02 | 0.02 | 0.04 | 0.03 | 0.03 | 0.03 | 0.04 | 0.04 | 0.02 |
| 45 | Caproic acid | 0.15 | 0.12 | 0.11 | 0.17 | 0.16 | 0.15 | 0.16 | 0.10 | 0.09 | 0.16 | 0.16 | 0.13 | 0.08 | 0.23 | 0.09 | 0.15 |
| 46 | gamma-Aminobutyric acid | 0.04 | 0.03 | 0.02 | 0.03 | 0.02 | 0.02 | 0.02 | 0.03 | 0.02 | 0.02 | 0.02 | 0.02 | 0.02 | 0.04 | 0.03 | 0.02 |
| 47 | Adrenic acid | 0.47 | 0.17 | 0.28 | 0.32 | 0.45 | 0.23 | 0.30 | 0.38 | 0.31 | 0.29 | 0.45 | 0.25 | 0.29 | 0.75 | 0.33 | 0.44 |
| 48 | Uracil | 0.02 | 0.04 | 0.02 | 0.03 | 0.02 | 0.02 | 0.02 | 0.02 | 0.06 | 0.03 | 0.05 | 0.05 | 0.03 | 0.04 | 0.05 | 0.04 |
| 49 | Guanosine monophosphate | 0.24 | 0.26 | 0.12 | 0.07 | 0.18 | 0.08 | 0.06 | 0.15 | 0.20 | 0.16 | 0.14 | 0.13 | 0.11 | 0.05 | 0.11 | 0.11 |
| 50 | Sedoheptulose | 1.72 | 1.49 | 0.83 | 0.91 | 1.55 | 0.84 | 0.82 | 1.56 | 1.41 | 1.36 | 2.45 | 2.91 | 2.50 | 2.46 | 1.56 | 2.28 |
| 51 | Oxypurinol | 0.76 | 1.03 | 0.54 | 0.76 | 0.87 | 0.53 | 0.68 | 0.67 | 0.69 | 0.49 | 0.75 | 0.46 | 0.70 | 0.67 | 0.56 | 0.73 |
| 52 | Caprylic acid | 0.18 | 0.18 | 0.16 | 0.17 | 0.19 | 0.16 | 0.18 | 0.17 | 0.21 | 0.28 | 0.29 | 0.27 | 0.20 | 0.28 | 0.30 | 0.29 |
| 53 | 5Z-Dodecenoic acid | 0.01 | 0.02 | 0.01 | 0.01 | 0.01 | 0.01 | 0.01 | 0.01 | 0.02 | 0.01 | 0.02 | 0.02 | 0.01 | 0.02 | 0.02 | 0.01 |
| 54 | Eicosadienoic acid | 0.09 | 0.13 | 0.40 | 0.11 | 0.29 | 0.10 | 0.11 | 0.17 | 0.16 | 0.07 | 0.16 | 0.11 | 0.18 | 0.06 | 0.13 | 0.14 |
| 55 | Nonadecanoic acid | 0.14 | 0.40 | 0.11 | 0.15 | 0.18 | 0.10 | 0.14 | 0.38 | 0.59 | 0.34 | 0.59 | 0.72 | 0.60 | 0.54 | 0.46 | 0.47 |
| 56 | N-Acetyl-L-aspartic acid | 2.25 | 4.15 | 1.32 | 2.76 | 2.96 | 1.93 | 2.53 | 1.53 | 2.76 | 1.87 | 3.47 | 2.92 | 2.80 | 4.12 | 3.24 | 3.34 |
| 57 | Docosapentaenoic acid (22n-3) | 0.24 | 1.09 | 0.14 | 0.64 | 0.26 | 1.04 | 0.84 | 0.49 | 0.04 | 0.44 | 0.15 | 0.13 | 1.01 | 0.00 | 0.41 | 0.17 |
| 58 | Malonic acid | 1.85 | 1.76 | 1.21 | 1.35 | 1.72 | 0.94 | 1.37 | 2.20 | 1.10 | 1.14 | 1.38 | 0.88 | 0.91 | 0.80 | 0.96 | 1.39 |
| 59 | N-Acetyl-glucosamine 1-phosphate | 0.03 | 0.04 | 0.04 | 0.02 | 0.10 | 0.02 | 0.02 | 0.02 | 0.00 | 0.00 | 0.00 | 0.00 | 0.00 | 0.00 | 0.00 | 0.00 |
| 60 | Heptadecanoic acid | 1.09 | 1.04 | 0.67 | 0.66 | 1.19 | 0.50 | 0.59 | 0.77 | 0.73 | 0.56 | 0.97 | 0.53 | 0.59 | 0.75 | 0.64 | 0.93 |
| ID | MS2 name | DCF1 | DCF2 | DCF3 | DCF4 | DCF5 | DCF6 | DCF7 | DCF8 | NCF1 | NCF2 | NCF3 | NCF4 | NCF5 | NCF6 | NCF7 | NCF8 |
| 61 | (Â±)-Tryptophan | 5.53 | 3.71 | 1.81 | 2.40 | 3.53 | 2.25 | 2.21 | 3.28 | 3.80 | 3.40 | 6.33 | 6.20 | 2.41 | 3.16 | 4.27 | 5.71 |
| 62 | N-Acetylornithine | 0.31 | 0.28 | 0.21 | 0.24 | 0.16 | 0.27 | 0.24 | 0.08 | 0.16 | 0.13 | 0.27 | 0.10 | 0.06 | 0.37 | 0.26 | 0.27 |
| 63 | Glycocholic acid | 0.03 | 0.02 | 0.02 | 0.02 | 0.03 | 0.02 | 0.02 | 0.02 | 0.03 | 0.02 | 0.03 | 0.02 | 0.02 | 0.02 | 0.02 | 0.03 |
| 64 | Mannitol | 0.02 | 0.03 | 0.02 | 0.02 | 0.02 | 0.02 | 0.02 | 0.02 | 0.01 | 0.01 | 0.01 | 0.01 | 0.03 | 0.01 | 0.01 | 0.01 |
| 65 | Uridine diphosphategalactose | 0.36 | 0.72 | 1.60 | 0.36 | 0.66 | 1.40 | 1.38 | 0.86 | 1.90 | 1.95 | 1.12 | 1.16 | 0.95 | 2.26 | 0.64 | 2.99 |
| 66 | Aminoadipic acid | 1.05 | 0.99 | 0.12 | 0.18 | 0.57 | 0.47 | 0.45 | 0.80 | 0.86 | 1.04 | 1.35 | 0.86 | 0.88 | 1.00 | 0.55 | 1.27 |
| 67 | Linoleic acid | 0.01 | 0.10 | 0.08 | 0.02 | 0.10 | 0.06 | 0.02 | 0.05 | 0.07 | 0.15 | 0.11 | 0.17 | 0.48 | 0.05 | 0.05 | 0.04 |
| 68 | 13S-hydroxyoctadecadienoic acid | 0.25 | 0.28 | 0.32 | 0.11 | 0.21 | 0.19 | 0.10 | 0.16 | 0.18 | 0.15 | 0.16 | 0.13 | 0.13 | 0.06 | 0.11 | 0.14 |
| 69 | D-Xylitol | 0.05 | 0.03 | 0.04 | 0.04 | 0.04 | 0.03 | 0.03 | 0.03 | 0.05 | 0.03 | 0.03 | 0.03 | 0.03 | 0.06 | 0.03 | 0.03 |
| 70 | L-Histidine | 0.12 | 0.15 | 0.07 | 0.07 | 0.09 | 0.04 | 0.06 | 0.06 | 0.08 | 0.04 | 0.10 | 0.03 | 0.05 | 0.00 | 0.04 | 0.07 |
| 71 | 9,10-epoxyoctadecanoic acid | 0.49 | 0.67 | 0.85 | 0.77 | 1.07 | 0.53 | 0.75 | 0.90 | 0.65 | 0.55 | 0.44 | 0.43 | 0.38 | 0.75 | 0.51 | 0.46 |
| 72 | L-Tyrosine | 0.23 | 0.42 | 0.25 | 0.16 | 0.25 | 0.15 | 0.14 | 0.28 | 0.51 | 0.29 | 0.32 | 0.24 | 0.22 | 0.10 | 0.27 | 0.27 |
| 73 | Glutathione | 0.01 | 0.01 | 0.01 | 0.00 | 0.01 | 0.00 | 0.00 | 0.01 | 0.01 | 0.01 | 0.01 | 0.00 | 0.00 | 0.00 | 0.00 | 0.01 |
| 74 | Acetylcysteine | 0.10 | 0.25 | 0.05 | 0.08 | 0.06 | 0.07 | 0.06 | 0.06 | 0.17 | 0.08 | 0.08 | 0.05 | 0.07 | 0.15 | 0.06 | 0.07 |
| 75 | Uridine | 0.14 | 0.06 | 0.09 | 0.05 | 0.08 | 0.08 | 0.07 | 0.07 | 0.09 | 0.15 | 0.09 | 0.08 | 0.09 | 0.16 | 0.07 | 0.08 |
| 76 | Creatinine | 0.03 | 0.05 | 0.05 | 0.03 | 0.04 | 0.02 | 0.02 | 0.05 | 0.06 | 0.04 | 0.04 | 0.04 | 0.03 | 0.03 | 0.07 | 0.03 |
| 77 | 8,11,14-Eicosatrienoic acid | 0.05 | 0.04 | 0.04 | 0.04 | 0.04 | 0.04 | 0.04 | 0.04 | 0.04 | 0.04 | 0.05 | 0.04 | 0.04 | 0.09 | 0.04 | 0.04 |
| 78 | (9xi,10xi,12xi)-9,10-Dihydroxy-12-octadecenoic acid | 0.60 | 1.03 | 0.68 | 0.37 | 0.67 | 0.50 | 0.32 | 0.64 | 1.16 | 0.91 | 0.83 | 0.58 | 0.69 | 0.23 | 0.61 | 0.70 |
| 79 | N2-gamma-Glutamylglutamine | 0.00 | 0.01 | 0.00 | 0.02 | 0.00 | 0.04 | 0.02 | 0.00 | 0.00 | 0.00 | 0.00 | 0.00 | 0.04 | 0.02 | 0.01 | 0.00 |
| 80 | Uridine diphosphate glucuronic acid | 0.53 | 0.20 | 0.46 | 0.17 | 0.55 | 0.13 | 0.19 | 0.35 | 0.35 | 0.23 | 0.39 | 0.27 | 0.16 | 0.08 | 0.23 | 0.38 |
| 81 | Oxidized glutathione | 0.00 | 0.01 | 0.00 | 0.00 | 0.00 | 0.00 | 0.00 | 0.01 | 0.01 | 0.01 | 0.01 | 0.01 | 0.02 | 0.00 | 0.00 | 0.01 |
| 82 | Docosatrienoic acid | 0.14 | 0.09 | 0.06 | 0.05 | 0.05 | 0.05 | 0.05 | 0.05 | 0.07 | 0.08 | 0.06 | 0.06 | 0.06 | 0.11 | 0.05 | 0.05 |
| 83 | Docosahexaenoic acid | 0.01 | 0.08 | 0.02 | 0.03 | 0.01 | 0.16 | 0.04 | 0.03 | 0.01 | 0.03 | 0.03 | 0.02 | 0.09 | 0.40 | 0.03 | 0.03 |
| 84 | Eicosapentaenoic acid | 0.45 | 0.09 | 0.16 | 0.16 | 0.29 | 0.09 | 0.15 | 0.29 | 0.18 | 0.21 | 0.38 | 0.19 | 0.11 | 0.09 | 0.16 | 0.39 |
| 85 | p-Cresol sulfate | 0.10 | 0.16 | 0.16 | 0.08 | 0.36 | 0.08 | 0.07 | 0.06 | 0.00 | 0.00 | 0.00 | 0.00 | 0.00 | 0.00 | 0.00 | 0.00 |
| 86 | Tauroursodeoxycholic acid | 0.11 | 0.03 | 0.05 | 0.13 | 0.03 | 0.14 | 0.19 | 0.03 | 0.01 | 0.03 | 0.19 | 0.12 | 0.13 | 0.07 | 0.03 | 0.23 |
| 87 | NAD | 0.19 | 0.31 | 0.18 | 0.28 | 0.22 | 0.23 | 0.25 | 0.34 | 0.23 | 0.35 | 0.35 | 0.30 | 0.27 | 0.49 | 0.20 | 0.31 |
| 88 | Glutamylglutamic acid | 0.02 | 0.03 | 0.08 | 0.02 | 0.06 | 0.02 | 0.02 | 0.03 | 0.03 | 0.01 | 0.03 | 0.02 | 0.03 | 0.01 | 0.03 | 0.03 |
| 89 | 4-Dodecylbenzenesulfonic Acid | 0.68 | 0.42 | 0.61 | 0.58 | 0.54 | 0.39 | 0.53 | 0.45 | 0.50 | 0.42 | 0.72 | 0.63 | 0.40 | 1.11 | 0.59 | 0.37 |
| ID | MS2 name | DCF1 | DCF2 | DCF3 | DCF4 | DCF5 | DCF6 | DCF7 | DCF8 | NCF1 | NCF2 | NCF3 | NCF4 | NCF5 | NCF6 | NCF7 | NCF8 |
| 90 | Trehalose | 1.44 | 1.38 | 0.78 | 0.97 | 1.19 | 0.92 | 0.92 | 1.00 | 0.83 | 0.96 | 0.99 | 0.75 | 0.82 | 1.12 | 0.75 | 0.97 |
| 91 | Raffinose | 0.01 | 0.01 | 0.01 | 0.00 | 0.02 | 0.01 | 0.00 | 0.00 | 0.01 | 0.00 | 0.00 | 0.00 | 0.02 | 0.00 | 0.01 | 0.00 |
| 92 | Taurocholic acid | 0.15 | 0.20 | 0.29 | 0.15 | 0.26 | 0.16 | 0.15 | 0.28 | 0.44 | 0.31 | 0.49 | 0.78 | 0.85 | 0.31 | 0.29 | 0.46 |
| 93 | 3-Hydroxyvaleric acid | 0.14 | 0.20 | 0.23 | 0.14 | 0.23 | 0.13 | 0.13 | 0.24 | 0.33 | 0.23 | 0.33 | 0.44 | 0.40 | 0.21 | 0.32 | 0.32 |
| 94 | L-Glutamine | 0.84 | 0.84 | 0.50 | 0.48 | 0.96 | 0.40 | 0.43 | 3.55 | 1.20 | 0.51 | 0.69 | 0.36 | 0.39 | 0.31 | 0.54 | 1.57 |
| 95 | Succinic acid | 0.02 | 0.02 | 0.02 | 0.01 | 0.01 | 0.01 | 0.00 | 0.02 | 0.08 | 0.04 | 0.04 | 0.03 | 0.04 | 0.01 | 0.03 | 0.03 |
| 96 | Stearidonic acid | 0.23 | 0.21 | 1.16 | 0.95 | 0.23 | 1.05 | 0.94 | 1.16 | 1.16 | 0.94 | 2.20 | 0.58 | 0.75 | 0.56 | 0.68 | 1.29 |
| 97 | myo-Inositol | 0.01 | 0.01 | 0.02 | 0.01 | 0.01 | 0.01 | 0.01 | 0.01 | 0.01 | 0.02 | 0.01 | 0.01 | 0.01 | 0.02 | 0.01 | 0.01 |
| 98 | (Â±)-erythro-Isoleucine | 0.01 | 0.00 | 0.00 | 0.01 | 0.05 | 0.08 | 0.09 | 0.08 | 0.01 | 0.00 | 0.00 | 0.01 | 0.08 | 0.11 | 0.09 | 0.10 |
| 99 | N-Acetylneuraminic acid | 0.02 | 0.02 | 0.02 | 0.02 | 0.02 | 0.01 | 0.01 | 0.01 | 0.02 | 0.02 | 0.02 | 0.02 | 0.01 | 0.02 | 0.01 | 0.02 |
| 100 | Threonic acid | 0.06 | 0.01 | 0.04 | 0.01 | 0.04 | 0.01 | 0.01 | 0.03 | 0.05 | 0.04 | 0.06 | 0.04 | 0.02 | 0.02 | 0.03 | 0.05 |
| 101 | L-Glutamic acid | 0.05 | 0.05 | 0.01 | 0.04 | 0.03 | 0.02 | 0.04 | 0.05 | 0.02 | 0.02 | 0.05 | 0.02 | 0.03 | 0.02 | 0.01 | 0.04 |
| 102 | 4-Nitrophenol | 11.05 | 6.66 | 4.50 | 5.68 | 8.98 | 3.66 | 5.26 | 8.78 | 4.70 | 6.39 | 11.40 | 4.60 | 3.84 | 5.25 | 5.95 | 10.63 |
| 103 | L-Aspartic acid | 0.03 | 0.04 | 0.02 | 0.02 | 0.03 | 0.02 | 0.02 | 0.03 | 0.00 | 0.01 | 0.02 | 0.01 | 0.04 | 0.01 | 0.01 | 0.02 |
| 104 | 3-Hydroxybutyric acid | 0.01 | 0.01 | 0.01 | 0.00 | 0.02 | 0.00 | 0.00 | 0.00 | 0.02 | 0.01 | 0.01 | 0.02 | 0.00 | 0.00 | 0.01 | 0.01 |
| 105 | beta-D-Glucosamine | 0.17 | 0.04 | 0.12 | 0.04 | 0.14 | 0.03 | 0.04 | 0.08 | 0.09 | 0.08 | 0.08 | 0.08 | 0.05 | 0.03 | 0.08 | 0.07 |
| 106 | 12-HETE | 0.15 | 0.18 | 0.13 | 0.16 | 0.17 | 0.11 | 0.15 | 0.20 | 0.22 | 0.13 | 0.12 | 0.08 | 0.09 | 0.18 | 0.15 | 0.12 |
| 107 | Gluconic acid | 0.64 | 0.61 | 0.56 | 0.58 | 0.53 | 0.53 | 0.53 | 0.51 | 0.55 | 0.57 | 0.62 | 0.55 | 0.58 | 1.09 | 0.54 | 0.56 |

LC-MS = liquid chromatograph-mass spectrometer; NCF = normal colostrum feeding; DCF = delayed colostrum feeding.Table S4 KEGG enrichment analysis of upregulated DEGs.

| Pathway | Upregulation (699) | All (8243) | Adjusted *P*-value | Pathway ID | Genes |
| --- | --- | --- | --- | --- | --- |
| Chemokine signaling pathway | 47 | 184 | <0.001 | ko04062 | *RASGRP2, PLCB2, LOC102170310, CCL5, JAK3, PREX1, PRKCB, PIK3R5, PIK3CG, CCL26, CCL24, CCL21, NCF1, ITK, VAV2, VAV1, PIK3CD, DOCK2, FGR, RAC2, CXCL16, CCR5, XCR1, CXCR6, GRK2, CCR9, LOC102181582, CXCL14, GNG7, WAS, HCK, LYN, PTK2B, GRK3, ADCY7, STAT3, GRK6, PRKCZ, ARRB2, CCR6, CX3CR1, CXCL9, STAT1, STAT2, LOC102190717, LOC102190981, CCL25* |
| T cell receptor signaling pathway | 35 | 112 | <0.001 | ko04660 | *PRKCQ, FOS, LCK, PIK3R5, RASGRP1, PIK3CG, PTPRC, CD247, ITK, LOC102176695, VAV2, VAV1, PIK3CD, FYN, ICOS, NCK2, LCP2, CD8B, CD8A, CBL, JUN, GRAP2, CBLB, CBLC, NFATC1, MAP3K14, PTPN6, CARD11, CD3E, CD3D, CD3G, TRBC1, V-TCR, V-TCR, NFATC2* |
| Hematopoietic cell lineage | 31 | 100 | <0.001 | ko04640 | *CSF1R, KIT, IL2RA, LOC102174471, LOC102174924, CSF2RA, IL4R, CD14, CD5, IL6R, ITGAM, LOC102180271, CD2, CD44, ITGA4, IL11, CD8B, MME, CD8A, ANPEP, CD7, FLT3, CD3E, CD37, CD3D, CD3G, LOC108633303, CD24, Ighg1, IGHA2, HLA-DRB1* |
| Osteoclast differentiation | 35 | 132 | <0.001 | ko04380 | *CSF1R, LOC102171241, FOS, FOSL2, NCF2, LCK, PIK3R5, PIK3CG, SYK, SIRPA, TYROBP, NCF4, LOC102175938, BTK, NCF1, GAB2, PIK3CD, FYN, PLCG2, LCP2, JUN, LOC102185698, SPI1, NFATC1, TYK2, LOC102188617, STAT1, MAP3K14, IRF9, STAT2, TNFRSF1A, ACP5, LOC106501822, SOCS1, NFATC2* |
| Complement and coagulation cascades | 25 | 84 | <0.001 | ko04610 | *C3, F13A1, SERPINE1, CFHR5, CFD, KNG1, LOC102175560, CFB, C2, C4BPB, C4BPA, C6, C3AR1, SERPING1, CFI, LOC102185401, PLAUR, C1QB C1QC, C1QA, LOC102188588, C1R, CFH, C1S, LOC102189753* |
| Antigen processing and presentation | 26 | 93 | <0.001 | ko04612 | *HSP70.1, B2M, CIITA, LOC102178315, LOC102180664, TAP1, LOC102181202, CTSS, LOC102184229, CD8B, CD8A, HSPA6, CTSL, CD74, HSPA2, LOC102187356, LOC102187998, LOC102188267, LOC102188814, LOC102188986, LOC102189356, LOC108636059);MSTRG.15268(HLA-DRB1, TRBC1, V-TCR, V-TCR* |
| Intestinal immune network for IgA production | 20 | 59 | <0.001 | ko04672 | *TNFSF13B, ICOS, ITGB7, LOC102180664, CCR9, ITGA4, CD86, LOC102187356, LOC102187998, LOC102188267, LOC102188986, LOC102189356, MAP3K1, CCL25, Ighg1, IGHA2, HLA-DRB1, TRBC1, V-TCR, V-TCR* |
| B cell receptor signaling pathway | 23 | 77 | <0.001 | ko04662 | *INPP5D., FOS., PRKCB, PIK3R5, PIK3CG, SYK, BTK, VAV2, VAV1, PIK3CD, RAC2, DAPP1, INPPL1, PLCG2, LYN, JUN, NFATC1, PTPN6, CARD11, LOC106501751, Ighg1, IGHA2, NFATC2* |
| NF-kappa B signaling pathway | 27 | 102 | <0.001 | ko04064 | *TNFSF13B, PRKCQ, LCK, PRKCB, BCL2A1, SYK, MYD88, CCL21, BTK, LOC102176695, CD14, PLCG2, TRIM25, TRAF1, TNFAIP3, DDX58, LYN, TRAF5, MAP3K14, TNFRSF1A, CARD11, TNFSF14, Ighg1, IGHA2, TRBC1, V-TCR, V-TCR* |
| Cell adhesion molecules | 35 | 170 | <0.001 | ko04514 | *ITGB2, CD226, SPN, SELL, CLDN4, ITGAL, PTPRC, CD6, CLDN3, ICOS, SELPLG, ITGAM, TGB7, LOC102180664, PTPRF, LOC102181202, SIGLEC1, CD2, ITGA4, CD8B, CD86, CD8A, SDC4, LOC102187356, LOC102187998, LOC102188267, LOC102188814, LOC102188986, LOC102189356, ICAM3, LOC108638310, HLA-DRB1, TRBC1, V-TCR, V-TCR* |
| Natural killer cell mediated cytotoxicity | 38 | 193 | <0.001 | ko04650 | *ITGB2, PRF1, SH2D1A, LCK, PRKCB, ITGAL, PIK3R5, PIK3CG, TNFSF10, CD247, SYK, TYROBP, LOC102176695, VAV2, LOC102177285, VAV1, PIK3CD, CD244, RAC2, FYN, PLCG2, SH2D1B, LOC102181202, LOC102184229, LCP2, PTK2B, LOC102185698, NFATC1, LOC102188814, PTPN6, FCER1G, LOC108633178, CD48, LOC108636059, LOC108638310, Ighg1, IGHA2, NFATC2* |
| Fc gamma R-mediated phagocytosis | 24 | 96 | <0.001 | ko04666 | *ARPC1B, INPP5D, PRKCB, PIK3R5, PIK3CG, PTPRC, SYK, NCF1, VAV2, GAB2, VAV1, PIK3CD, DOCK2, RAC2, INPPL1, GSN, PLCG2, WAS, HCK, LYN, SCIN, WASF2, Ighg1, IGHA2* |
| Phagosome | 33 | 181 | <0.001 | ko04145 | *C3, ITGB2, COLEC12, NCF2, CD209, RAB7B, NCF4, LOC102175938, NCF1, CD14, TUBB4A, ACTB, ITGAM, LOC102180664, TAP1, LOC102181202, CTSS, COLEC11, CORO1A, CTSL, LOC102185698, LOC102187356, LOC102187998, LOC102188267, LOC102188617, LOC102188814, C1R, LOC102188986, LOC102189356, LOC108633303, Ighg1, IGHA2, HLA-DRB1* |
| Fc epsilon RI signaling pathway | 18 | 75 | <0.001 | ko04664 | *INPP5D, PIK3R5, PIK3CG, SYK, BTK, VAV2, GAB2, VAV1, PIK3CD, RAC2, FYN, PLCG2, LCP2, LYN, PLA2G4F, FCER1G, Ighg1, IGHA2* |
| Th1 and Th2 cell differentiation | 6 | 10 | <0.001 | ko04658 | *HLA-DRB1, TRBC1, V-TCR, V-TCR, IL2RB, NFATC2* |
| Th17 cell differentiation | 6 | 10 | <0.001 | ko04659 | *HLA-DRB1, TRBC1, V-TCR, V-TCR, IL2RB, NFATC2* |
| Leukocyte transendothelial migration | 27 | 149 | <0.001 | ko04670 | *ITGB2, CLDN4, NCF2, PRKCB, ITGAL, PIK3R5, PIK3CG, SIPA1, RHOH, RASSF5, NCF4, LOC102175938, NCF1, ITK, VAV2, VAV1, PIK3CD, RAC2, CLDN3, ITGAM, PLCG2, EZR, ITGA4, PTK2B, TXK, LOC102188617, MSTRG.3902(--)* |
| Arachidonic acid metabolism | 22 | 115 | 0.002 | ko00590 | *PLA2G2C, ALOX5, LOC102170006, PLA2G2A, PLB1, LOC102172133, CBR3, LOC102176622, LOC102181858, PLA2G6, LOC102182119, GGT5, LOC102183680, EPHX2, LOC102186455, LOC102186759 LOC102186942, PLA2G4F, LOC102188304, LOC102189109, LOC106503208, pol* |
| Rap1 signaling pathway | 34 | 223 | 0.003 | ko04015 | *VEGFA, ITGB2, CSF1R, KIT, EFNA1, RASGRP2, PLCB2, PRKCB, ITGAL, PIK3R5, PIK3CG, SIPA1, APBB1IP, RASSF5, PIK3CD, SIPA1L3, RAC2, TLN1, ITGAM, ANGPT4, ID1, LCP2, EPHA2, ADCY7, RGS14, PRKCZ, LOC102187310, LPAR5, FARP2, FYB, TRBC1, V-TCR, V-TCR, MSTRG.4722(--)* |
| Platelet activation | 22 | 125 | 0.005 | ko04611 | *RASGRP2, PLCB2, ITPR2, PIK3R5, RASGRP1, PIK3CG, SYK, APBB1IP, BTK, PIK3CD, TLN1, FYN, PLCG2, LCP2, LYN, ITPR3, ADCY7, PRKCZ, PLA2G4F, FERMT3, FCER1G, MYLK* |
| Phosphatidylinositol signaling system | 19 | 103 | 0.006 | ko04070 | *PIP4K2A, PLCB2, ITPR2, INPP5D, DGKD, PRKCB, PIK3R5, PIK3CG, DGKA, PIK3CD, IMPA2, INPPL1, PLCG2, INPP4A, ITPK1, ITPR3, IP6K2, ITPKC, SYNJ2* |
| Ras signaling pathway | 35 | 244 | 0.008 | ko04014 | *VEGFA, CSF1R, KIT, PLA2G2C, EFNA1, RASGRP2, REL, LOC102170006, PLA2G2A, FOXO4, RASSF1, PRKCB, PIK3R5, RASGRP1, PIK3CG, RASSF5, LOC102176695, GAB2, PIK3CD, RAC2, PLCG2, ANGPT4, PLA2G6, GNG7, SYNGAP1, STK4, EPHA2, LOC102187310, RASAL3, PLA2G4F, LOC102189340, TRBC1, V-TCR, V-TCR, MSTRG.4722(--)* |
| Regulation of actin cytoskeleton | 32 | 223 | 0.012 | ko04810 | *ITGB2, PIP4K2A, ARPC1B, ITGAL, ARHGEF6, PIK3R5, PIK3CG, VAV2, CD14, CYFIP2, VAV1, PIK3CD, RAC2, ITGAE, ACTB, ITGAM, ITGB7, GSN, ITGAX, EZR, ITGA4, WAS, BAIAP2, NCKAP1L, SCIN, LOC102187310, FGD3, TMSB4X, SSH1, WASF2, MYLK, MSTRG.4722(--)* |
| Carbohydrate digestion and absorption | 11 | 49 | 0.012 | ko04973 | *LCT, PRKCB, PIK3R5, PIK3CG, PIK3CD, MGAM, HK3, SI, ATP1B3, HKDC1, SI* |
| Cytokine-cytokine receptor interaction | 42 | 318 | 0.013 | ko04060 | *TNFSF13B, VEGFA, IL18, CSF1R, KIT, IL2RG, IL2RA, LOC102170310, CCL5, TNFSF10, CCL26, CCL24, LOC102174924, CSF2RA, CCL21, IL4R, CSF2RB, IL21R, TNFRSF9, CXCL16, IL22RA1, CCR5, TNFSF8, IL6R, XCR1, CXCR6, CCR9, LOC102181582, CXCL14, IFNLR1, IL11, CCR6, CX3CR1, CXCL9, IL10RA, FLT3, TNFRSF1A, LOC102190717, LOC102190981, TNFSF14, CCL25, IL2RB* |
| Alpha-Linolenic acid metabolism | 8 | 31 | 0.018 | ko00592 | *PLA2G2C, LOC102170006, PLA2G2A, PLB1, ACOX3, PLA2G6, ACAA1, PLA2G4F* |
| PPAR signaling pathway | 15 | 83 | 0.020 | ko03320 | *NR1H3, PCK1, SLC27A5, SLC27A2, LOC102177672, ACOX3, LOC102179380, APOA1, ACOX2, ACAA1, SLC27A4, APOA2, AQP7, PLTP, LOC108633303* |
| AMPK signaling pathway | 20 | 128 | 0.027 | ko04152 | *LIPE, EEF2K, PCK1, PRKAB2, PFKFB3, PIK3R5, PIK3CG, ADIPOR2, CREB3L3, PIK3CD, CRTC, PPP2CB, CREB3L1, FOXO1, CAB39L, CAMKK1, PFKFB4, LOC108633303, IRS2, LOC108637886)* |
| Endocytosis | 37 | 287 | 0.032 | ko04144 | *HSP70.1, CSF1R, KIT, EHD1, IL2RG, IL2RA, ARPC1B, PML, LOC102174322, FOLR2, VPS37B, LOC102178315, CYTH4, CCR5, RAB11FIP1, ARAP1, GRK2, LOC102181202, ACAP1, WAS, PSD4, GRK3, CBL, HSPA6, GRK6, PRKCZ, ARRB2, HSPA2, CBLB, LOC102187310, AGAP2, CBLC, MVB12B, LOC102188814, MSTRG.17596(--), IL2RB, MSTRG.4722(--)* |
| Toll-like receptor signaling pathway | 18 | 114 | 0.035 | ko04620 | *SPP1, TLR5, TLR8, TLR1, CCL5, FOS, IRF3, PIK3R5, PIK3CG, MYD88, CD14, IRF5, PIK3CD, CD86, JUN, CXCL9, STAT1, IRF7* |
| Peroxisome | 17 | 107 | 0.040 | ko04146 | *ECI, PXMP2, HSD17B4, BAAT, SLC27A2, ACOX3, PHYH, ABCD1, ACOX2, ACAA1, PAOX, EPHX, MSTRG.13787(--), MSTRG.4225(--), MSTRG.4724(--), MSTRG.5431(--), MSTRG.6760(--)* |
| Primary bile acid biosynthesis | 5 | 16 | 0.041 | ko00120 | *HSD17B4, SLC27A5, BAAT, LOC102177672, ACOX2* |

KEGG = the Kyoto Encyclopedia of Genes and Genomes; DEGs = differentially expressed genes; PPAR = peroxisome proliferator-activated receptor; AMPK = adenosine 5’-monophosphate (AMP)-activated protein kinase.

Table S5 KEGG enrichment analysis of downregulated DEGs.

| Pathway | Downregulation (875) | All (8243) | Adjusted *P*-value | Pathway ID | Genes |
| --- | --- | --- | --- | --- | --- |
| Oxidative phosphorylation | 76 | 144 | 0.000 | ko00190 | *NDUFS7, SDHD, LOC100861263, NDUFA4, NDUFB8, NDUFS1, NDUFB3, ATP5G1, NDUFB10, LOC102168533, ATP6V1D, LOC102169976, NDUFS5, ATP5J2, ATP5O, NDUFS4, ATP6V0E2, NDUFAB1, LOC102170877, LOC102171149, NDUFB7, ATP5C1, LHPP, NDUFA3, LOC102172289, LOC102172376, PPA2, LOC102173562, ATP5H, LOC102175794, LOC102176290, NDUFV2, ATP6V1F, ATP6V1G1, NDUFA2, NDUFA1, NDUFB4, ATP6V0D1, ATP5F1, LOC102179036, LOC102179291, NDUFA9, ATP5G3, NDUFA12, NDUFA11, NDUFA10, NDUFC2, NDUFB6, LOC102183616, NDUFV1, ATP5A1, ATP6V1A, NDUFC1, LOC102186286, ATP5B, NDUFA13, LOC102187154, LOC102187496, NDUFA5, ATP5J, LOC102188021, NDUFS3, LOC102188264, LOC102188675, ATP5D, NDUFB5, NDUFB1, ATP5I, LOC102191741, ATP6V0C, LOC108635087, LOC108637473, LOC108638252, COX2, ND5, ND6* |
| Proteasome | 29 | 44 | 0.000 | ko03050 | *PSMA6, PSMA3, PSMA7, PSMD7, PSMB5, PSMC5, PSMD3, PSMD8, PSMB6, PSMB4, PSMD6, PSMC6, PSMD13, PSMB2, PSMB1, PSMB7, PSMD2, PSMD12, PSMA4, PSMD14, PSMA5, PSMC3, PSMD1, POMP, PSMA1, PSMC1, SHFM1, PSMC2, PSME3* |
| RNA transport | 60 | 181 | 0.000 | ko03013 | *EIF2S1, CLNS1A, THOC1, THOC7, EIF3B, LOC102169858, NUP155, NUP85, EIF5, KPNB1, ELAC1, GEMIN5, NUP107, SUMO2, EIF4A3, NUP37, ELAC2, PYM1, RAN, AAAS, RPP40, NCBP2, EIF2S2, GEMIN6, EIF2B4, POP1, NDC1, XPO5, SEH1L, MAGOH, EIF4E2, EIF2B1, MAGOHB, XPOT, RPP30, LOC102182187, DDX20, NUP133, POP4, EIF4G1, EIF4E, NCBP1, EIF1AX, EIF3J, RPP14, EIF2B3, NUP160, NUP43, NUP35, EIF3I, NUP93, SNUPN, SUMO3, TRNT1, EIF4A1, UBE2I, NUP205, LOC108635156, STRAP, MSTRG.383(--)* |
| Spliceosome | 47 | 142 | 0.000 | ko03040 | *SMNDC1, LSM2, SNRPE, THOC1, SNRPB2, DHX15, SF3B3, WBP11, LSM5, BUD31, SNRNP40, PQBP1, SF3B6, SF3A3, RBMX, SNRPB, PRPF40B, EIF4A3, LSM6, U2SURP, PPIH, NCBP2, HNRNPA3, SNRPD3, LSM7, SRSF10, MAGOH, SRSF3, MAGOHB, CWC15, CTNNBL1, SNRPA1, SNRPF, PLRG1, NCBP1, EFTUD2, PRPF38A, HNRNPA1, PRPF19, SNRPA, PHF5A, SNRPG, LSM3, SNRPD1, LSM4, LOC106503022, SNRPC* |
| Protein export | 13 | 23 | 0.000 | ko03060 | *SEC11C, SEC61G, SPCS1, SPCS3, SEC61A2, SEC11A, SRP14, SRP72, OXA1L, SEC62, SRP19, IMMP2L, SRP9* |
| Citrate cycle (TCA cycle) | 15 | 31 | 0.000 | ko00020 | *SDHD, PDHA1, IDH3B, MDH2, DLAT, DLD, MDH1, IDH3G, IDH3A, SUCLA2, ACO1, PDHB, FH, IDH1, ACO2* |
| Carbon metabolism | 33 | 120 | 0.000 | ko01200 | *ME1, GAPDH, SDHD, GOT1, PDHA1, PGAM1, IDH3B, LOC102173923, MDH2, ENO1, DLAT, DLD, MDH1PKM, CPS1, SHMT2, IDH3G, IDH3A, ESD, MTHFR SUCLA2, ACO1, PDHB, PGK1, LOC102186197, AGXT, RGN, FH, HADHA, GPI, IDH1, ACO2, HK1* |
| Aminoacyl-tRNA biosynthesis | 18 | 46 | 0.000 | ko00970 | *FARSB, QRSL1, LAR, FARS2, SEPSECS, DARS, VARS2, YARS, EPRS, DARS2, IARS2, RARS2, VARS, TARS, NARS2, LARS2, AARS2, RARS* |
| Ribosome biogenesis in eukaryotes | 25 | 83 | 0.000 | ko03008 | *NAT10, TBL3, PWP2, RBM28, UTP18, RAN, RPP40, MPHOSPH10, POP1, SPATA5, GNL3, REXO2, CSNK2B, RPP30, WDR75, POP4, DKC1, FBL, AK6, RIOK2, NOP56, NOP10, NHP2, NOL6, FCF1* |
| Cardiac muscle contraction | 25 | 84 | 0.000 | ko04260 | *TNNI3, LOC100861263, ATP1B1, LOC102168533, LOC102169976, LOC102170877, LOC102171149, LOC102172289, LOC102172376, LOC102173562, LOC102176290, LOC102179036, LOC102179291, ATP1A1, LOC102183616, LOC102186286, LOC102187154, LOC102187496, LOC102188021, LOC102188264, LOC102188675, LOC102191741, LOC108635087, LOC108637473, COX2* |
| RNA polymerase | 13 | 30 | 0.000 | ko03020 | *POLR1C, POLR2J, POLR2G, POLR3K, POLR2D, POLR2K, POLR2F, POLR2B, POLR3B, POLR2E, POLR1E, POLR1A, POLR3H* |
| Ribosome | 43 | 211 | 0.000 | ko03010 | *RPL21, MRPS10, MRPL1, MRPL16, MRPL19, MRPL28, MRPL12, MRPS7, RPS24, RPS25, RPS27L, MRPL2, MRPL24, MRPL22, MRPL34, RPS19, RPS17, MRPS15, MRPL11, MRPL23, MRPL17, MRPL13, MRPS12, RPL, MRPL4, MRPS14, MRPL27, MRPL30, RPS6, LOC102184991, RPL24, MRPL35, MRPL15, RPS27A, MRPL21, MRPS21, MRPL20, RPL26L1, RSL24D1, MRPS9, LOC108638514, Rps28, RPS20* |
| Steroid biosynthesis | 10 | 21 | 0.000 | ko00100 | *DHCR7, DHCR24, NSDHL, LSS, EBP, HSD17B7, MSMO1, SC5D, SQLE, EBP* |
| Pyruvate metabolism | 14 | 41 | 0.001 | ko00620 | *ME1, ACACA, PDHA1, MDH2, DLAT, DLD, MDH1, PKM, PDHB, GLO1, ACYP2, ALDH7A1, LDHB, FH* |
| Nucleotide excision repair | 18 | 62 | 0.001 | ko03420 | *GTF2H, RPA3, PCNA, CETN2, POLE3, CCNH, RFC4, RAD23A, MNAT1, RFC3, GTF2H3, RPA2, RFC5, LOC102185505, POLE4, GTF2H1, MSTRG.10886(--), MSTRG.6569(--)* |
| Purine metabolism | 37 | 183 | 0.001 | ko00230 | *POLR1C, POLR2J, POLR2G, POLR3K, HDDC3, POLE3, GMPR, LOC102173923, POLR2D, NUDT5, ENTPD6, PGM2, LOC102175795, NME1, POLR2K, GMPS, PKM, POLR2F, NT5C3B, POLR2B, AK2, ATIC, LOC102182773, PGM1, GUK1, IMPDH2, POLR3B, AK6, ADSL, POLR2E, POLR1E, ADK, POLR1A, POLR3H, ENPP1, NTPCR, POLE4* |
| Biosynthesis of amino acids | 20 | 78 | 0.002 | ko01230 | *GAPDH, OTC, GOT1, PGAM1, IDH3B, LOC102173923, ENO1, PKM, CPS1, SHMT2, IDH3G, IDH3A, ACO1, PGK1, ALDH7A1, IDH1, AADAT, ACO2, PYCR1, OTC* |
| RNA degradation | 20 | 78 | 0.002 | ko03018 | *CNOT7, HSPD1, LSM2, LSM5, EXOSC1, EXOSC8, ENO1, LSM6, SKIV2L, DHX36, LSM7, TTC37, C1D, MPHOSPH6, WDR61, EXOSC7, HSPA9, DCPS, LSM3, LSM4* |
| Protein processing in endoplasmic reticulum | 35 | 175 | 0.002 | ko04141 | *PDIA3, DAD1, MAPK8, EIF2S1, SSR1, SAR1A, TRAM1, P4HB, UBE2E3, RNF5, SEC61G, CANX, SEC61A2, LMAN1, ERLEC1, DNAJB11, STT3B, RAD23A, VCP, NSFL1C, VIMP, HSP90B1, SEC62, SSR2, SSR3, DNAJC10, DERL2, PDIA6, STT3A, EIF2AK3, HSPBP1, DDOST, TXNDC5, LOC108635156, LOC108637357* |
| 2-Oxocarboxylic acid metabolism | 8 | 17 | 0.002 | ko01210 | *GOT1, IDH3B, IDH3G, IDH3A, ACO1, IDH1, AADAT, ACO2* |
| Ascorbate and aldarate metabolism | 13 | 41 | 0.002 | ko00053 | *ALDH7A1, RGN, LOC102190288, LOC106501841, LOC108633246, LOC108635023, LOC108635036, LOC108635272, LOC108635540, UGT2B18, UGT2B17, UGT2B18, UGT2B18* |
| Porphyrin and chlorophyll metabolism | 16 | 59 | 0.003 | ko00860 | *HMBS, LOC102172983, EPRS, FXN, UROD, LOC102190288, LOC106501841, LOC108633246, LOC108635023, LOC108635036, LOC108635272, LOC108635540, UGT2B18, UGT2B17, UGT2B18, UGT2B18* |
| Cell cycle | 27 | 130 | 0.005 | ko04110 | *CDK2, TP53, ANAPC13, ANAPC1, CDK4, PCNA, ANAPC5, SKP2, E2F5, CCNH, DBF4, ANAPC7, HDAC2, YWHAE, BUB3, ORC3, RBL1, TFDP2, CCNE1, CHEK1, SMC3, YWHAQ, STAG1, MAD2L1, ORC2, ORC5, LOC108636541* |
| Terpenoid backbone biosynthesis | 8 | 21 | 0.009 | ko00900 | *MVD, HMGCR, HMGCS1, MVK, ICMT, FDPS, FNTA, LOC102191533* |
| Drug metabolism - other enzymes | 15 | 60 | 0.012 | ko00983 | *TPMT, UMPS, GMPS, IMPDH2, LOC102190288, LOC106501841, LOC108633246, LOC108635023, LOC108635036, LOC108635272, LOC108635540, UGT2B18, UGT2B17, UGT2B18, UGT2B18* |
| Mismatch repair | 8 | 23 | 0.017 | ko03430 | *RPA3, PCNA, MSH2, RFC4, SSBP1, RFC3, RPA2, RFC5* |
| Pyrimidine metabolism | 21 | 102 | 0.019 | ko00240 | *POLR1C, POLR2J, POLR2G, POLR3K, UMPS, POLE3, POLR2D, ENTPD6, LOC102175795, NME1, POLR2K, POLR2F, CAD, NT5C3B, POLR2B, POLR3B, POLR2E, POLR1E, POLR1A, POLR3H, POLE4* |
| Starch and sucrose metabolism | 14 | 59 | 0.025 | ko00500 | *PGM2, UXS1, PGM1, GBE1, GPI, LOC102190288, HK1, ENPP1, LOC106501841, LOC108633246, LOC108635023, LOC108635036, LOC108635272, LOC108635540* |
| N-Glycan biosynthesis | 13 | 53 | 0.025 | ko00510 | *DAD1, DPM1, STT3B, ALG14, ALG10, ALG3, STT3A, ALG6, ALG5, DPM2, DDOST, LOC106503782, LOC108637357* |
| Glycolysis/gluconeogenesis | 16 | 72 | 0.026 | ko00010 | *GAPDH), PDHA1, PGAM1, PGM2, ENO1, DLAT, DLD, PKM, PGM1, PDHB, PGK1, LOC102186197, ALDH7A1, LDHB, GPI, HK1* |
| Glycosylphosphatidylinositol-anchor biosynthesis | 8 | 25 | 0.026 | ko00563 | *PIGF, PIGY, PIGU, PIGP, PIGK, PIGB, DPM2, PIGG* |
| DNA replication | 10 | 36 | 0.026 | ko03030 | *RNASEH2B, RPA3, PCNA, POLE3, RFC4, SSBP1, RFC3, RPA2, RFC5, POLE4* |
| Fatty acid metabolism | 13 | 54 | 0.027 | ko01212 | *SCD, ACACA, FADS2, ACSL5, ACADSB, CPT2, ELOVL6, ACADVL, ACSL3, HSD17B12, TECR, HADHB, HADHA* |
| Amino sugar and nucleotide sugar metabolism | 13 | 55 | 0.031 | ko00520 | *NANS, TSTA3, RENBP, PGM2, UXS1, GNPNAT1, GALE, PGM1, AMDHD2, GNPDA1, PMM1, GPI, HK1* |

KEGG = the Kyoto Encyclopedia of Genes and Genomes; DEGs = differentially expressed genes.

Table S6 List of differentially expressed miRNAs.

| ID | NCF1 | NCF2 | NCF3 | NCF4 | NCF5 | NCF6 | NCF7 | NCF8 | DCF1 | DCF2 | DCF3 | DCF4 | DCF5 | DCF6 | DCF7 | DCF8 | log2(FC) | *P*-value |
| --- | --- | --- | --- | --- | --- | --- | --- | --- | --- | --- | --- | --- | --- | --- | --- | --- | --- | --- |
| miR-745-3p | 0.01 | 0.14 | 0.01 | 0.01 | 0.01 | 0.01 | 0.01 | 0.01 | 0.01 | 0.01 | 4.86 | 24.24 | 2.83 | 8.82 | 11.57 | 22.00 | 0.02 | <0.001 |
| miR-2944-3p | 0.01 | 0.01 | 0.01 | 0.01 | 0.17 | 0.01 | 0.01 | 0.01 | 0.01 | 0.01 | 4.23 | 19.13 | 2.21 | 6.12 | 7.28 | 21.88 | 0.02 | <0.001 |
| miR-584-5p | 0.01 | 0.01 | 0.01 | 0.01 | 0.01 | 0.01 | 0.01 | 0.01 | 0.01 | 0.01 | 1.44 | 1.70 | 1.06 | 2.16 | 0.44 | 2.99 | 0.01 | <0.001 |
| miR-363-3p | 2.98 | 1.09 | 2.14 | 1.51 | 2.15 | 2.89 | 2.22 | 2.38 | 2.22 | 3.90 | 9.00 | 7.58 | 9.20 | 11.26 | 8.16 | 24.19 | 2.17 | <0.001 |
| miR-71-5p | 0.56 | 0.61 | 0.01 | 0.01 | 0.17 | 0.01 | 0.01 | 0.01 | 0.57 | 0.01 | 4.59 | 20.83 | 3.80 | 8.28 | 8.38 | 27.76 | 0.17 | <0.001 |
| miR-629-5p | 0.01 | 0.01 | 0.01 | 0.01 | 0.01 | 0.01 | 0.01 | 0.01 | 0.01 | 0.01 | 1.71 | 1.70 | 0.27 | 2.07 | 0.33 | 8.29 | 0.01 | <0.001 |
| chi-miR-146b-5p | 52.59 | 70.36 | 66.70 | 70.85 | 76.11 | 100.97 | 91.53 | 79.87 | 116.40 | 130.30 | 162.37 | 125.85 | 240.75 | 229.35 | 182.21 | 109.19 | 76.12 | <0.001 |
| miR-965-3p | 0.40 | 0.20 | 0.17 | 0.01 | 0.25 | 0.01 | 0.01 | 0.01 | 0.01 | 0.01 | 2.52 | 8.43 | 2.56 | 3.87 | 4.85 | 13.48 | 0.13 | <0.001 |
| miR-486-5p | 370.73 | 266.77 | 433.09 | 453.83 | 405.33 | 509.49 | 302.76 | 385.58 | 264.97 | 216.40 | 1584.06 | 2542.11 | 855.93 | 1381.86 | 871.46 | 8941.89 | 390.95 | 0.001 |
| miR-3615-3p | 1.05 | 0.48 | 0.34 | 0.42 | 0.58 | 0.14 | 0.62 | 0.22 | 0.50 | 0.89 | 6.93 | 2.84 | 1.06 | 3.24 | 1.32 | 6.33 | 0.48 | 0.001 |
| chi-miR-146a | 335.62 | 341.14 | 309.53 | 263.68 | 347.82 | 489.50 | 267.45 | 295.98 | 726.85 | 482.00 | 835.86 | 786.45 | 890.14 | 733.97 | 484.56 | 259.84 | 331.34 | 0.004 |
| miR-995-3p | 0.01 | 0.01 | 0.17 | 0.01 | 1.74 | 0.01 | 0.01 | 0.01 | 0.01 | 0.01 | 8.37 | 34.19 | 4.95 | 11.44 | 13.23 | 45.61 | 0.24 | 0.004 |
| miR-2411-3p | 9.10 | 2.38 | 1.63 | 3.86 | 8.35 | 1.80 | 2.91 | 3.46 | 8.88 | 17.79 | 59.49 | 38.45 | 6.37 | 4.59 | 5.18 | 6.33 | 4.19 | 0.006 |
| miR-3162-3p | 2.26 | 0.54 | 0.26 | 0.59 | 0.66 | 0.58 | 0.28 | 0.58 | 0.50 | 6.78 | 11.70 | 0.95 | 0.97 | 1.89 | 2.98 | 2.99 | 0.72 | 0.009 |
| miR-11985-3p | 9.58 | 2.99 | 1.80 | 1.85 | 10.49 | 1.73 | 4.43 | 4.97 | 4.80 | 13.00 | 69.30 | 2.94 | 6.72 | 14.14 | 26.45 | 16.12 | 4.73 | 0.013 |
| miR-10400-5p | 36.40 | 7.41 | 8.73 | 8.23 | 18.26 | 9.17 | 11.42 | 11.75 | 15.47 | 35.79 | 123.75 | 23.96 | 21.31 | 25.12 | 37.92 | 35.01 | 13.92 | 0.013 |
| miR-735-3p | 4.43 | 0.48 | 0.77 | 0.59 | 1.98 | 0.14 | 0.83 | 1.37 | 0.79 | 4.86 | 33.75 | 1.89 | 2.92 | 2.52 | 6.17 | 4.61 | 1.32 | 0.018 |
| miR-492-5p | 1.53 | 0.27 | 0.43 | 0.01 | 0.17 | 0.14 | 0.01 | 0.36 | 0.93 | 1.71 | 5.67 | 1.23 | 1.15 | 0.99 | 2.31 | 1.27 | 0.36 | 0.019 |
| chi-miR-1388-5p | 81.27 | 89.19 | 67.30 | 75.13 | 63.55 | 82.21 | 78.09 | 52.04 | 114.40 | 123.26 | 125.91 | 160.98 | 150.57 | 105.54 | 109.35 | 75.67 | 73.60 | 0.021 |
| miR-1285-3p | 150.21 | 23.25 | 22.52 | 16.79 | 144.37 | 12.99 | 49.99 | 63.58 | 68.48 | 215.37 | 875.37 | 46.40 | 61.98 | 117.60 | 346.34 | 214.58 | 60.46 | 0.024 |
| miR-1388-5p | 18.85 | 24.00 | 15.50 | 16.96 | 20.08 | 22.74 | 20.84 | 14.99 | 36.60 | 31.48 | 29.61 | 30.87 | 41.82 | 23.77 | 36.04 | 20.73 | 19.24 | 0.027 |
| miR-1292-3p | 2.01 | 0.61 | 0.69 | 0.92 | 1.07 | 0.14 | 0.42 | 0.43 | 1.07 | 1.64 | 9.00 | 0.76 | 1.86 | 2.34 | 2.43 | 2.07 | 0.79 | 0.042 |
| miR-10225-3p | 40.35 | 30.59 | 25.17 | 38.11 | 38.84 | 24.40 | 38.15 | 31.64 | 89.97 | 22.45 | 67.86 | 63.54 | 39.52 | 59.70 | 69.66 | 54.02 | 33.41 | 0.045 |

NCF = normal colostrum feeding; DCF = delayed colostrum feeding; FC = fold change.

Table S7 KEGG enrichment analysis of target genes.

| Pathway | Enrich (9617) | All (15069) | Adjusted *P*-value | Pathway ID |
| --- | --- | --- | --- | --- |
| Wnt signaling pathway | 342 | 404 | <0.001 | ko04310 |
| Neurotrophin signaling pathway | 276 | 331 | <0.001 | ko04722 |
| Cell adhesion molecules | 307 | 379 | <0.001 | ko04514 |
| cAMP signaling pathway | 378 | 480 | <0.001 | ko04024 |
| MAPK signaling pathway - fly | 192 | 226 | <0.001 | ko04013 |
| Signaling pathways regulating pluripotency of stem cells | 263 | 326 | <0.001 | ko04550 |
| Ras signaling pathway | 438 | 572 | <0.001 | ko04014 |
| Axon guidance | 410 | 535 | <0.001 | ko04360 |
| Parathyroid hormone synthesis, secretion and action | 235 | 293 | <0.001 | ko04928 |
| Sphingolipid signaling pathway | 230 | 287 | <0.001 | ko04071 |
| Relaxin signaling pathway | 219 | 273 | <0.001 | ko04926 |
| Dopaminergic synapse | 277 | 355 | <0.001 | ko04728 |
| MAPK signaling pathway | 575 | 784 | <0.001 | ko04010 |
| Longevity regulating pathway - mammal | 184 | 226 | <0.001 | ko04211 |
| Cholinergic synapse | 222 | 280 | <0.001 | ko04725 |
| AMPK signaling pathway | 251 | 321 | <0.001 | ko04152 |
| cGMP - PKG signaling pathway | 301 | 392 | <0.001 | ko04022 |
| T cell receptor signaling pathway | 193 | 241 | <0.001 | ko04660 |
| ErbB signaling pathway | 213 | 269 | <0.001 | ko04012 |
| Rap1 signaling pathway | 411 | 552 | <0.001 | ko04015 |
| Insulin signaling pathway | 256 | 330 | <0.001 | ko04910 |
| Adherens junction | 207 | 262 | <0.001 | ko04520 |
| Phosphatidylinositol signaling system | 212 | 269 | <0.001 | ko04070 |
| Hippo signaling pathway | 320 | 423 | <0.001 | ko04390 |
| Endocytosis | 456 | 620 | <0.001 | ko04144 |
| Inositol phosphate metabolism | 150 | 184 | <0.001 | ko00562 |
| Wnt signaling pathway | 342 | 404 | <0.001 | ko04310 |
| Oxytocin signaling pathway | 297 | 391 | <0.001 | ko04921 |
| Melanogenesis | 213 | 272 | <0.001 | ko04916 |
| Phospholipase D signaling pathway | 298 | 393 | <0.001 | ko04072 |
| Pathway | Enrich (9617) | All (15069) | Adjusted *P*-value | Pathway ID |
| FoxO signaling pathway | 219 | 282 | <0.001 | ko04068 |
| Glucagon signaling pathway | 212 | 273 | <0.001 | ko04922 |
| Phototransduction - fly | 80 | 92 | <0.001 | ko04745 |
| Hedgehog signaling pathway | 122 | 149 | <0.001 | ko04340 |
| TNF signaling pathway | 197 | 254 | <0.001 | ko04668 |
| Insulin secretion | 188 | 242 | <0.001 | ko04911 |
| Autophagy - animal | 259 | 345 | <0.001 | ko04140 |
| Thyroid hormone signaling pathway | 215 | 282 | <0.001 | ko04919 |
| Hedgehog signaling pathway - fly | 88 | 105 | <0.001 | ko04341 |
| Circadian entrainment | 190 | 247 | <0.001 | ko04713 |
| Cellular senescence | 257 | 343 | <0.001 | ko04218 |
| Mitophagy - animal | 129 | 162 | <0.001 | ko04137 |
| Gastric acid secretion | 144 | 183 | <0.001 | ko04971 |
| Long-term potentiation | 161 | 207 | <0.001 | ko04720 |
| Tight junction | 340 | 468 | <0.001 | ko04530 |
| Platelet activation | 196 | 259 | <0.001 | ko04611 |
| B cell receptor signaling pathway | 136 | 174 | <0.001 | ko04662 |
| Adrenergic signaling in cardiomyocytes | 285 | 389 | <0.001 | ko04261 |
| Hippo signaling pathway - multiple species | 72 | 86 | <0.001 | ko04392 |
| Sphingolipid metabolism | 82 | 100 | <0.001 | ko00600 |
| Apoptosis - fly | 116 | 148 | <0.001 | ko04214 |
| Regulation of actin cytoskeleton | 382 | 536 | 0.001 | ko04810 |
| Lysine degradation | 132 | 172 | 0.001 | ko00310 |
| Glutamatergic synapse | 209 | 283 | 0.001 | ko04724 |
| Oocyte meiosis | 223 | 304 | 0.001 | ko04114 |
| Glycosaminoglycan biosynthesis - heparan sulfate/heparin | 44 | 51 | 0.001 | ko00534 |
| SNARE interactions in vesicular transport | 50 | 59 | 0.001 | ko04130 |
| Longevity regulating pathway - multiple species | 107 | 139 | 0.002 | ko04213 |
| C-type lectin receptor signaling pathway | 188 | 256 | 0.003 | ko04625 |
| Aldosterone synthesis and secretion | 191 | 261 | 0.003 | ko04925 |
| Focal adhesion | 354 | 502 | 0.003 | ko04510 |
| Pathway | Enrich (9617) | All (15069) | Adjusted *P*-value | Pathway ID |
| Renin secretion | 131 | 174 | 0.003 | ko04924 |
| Cortisol synthesis and secretion | 124 | 164 | 0.003 | ko04927 |
| Fc gamma R-mediated phagocytosis | 182 | 248 | 0.003 | ko04666 |
| Apoptosis - multiple species | 73 | 92 | 0.003 | ko04215 |
| Th1 and Th2 cell differentiation | 134 | 179 | 0.004 | ko04658 |
| Inflammatory mediator regulation of TRP channels | 194 | 267 | 0.005 | ko04750 |
| Osteoclast differentiation | 196 | 270 | 0.005 | ko04380 |
| PI3K-Akt signaling pathway | 530 | 770 | 0.005 | ko04151 |
| Toll-like receptor signaling pathway | 153 | 208 | 0.006 | ko04620 |
| Vasopressin-regulated water reabsorption | 87 | 113 | 0.006 | ko04962 |
| Toll and Imd signaling pathway | 94 | 123 | 0.006 | ko04624 |
| Dorso-ventral axis formation | 49 | 60 | 0.007 | ko04320 |
| Glycosphingolipid biosynthesis - lacto and neolacto series | 56 | 70 | 0.008 | ko00601 |
| p53 signaling pathway | 139 | 189 | 0.009 | ko04115 |
| Calcium signaling pathway | 307 | 438 | 0.009 | ko04020 |
| GnRH signaling pathway | 180 | 250 | 0.011 | ko04912 |
| Glycosaminoglycan biosynthesis - chondroitin sulfate/dermatan sulfate | 36 | 43 | 0.011 | ko00532 |
| Adipocytokine signaling pathway | 139 | 190 | 0.012 | ko04920 |
| Protein processing in endoplasmic reticulum | 234 | 332 | 0.017 | ko04141 |
| TGF-beta signaling pathway | 119 | 163 | 0.024 | ko04350 |
| GABAergic synapse | 136 | 188 | 0.024 | ko04727 |
| Regulation of lipolysis in adipocyte | 88 | 118 | 0.024 | ko04923 |
| HIF-1 signaling pathway | 171 | 241 | 0.032 | ko04066 |
| Apelin signaling pathway | 224 | 321 | 0.038 | ko04371 |
| Chemokine signaling pathway | 263 | 380 | 0.041 | ko04062 |
| Prolactin signaling pathway | 118 | 164 | 0.046 | ko04917 |

KEGG = the Kyoto Encyclopedia of Genes and Genomes; cAMP = cyclic adenosine monophosphate; MAPK = mitogen-activated protein kinase; AMPK = adenosine 5‘-monophosphate (AMP)-activated protein kinase; cGMP-PKG = cyclic guanosine monophosphate- protein kinases G; FoxO = the forkhead box O; TNF = tumor necrosis factor; SNARE = soluble-N-ethylmaleimide-sensitive-factor accessory-protein receptor; TRP = transient receptor potential; PI3K-Akt = phosphatidylinositol 3-kinase- RAC -α serine/threonine-protein kinase; GnRH = gonadotropin-releasing hormone; TGF = transforming growth factor; HIF-1 = hypoxia inducible factor-1.

Supplementary Figures


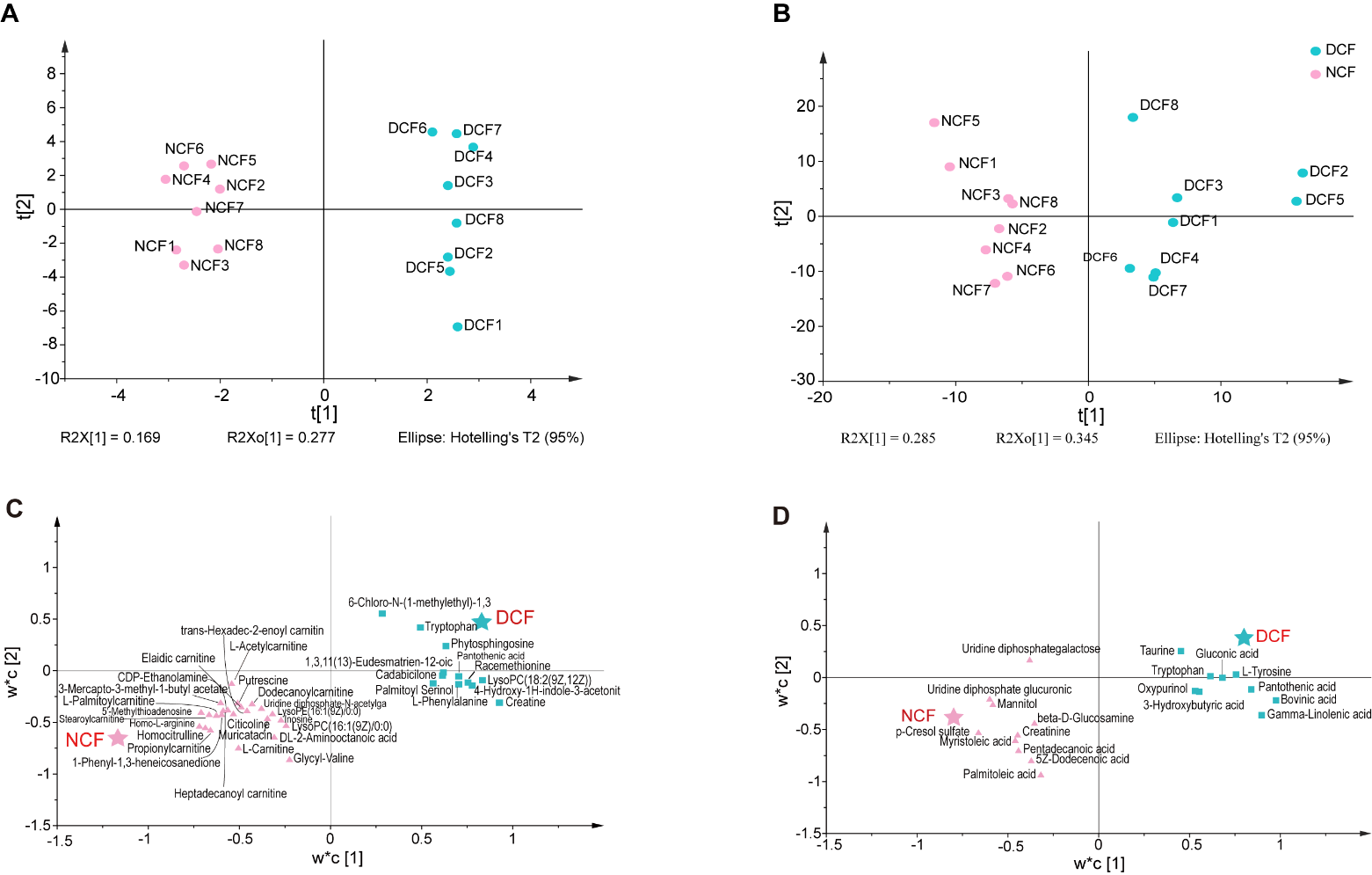


Fig. S1 Plot of orthogonal partial least squares discriminant analysis of all expressed metabolites under (A) positive ion mode and (B) negative ion mode. Loading plot of partial least squares discriminant analysis (PLS-DA) based on of differential metabolites (VIP > 1, *P* < 0.05, |fold change| > 1.2) in (C) positive ion (PI) and (D) negative ion (NI) model, respectively. NCF = normal colostrum feeding; DCF = delayed colostrum feeding; VIP = the variable importance in projection.


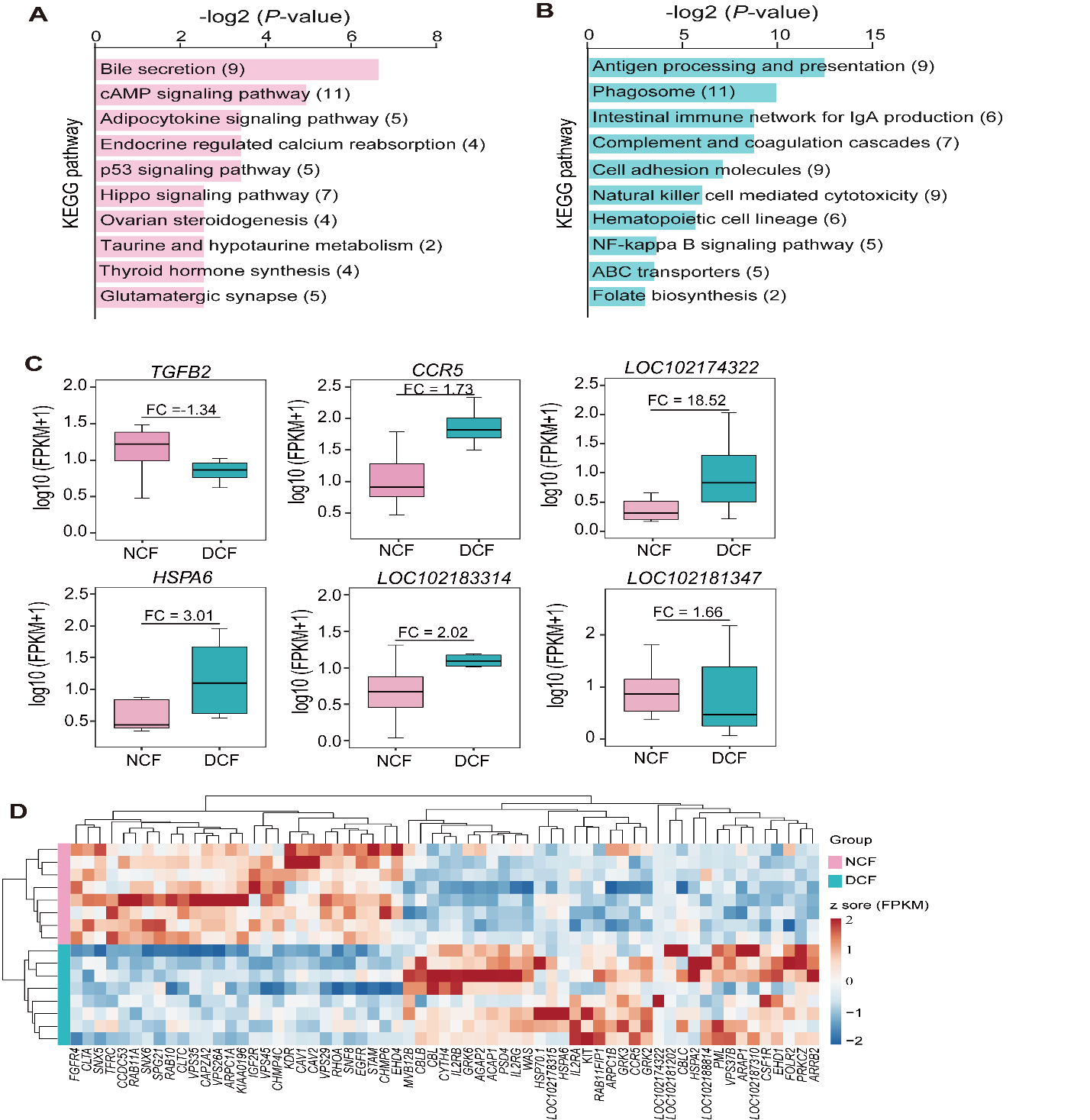


Fig. S2 Transcriptomic analysis of jejunum in neonatal goat kids when subjected to delayed colostrum feeding. Top 10 KEGG pathways of uniquely expressed genes in (A) NCF and (B) DCF group, respectively. Numerical values in parenthesis represents gene number of the pathways. The rank of pathway from top to bottom is according to values of FDR from low to high. (C) Expression (log10 of FPKM+1) and fold change of genes involved in endocytosis uniquely expressed in NCF and DCF group. (D) All DEGs enriched in endocytosis in NCF and DCF group. cAMP = cyclic adenosine monophosphate; IgA = immunoglobulin A; NCF = normal colostrum feeding; DCF= delayed colostrum feeding; FPKM = the fragment per kilobase of transcript per million mapped reads; DEGs = differentially expressed genes; FC = fold change.


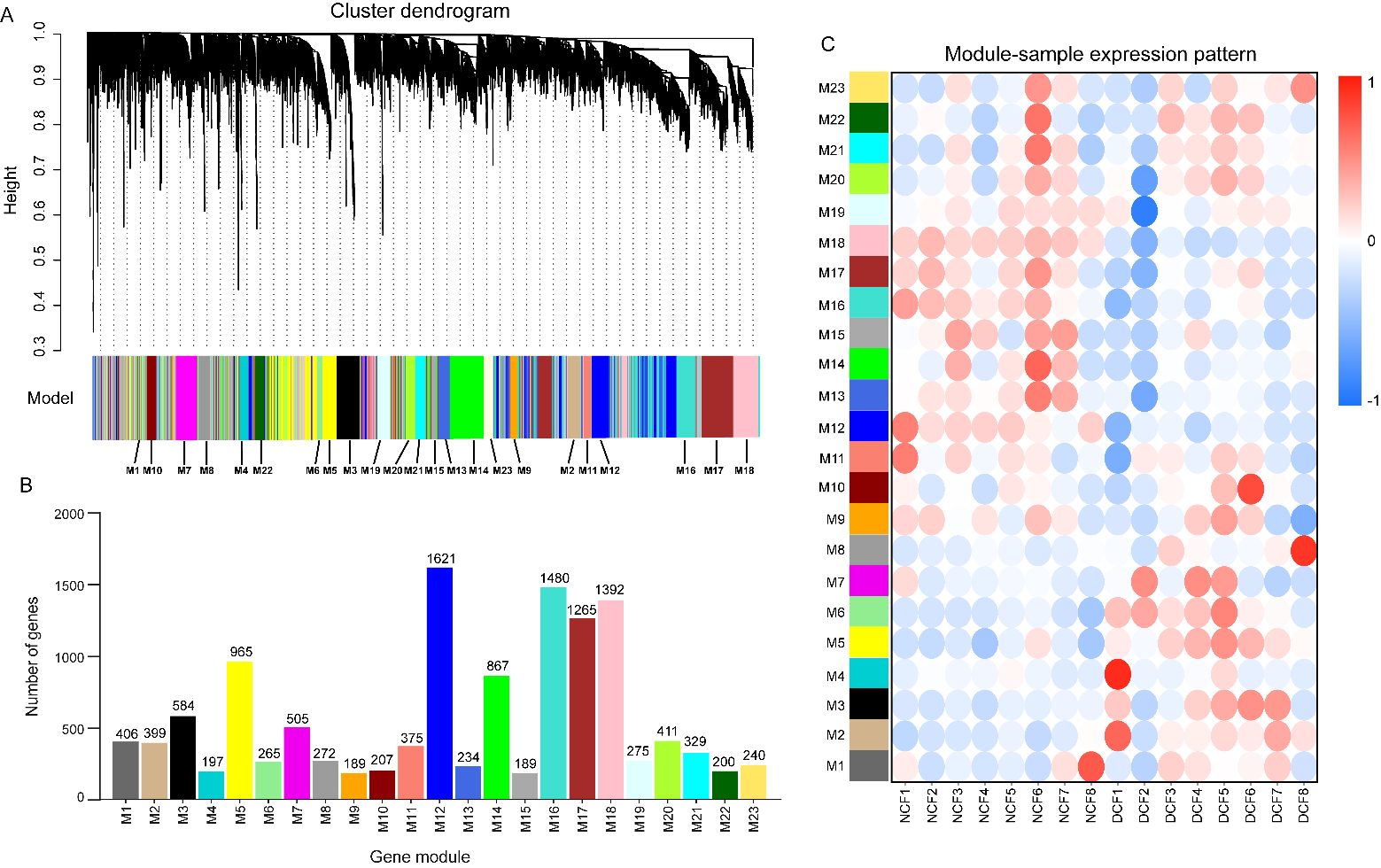


Fig. S3 Global co-expression networks construction and gene modules generation. (A) Cluster dendrogram from gene co-expression network analysis of samples between NCF and DCF. Modules of co-expressed genes were assigned a color and number (M1 to M23). (B) The number of genes in each gene module. (C) Heat map of genes in in each module showing the dynamic expression pattern in NCF and DCF group. The color key of the heat map depicts the z-score of the expression of genes in each module. NCF = normal colostrum feeding; DCF= delayed colostrum feeding.


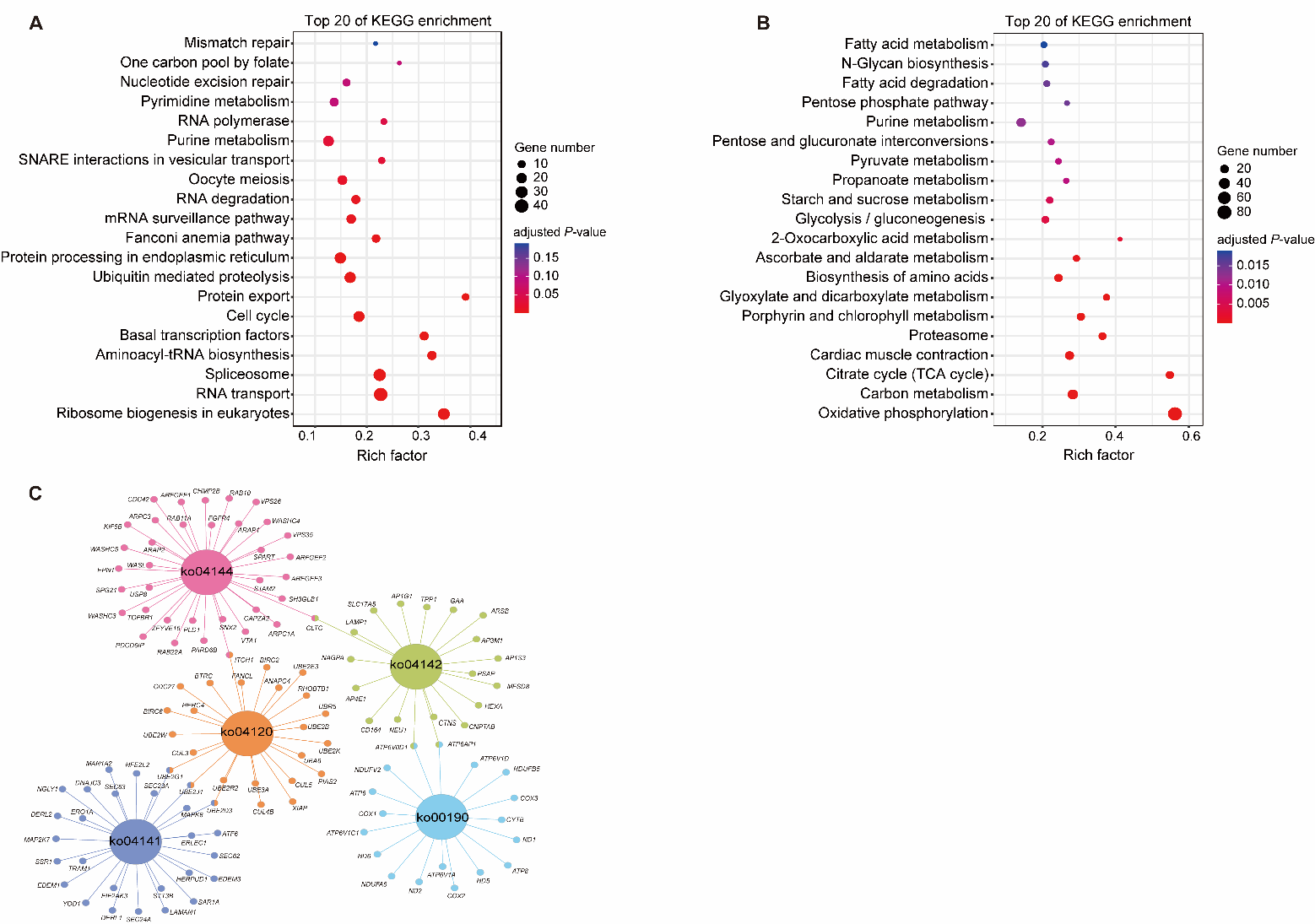


Fig. S4 KEGG pathway enrichment analysis of genes in M12, M16 and M18. Bubble diagram of top 20 KEGG pathways (level 3) of genes co-expressed in M16 (A) and M18 (B) module. (C) Network of genes co-expressed in M12 module and their enriched pathways (level 3) generated by Cytoscape. The ko number ko00190, ko04120, ko04141, ko04142 and ko04144 represents oxidative phosphorylation, ubiquitin mediated proteolysis, protein processing in endoplasmic reticulum, lysosome, endocytosis. KEGG = the Kyoto Encyclopedia of Genes and Genomes; SNARE = soluble-N-ethylmaleimide-sensitive-factor accessory-protein receptor.
